# Organic Selenium Reaches the Central Nervous System and Downmodulates Local Inflammation: A Complementary Therapy for Multiple Sclerosis?

**DOI:** 10.3389/fimmu.2020.571844

**Published:** 2020-10-30

**Authors:** Juliana Helena dos Santos de Toledo, Thais Fernanda de Campos Fraga-Silva, Patrícia Aparecida Borim, Larissa Ragozo Cardoso de Oliveira, Evelyn da Silva Oliveira, Larissa Lucena Périco, Clélia Akiko Hiruma-Lima, Adriana Aparecida Lopes de Souza, Carlos Alberto Ferreira de Oliveira, Pedro de Magalhães Padilha, Marcos Felipe Pinatto-Botelho, Alcindo Aparecido dos Santos, Alexandrina Sartori, Sofia Fernanda Gonçalves Zorzella-Pezavento

**Affiliations:** ^1^Department of Chemical and Biological Sciences, Institute of Biosciences, São Paulo State University (UNESP), Botucatu, Brazil; ^2^Department of Structural and Functional Biology, São Paulo State University (UNESP), Institute of Biosciences, Botucatu, Brazil; ^3^Veterinary Clinical Laboratory, School of Veterinary Medicine and Animal Science (FMVZ), São Paulo State University (UNESP), Botucatu, Brazil; ^4^Department of Research and Development, Biorigin Company, Lençóis Paulista, Brazil; ^5^LabSSeTe Department of Fundamental Chemistry, Institute of Chemistry, University of São Paulo (USP), São Paulo, Brazil

**Keywords:** multiple sclerosis, experimental autoimmune encephalomyelitis, selenium, microglia, inflammasome

## Abstract

Multiple sclerosis (MS) is an inflammatory and demyelinating disease of the central nervous system (CNS). The persistent inflammation is being mainly attributed to local oxidative stress and inflammasome activation implicated in the ensuing demyelination and axonal damage. Since new control measures remain necessary, we evaluated the preventive and therapeutic potential of a beta-selenium-lactic acid derivative (LAD-βSe), which is a source of organic selenium under development, to control experimental autoimmune encephalomyelitis (EAE) that is an animal model for MS. Two EAE murine models: C57BL/6 and SJL/J immunized with myelin oligodendrocyte glycoprotein and proteolipid protein, respectively, and a model of neurodegeneration induced by LPS in male C57BL/6 mice were used. The preventive potential of LAD-βSe was initially tested in C57BL/6 mice, the chronic MS model, by three different protocols that were started 14 days before or 1 or 7 days after EAE induction and were extended until the acute disease phase. These three procedures were denominated preventive therapy −14 days, 1 day, and 7 days, respectively. LAD-βSe administration significantly controlled clinical EAE development without triggering overt hepatic and renal dysfunction. In addition of a tolerogenic profile in dendritic cells from the mesenteric lymph nodes, LAD-βSe also downregulated cell amount, activation status of macrophages and microglia, NLRP3 (NOD-like receptors) inflammasome activation and other pro-inflammatory parameters in the CNS. The high Se levels found in the CNS suggested that the product crossed the blood-brain barrier having a possible local effect. The hypothesis that LAD-βSe was acting locally was then confirmed by using the LPS-induced neurodegeneration model that also displayed Se accumulation and downmodulation of pro-inflammatory parameters in the CNS. Remarkably, therapy with LAD-βSe soon after the first remitting episode in SJL/J mice, also significantly downmodulated local inflammation and clinical disease severity. This study indicates that LAD-βSe, and possibly other derivatives containing Se, are able to reach the CNS and have the potential to be used as preventive and therapeutic measures in distinct clinical forms of MS.

## Introduction

Multiple sclerosis (MS) is a chronic and inflammatory condition of the central nervous system (CNS) whose prevalence is clearly increasing in many countries (1). Symptomatology and patient’s disability are consequences of the presence of demyelinating lesions in the CNS and include weakness, fatigue, incontinence and paralysis (2). The immunopathogenesis of these lesions is complex and involves the interplay of distinct subsets of T lymphocytes. Autoreactive T cells specific for myelin peptides, probably activated in the peripheral lymphoid organs by molecular mimicry or bystander activation, migrate toward the CNS and target the myelin sheath-producing oligodendrocytes, triggering a local inflammatory and injurious process (3, 4). Classically, Th1 and Th17 cells, locally reactivated by myelin presented by microglia act mainly by releasing pro-inflammatory cytokines as IFN-γ, TNF-α, IL-6 and IL-17 (5). Th17 cells have also being associated to blood-brain barrier (BBB) disruption, facilitating therefore lymphocyte transmigration to the CNS (6). Local antigen presentation, that takes place in the presence of MHCII and co-stimulatory molecules, allows T cell reactivation in the CNS and is viewed as a crucial event for disease evolution (7). Different cell types as peripheral dendritic cells (DCs), infiltrating macrophages, CNS-resident microglia, and T lymphocytes (Th1, Th17, and Tregs) have been extensively investigated concerning their contribution to this neuroinflammatory process (8, 9).

This orchestrated cell interplay will, ultimately, generate inflammation and subsequent neurodegeneration. Strong evidences indicate that oxidative stress is a dominant trigger of inflammation and vice versa and that this cyclical process is implicated in both, demyelination and axonal damage processes (10). A close relationship between the oxidative stress and the activation of the inflammasome platform has been described. CNS reactive species of oxygen (ROS) are mostly produced by infiltrating macrophages and activated microglia. In addition to direct damage to the BBB and myelin sheath (11, 12), ROS are also considered the main inflammasome activators (13). The inflammasomes are innate system receptors that perceive inflammatory signals coming from infections or from host derived molecules. Several inflammasomes have been identified so far but NOD-like receptor pyrin/domain-containing-3 (NLRP3) inflammasome is currently the best characterized complex involved in the pathogenesis of chronic inflammatory and autoimmune conditions including MS (14). Briefly, pathogen-associated molecular patterns (PAMPs) or damage-associated molecular patterns (DAMPs) interaction with Toll-like receptors (TLRs) triggers NF-κB activation, promoting the transcription of NLRP3, pro-IL-1β, and pro-IL-18. Subsequent stimuli as presence of ROS, potassium efflux, calcium influx or mitochondrial damage, allows oligomerization of the inflammasome complex and subsequent release of active IL-1β and IL-18 (15). In addition of being essential mediators of innate immunity, IL-1β and IL-18 play important roles instructing Th1 and Th17-types of polarization (15).

Although there is no cure for MS yet, the current therapy, which is essentially based on the application of immunomodulatory drugs, can reduce the intensity and number of disease relapses (16, 17). Alternative and adjunct therapies have been largely investigated, including compounds derived from helminths (18), medical cannabis (19) and vitamin D (20). In the last few years our research group has been using experimental autoimmune encephalomyelitis (EAE) to investigate alternative or adjunct therapies for MS (21–23). EAE is used worldwide as a study tool to allow a deeper comprehension of MS immunopathogenesis. C57BL/6 mice develop a chronic type of disease characterized by only one peak of CNS inflammation and demyelination, whereas SJL/J mice mimics the relapsing-remitting MS (RRMS), featured by periods of stability in between relapses. Notably, around 85% of the patients develop this type of disease (24). Both murine models are largely employed to study new prophylactic and therapeutic approaches, but SJL/J mice also contributes to the development of drugs to reduce the intensity or frequency of relapses (25).

Many of the drugs currently elected for MS therapy were assayed and validated by pre-clinical investigations performed with the EAE model (25). Despite the variety of medications, some patients do not respond to available therapies and the global inhibition of the immune system can trigger collateral effects as increased susceptibility to infections (26).

In the light of MS immunopathogenesis, a product with the ability to control ROS production and/or inflammasome activation and capable of reaching the CNS seemed logical to be tested. From this perspective, selenium (Se) is a micronutrient essential for normal physiological processes that is endowed with antioxidant and anti-inflammatory properties (27). It is involved in the mitochondrial dynamics, calcium channels and free radical regulation, that are all pathways implicated in MS evolution (11, 27, 28). In addition, Se supplementation can induce a regulatory phenotype in Th cells (29), reduce the expression of adhesion factors as E-selectin (30) and polarize macrophages to an anti-inflammatory profile (31). Se supplementation has already been effective in the control of Parkinson and other possible autoimmune conditions (11, 32, 33). Its beneficial effects depend mainly on its incorporation into proteins that will mediate the activation, proliferation and differentiation of innate and adaptative immune components (11, 29). Se-containing amino acids, such as selenocysteine and selenium-methionine, can provide direct antioxidant benefits and can also be incorporated into the synthesis of antioxidant enzymes, as glutathione peroxidase, thioredoxin reductases and methionine sulfoxide reductases (28). Synthetic compounds containing organic Se mimic the effects of human selenized proteins, including antioxidant activity (34).

In this context, we showed that a beta-selenium-lactic acid derivative (LAD-βSe) was able to significantly control the clinical development of the two most employed murine experimental models of MS. We also found that this product reaches the CNS and downmodulates pivotal parameters locally involved in neurodegeneration.

## Materials and Methods

### Experimental Design

In this study were used two different murine models to evaluate the preventive and therapeutic potential of LAD-βSe to control EAE development: C57BL/6 mice as a chronic MS model and SJL/J as a relapsing-remitting one. The preventive potential of LAD-βSe was tested in C57BL/6 mice by three different protocols that started 14 days before, and 1 or 7 days after EAE induction and extended until the acute disease phase (day 18). In this case, the animals were allocated into five groups: Control (normal healthy mice injected with the vehicle); Control/EAE (non-treated sick group injected with the vehicle); LAD-βSe -14 days/EAE (sick group treated with LAD-βSe starting 14 days before EAE induction); LAD-βSe 1 day/EAE (sick group treated with LAD-βSe starting 1 day after EAE induction); LAD-βSe 7 days/EAE (sick group treated with LAD-βSe starting 7 days after EAE induction). Weight and clinical score were evaluated daily whereas the other analyses were done at the acute disease phase which occurred at the 18th day after disease induction. Serum samples were employed for biochemical analysis of hepatic and renal function, mesenteric lymph nodes for Foxp3+ Treg cells frequency and maturation status of dendritic cells (DCs), and CNS for oxidative stress, selenium levels, macrophage/microglial cells evaluation and inflammatory parameters. In the relapsing-remitting murine model, SJL/J mice were treated with LAD-βSe soon after the first remitting episode and the analyzes were done 42 days after EAE induction. The animals were allocated into three groups: Control (normal healthy mice injected with the vehicle); Control/EAE (non-treated sick group injected with the vehicle); LAD-βSe/EAE (sick group treated with LAD-βSe starting 20 days after EAE induction). Weight and clinical score were checked daily whereas oxidative stress and inflammatory parameters in the CNS were evaluated at the relapsing peak observed 42 days after disease induction. A model of neurodegeneration induced by LPS in male C57BL/6 mice was also employed. In these experiments, the animals were allocated into three groups: Control (normal healthy mice injected with the vehicle); LPS (non-treated group injected with two doses of LPS); LPS/LAD-βSe (group injected with two doses of LPS and treated with sevens doses of LAD-βSe starting 5 days before LPS administration). One day after last LPS administration, we evaluated the oxidative stress, selenium levels, and inflammatory parameters at the CNS. All these pro-inflammatory parameters were determined only by real time RT-PCR, that is, they were not complemented by evaluation of the corresponding proteins, constituting, therefore, a limitation of this aspect of this investigation.

### Mice

Male and female C57BL/6 mice with 8–9 weeks old were purchased from the University of São Paulo (USP, Ribeirão Preto, SP, Brazil) and female SJL/J mice with 10–12 weeks old were breed in the Animal Facility of the Department of Chemical and Biological Sciences (UNESP, Botucatu, SP, Brazil), according to the local Ethics Committee on Use of Animals (CEUA - protocol number 1016/2017). Mice were maintained in a specific pathogen-free facility and received sterilized food and water *ad libitum*. Animals were anesthetized with ketamine/xylazine and then perfused with 10 ml of saline solution before collecting biological samples, except in the case of blood samples that were withdraw before perfusion. All experiments were done in accordance with the local CEUA (protocol number 1114/2018).

### Beta-Selenium-Lactic Acid Derivative

The LAD-βSe is being developed by LabSSeTe/IQ-USP (São Paulo/SP, Brazil) and Biorigin Company (Lençóis Paulista/SP, Brazil). LAD-βSe synthesis was conducted as the patent WO 2015/155453A2 (**Supplementary File**).

### EAE Induction

Female C57BL/6 mice (chronic model) and female SJL/J mice (relapsing-remitting model) were subcutaneously injected, at the lower back, with 100µg of MOG_35–55_ (MEVGWYRSPFSRVVHLYRNGK) or 100 μg of PLP_139–151_ (HCLGKWLGHPDKF) peptides (both from Proteimax, São Paulo, SP, Brazil). These peptides were previously emulsified with 25µl of Complete Freund’s Adjuvant (CFA) (Sigma-Aldrich, St. Louis, MO, USA) containing 2 mg/ml of* Mycobacterium tuberculosis* (Difco, Detroit, MI, USA). Mice also received two intraperitoneal doses of *Bordetella pertussis* toxin (200 ng/Sigma-Aldrich), 0 and 48 h after immunization. Control group without EAE was not injected with CFA and pertussis toxin. Clinical score and body weight were assessed daily. Clinical scores in the chronic EAE model were defined according to the following degrees of paralysis: 0- no symptoms, 1- limp tail, 2- hind legs weakness, 3- partially paralyzed hind legs, 4- complete hind leg paralysis, and 5- complete paralysis. Relapsing-remitting disease was analyzed according to the following scores: 0- no symptoms, 0.5- weak tail tip, 1- limp tail, 1.5- limp tail + one weak hind limb, 2- limp tail + two weak hind limbs, 2.5- limp tail + paralysis of one hind limb, 3- complete hind limb paralysis, 3.5- complete hind limb paralysis + one weak forelimb, 4- complete hind limb paralysis and beginning front limb paralysis, 4.5- complete hind limb paralysis + weak forelimbs, 5- moribund or dead.

### Preventive/Therapeutic Procedures

The product tested in this investigation is a LAD-βSe, that is being developed by LabSSeTe/IQ-USP (São Paulo/SP, Brazil) and Biorigin Company (Lençóis Paulista/SP, Brazil), in a cooperative program. Mice were treated daily by oral gavage with 0.125 mg of LAD-βSe (diluted in water for injection), containing 45 μg of Se/dose. This Se dose was based on previous data published by Vieira et al. (35). Three procedures were tested in the chronic model: a preventive therapy (LAD-βSe −14 days) approach that included 32 doses, started 14 days before EAE induction and extended up to the EAE acute phase, and a preventive therapy 1 day (16 doses) and a preventive therapy 7 days (10 doses) approaches that started on days 1 and 7 after EAE induction, respectively. The last 2 preventive therapies were also extended up to the acute disease phase (18 days after MOG injection). SJL/J mice treatment (22 doses) was initiated on day 20 which corresponds to the first remitting disease episode and prolonged until day 42. Control groups received the diluent by gavage following the scheme of the different protocols.

### Mesenteric Lymph Node and CNS Mononuclear Cells Isolation

Mesenteric lymph nodes were collected and macerated with cell strainers in HBSS (Hanks’ Balanced Salt solution). After centrifugation at 450 ×g at 4°C for 5 min the cells were resuspended in HBSS, counted and stained as described below to be further analyzed by flow cytometry. To obtain mononuclear cells from the CNS, brain and spinal cord were collected and digested with 2.5 mg/ml of collagenase D (Roche Applied Science, Indianapolis, IN, USA) and DNAse (100µg/ml, Sigma-Aldrich) at 37°C for 45 min. After maceration with cell strainers, suspensions were washed in HBSS and centrifuged at 450 ×g for 7 min at 18°C. Cells were resuspended in Percoll (GE Healthcare, Uppsala, Sweden) 30% and gently placed over Percoll 70%. After centrifugation at 950 ×g for 20 min at 18°C, the ring containing mononuclear cells was collected and washed in HBSS. After a last centrifugation at 450 ×g for 7 min at 18°C, the mononuclear cells were resuspended in HBSS, counted and stained as described below to be further analyzed by flow cytometry.

### Macrophage and Microglial Cells Evaluation

The percentages of infiltrating macrophages/activated microglia (CD45^High^CD11b^+^) and resting microglia (CD45^Low^CD11b^+^) in CNS-isolated mononuclear cells were analyzed by flow cytometry. Samples were incubated with FITC labeled anti-mouse CD45 (30-F11), PerCP-Cy5.5 labeled anti-mouse CD11b (M1/70), APC labeled anti-mouse MHC II (M5/114.15.2) for 30 min at 4°C, according to manufacturer’s instructions (eBiosciences, San Diego, CA, USA). After staining, the cells were washed, resuspended in flow cytometry buffer, and fixed in paraformaldehyde 1%. Flow cytometry was performed using a FACS Canto II (Becton Dickinson - BD, California, EUA) from Institute of Biosciences (UNESP, Botucatu, SP, Brazil) and the data were analyzed with FlowJo software (Becton Dickinson - BD, California, EUA).

### Treg Foxp3+ and Dendritic Cells Evaluation

The percentage of Treg cells (CD3^+^CD4^+^CD25^High^Foxp3^+^), dendritic cells (DCs) (CD11c^+^MHCII^+^) and tolerogenic DCs (CD11b^-^CD103^+^) were evaluated by flow cytometry in cells from mesenteric lymph nodes. Samples were incubated with PerCP-Cy5.5 labeled anti-mouse CD11b (M1/70), APC-Cy7 labeled anti-mouse CD11c (N418), APC labeled anti-mouse MHCII (M5/114.15.2) and PE labeled anti-mouse CD103 (2E7) panel or with PercP 5.5 labeled anti-mouse CD3 (145-2C11), PE-Cy7 labeled anti-mouse CD4 (GK1.5) and APC labeled anti-mouse CD25 (PC61.5) panel for 30 min at 4°C. Intracellular Foxp3 transcription factor was detected using Anti-mouse/rat Foxp3 Staining Set PE (FJK-16s) (eBiosciences, San Diego, CA, USA) according to manufacturer’s instructions. After staining, the cells were washed, resuspended in flow cytometry buffer, and fixed in paraformaldehyde 1%. Flow cytometry was performed as described above. Gate strategy for DCs was based on Ruane and Lavelle (36).

### Reverse Transcriptase PCR Assays

RNA was extracted from lumbar spinal cord and lymphoid tissue (mesenteric and inguinal lymph nodes) with TRIzol reagent (Life Technologies, Austin, TX, USA). One µg of RNA was converted to cDNA using High Capacity cDNA Reverse Transcription Kit (Applied Biosystems, California City, CA, USA) according to the manufacturer’s instructions. Tbet (Mm00450960_m1), GATA-3 (Mm0484783_m1), RORc (Mm01261022_m1), Foxp3 (Mm00475162_m1), CX3CR1 (Mm00438354_m1), iNOS (Mm00440502_m1), Arginase-1 (Mm00475988_m1), NLRP3 (Mm00840904_m1), ASC (Mm00451187_m1), caspase-1 (Mm00438023_m1) and IL-1β (Mm00434228_m1) expression was analyzed in comparison to GAPDH (Mm99999915_g1, housekeeping gene) levels. Real time PCR reactions were performed using TaqMan assays according to manufacturer’s recommendations (Applied Biosystems). Levels of gene expression were represented as relative copy numbers by using the method of delta threshold (2^-ΔΔCt^) according to Livak and Schmittgen (37).

### Hepatic and Renal Function

Concentrations of alanine aminotransferase (ALT), aspartate aminotransferase (AST), gamma-glutamyl transferase (GGT) and alkaline phosphatase (AF) were evaluated in serum samples using commercial biochemistry kits according to manufacturer’s recommendations (Labtest Diagnóstica AS, Vista Alegre, MG, Brazil). Urea and creatinine serum levels were quantified with Bioclin commercial kits (Quibasa Química Básica Ltda, Belo Horizonte, MG, Brazil). Results were measured by Cobas Mira plus Chemistry Analyzer (Roche Diagnostic Systems).

### Oxidative Stress in the Lumbar Spinal Cord

Lumbar spinal cords from C57BL/6 were initially homogenized with RIPA extraction buffer (50 mM HEPES pH 7.9, 1 M KCl, 1 M MgCl_2_, 0.1 M EDTA, 0.1 M NaF) and 1% protease inhibitor cocktail. After homogenization, the samples were centrifuged at 14000 rpm for 45 min at 4°C and the supernatants were collected and maintained at -80° C for the determination of reduced glutathione (GSH), catalase (CAT) and superoxide dismutase (SOD).

#### Determination of SOD

The supernatants were diluted in PBS (0.1 M, pH 7.2) at a ratio of 1:20 (w/v). In 100 μl of the supernatants were added 150 μl of a solution containing hypoxanthine, xanthine oxidase and nitrotetrazolium blue chloride at a ratio of 1:1:1. The absorbance was read in Eon Microplate Spectrophotometer (BioTek Instruments, Winooski, VT, USA) at 560 nm every min for 10 min at 37°C. The mean of the absorbance was divided by the protein concentration and the results were expressed as U of SOD/μg protein (38).

#### Determination of CAT

The supernatants were diluted in KH_2_PO_4_ buffer (25 mM, pH 7.5, 1 mM EDTA, and 1% BSA) at a ratio of 1:20 (w/v). Catalase was added to two wells of the plate and used as a positive control. Twenty μl of the samples were distributed in a 96-well plate with 100 μl of buffer (KH_2_PO_4_, 250 mM, pH 7.0), 30 μl of methanol and 20 μl of H_2_O_2_ (35.3 mM). The plate was held for 20 min at room temperature. After this, 30 μl of KOH (10 mM) and 45 μl of Purpald^®^ were added and the plate was held under gentle agitation for 20 min at room temperature and protected from light. Subsequently, 15 μl of KIO_4_ (65.2 mM) were added and the plate was maintained under gentle stirring for 10 min at room temperature and protected from light. Absorbance determination was performed in Eon Microplate Spectrophotometer (BioTek Instruments) at 540 nm. The concentration average was divided by the protein concentration and the results were expressed as U of CAT/μg protein (39).

#### Determination of Reduced Glutathione Levels (GSH)

The supernatants were diluted in PBS buffer in a ratio of 1:10 (w/v). The GSH levels were determined using 100 μl of Tris/EDTA buffer and 100 μl of samples and a spectrophotometer reading was performed. Then, 20 μl of 5’-5-dithio-bis-2-nitrobenzoic acid (DTNB) was added, incubated at room temperature for 15 min, and a re-reading of absorbance. The intensity of the yellow color was read using Eon Microplate Spectrophotometer (BioTek Instruments) at 412 nm. The concentration average was divided by the protein and the results were expressed in nmol of GSH/μg protein (40).

### Determination of Malondialdehyde Levels

Brain and spinal cord from SJL/J mice (cervical and thoracic portions) were homogenized with KCl 1.15%. After centrifugation at 9000 rpm for 10 min at 4°C, the supernatants were collected and diluted in distilled water, lauryl, acetic acid and thiobarbituric acid. Samples were incubated at 95°C for 1h and then centrifuged at 4000 rpm for 10 min at 25°C. 200 µl de each sample were distributed in 96-well plates. Samples and standard curve were measured in duplicate at 532nm in Eon Microplate Spectrophotometer (BioTek Instruments). Results were expressed in nmol of malondialdehyde level (MDA)/g of tissue (41).

### Determination of Selenium Levels in the CNS

Se levels were determined in lyophilized samples made up of brain plus the cervical and thoracic portions of the spinal cord by a graphite furnace atomic absorption spectrometry. A Shimadzu model AA-6800 atomic absorption spectrometer was used, equipped with a background absorption corrector with a deuterium lamp and self-reverse (SR) system, and a pyrolytic graphite tube with integrated platform and automatic ASC-6100 sampler. A Shimadzu hollow cathode selenium lamp operated with a 10 mA current was also used. The wavelength applied was 196.0 nm and the spectral resolution was 0.5 nm. Argon was employed as inert gas at a constant flow of 1 l min^−1^ throughout the heating program, except during the atomization step, when the gas flow was interrupted. The absorbance signals were measured in the peak area. Sample preparation was done by particulate matter based on (42). After the sonication step of the sample in slurries and/or standard slurries directly in the auto sampler’s containers, 20 μl of standard or sample were injected into the graphite tube (coated internally with metallic tungsten), using the auto sampler’s micropipette according to Silva et al., 2006 (43).

### LPS-Induced Murine Neuroinflammation Model

To trigger LPS neuroinflammation, C57BL/6 male mice received two intraperitoneal doses of LPS (1 mg/kg) (Sigma-Aldrich, serotype 055:B5) as described by Chen et al., 2012 (44). Mice were daily treated by gavage during 5 days before LPS administration with 0.111 mg of LAD-βSe diluted in water for injection, containing 40 μg of Se/dose. The animals also received LAD-βSe during the 2 days of LPS challenge, totalizing seven LAD-βSe doses. A control group received the same diluent by gavage. Twenty-four hours after the last LPS injection, mice were anesthetized with ketamine/xylazine and perfused with 10 ml of saline solution before collection of biological samples. Brain and medulla oblongata were used to analyze the neuroinflammatory process.

### Statistical Analysis

Data were expressed as mean ± standard deviation or as median and interquartile (25%–75%) ranges. To test normality of data, results were analyzed by Shapiro-Wilk’s test. Comparisons between experimental groups were made by t-test or One-way ANOVA followed by Tukey’s test for parametric variables and by Mann-Whitney or Kruskal-Wallis followed by Dunn’s test for nonparametric variables. Chi-square and Fisher Exact Test were performed for EAE prevalence. For comparison of EAE clinical scores between groups, repeated measures two-way ANOVA were performed followed by a Bonferroni *post hoc* test. Statistical analysis was accomplished with SigmaPlot Software Version 12.0 (Systat Software Inc., San Jose, CA, USA) and p < 0.05 was considered significant.

## Results

### Long-Term LAD-βSe Administration Reduces Clinical Severity

**Figure 1:**
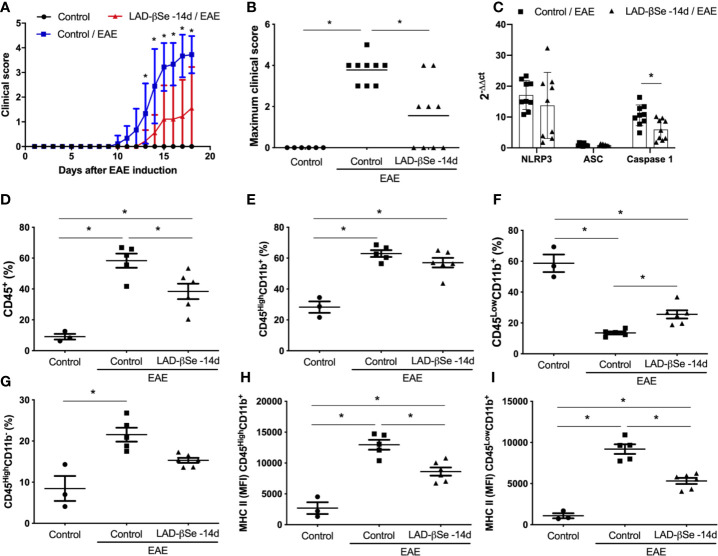
Long-term administration of beta-selenium-lactic acid derivative (LAD-βSe) controls chronic experimental autoimmune encephalomyelitis (EAE) clinical development: effect on inflammasome, infiltrating macrophages and microglia. Daily clinical score **(A)** and maximum clinical score **(B)**. NOD-like receptor pyrin/domain-containing-3 (NLRP3), ASC, and Caspase-1 transcripts **(C)** in the central nervous system (CNS) were analyzed by real-time RT-PCR during EAE acute phase (day 18). The levels of mRNA expression were represented by the values of 2^-ΔΔct^. The values obtained in the CNS analysis of healthy mice were used as reaction calibrators. The percentage of infiltrating leukocytes plus microglia (CD45^+^) **(D)**, macrophages/activated microglia (CD45^High^CD11b^+^) **(E)**, resting microglia (CD45^Low^CD11b^+^) **(F)** and lymphocytes (CD45^High^CD11b^-^) **(G)** cells eluted from the CNS were analyzed by flow cytometry. Mean fluorescence intensity (MFI) of the MHCII on macrophages/activated microglia **(H)** and resting microglial cells **(I)**. Results represent the median ± SEM of three–six animals per group in each experiment and are representative of two independent experiments. *p < 0.05.

In a preliminary assay we tested the potential of LAD-βSe to control disease evolution. To investigate this possibility, mice were injected with LAD-βSe during 32 consecutive days beginning 14 days before and ending 18 days after EAE induction. LAD-βSe administration significantly controlled disease severity as indicated by the evident reduction in the clinical score (**Figures 1A, B**). This effect was associated with a discrete alteration in the inflammasome platform characterized by normal NLRP3 and ASC mRNA levels but significantly decreased levels of caspase-1 mRNA, as shown in **Figure 1C**. In terms of percentage, LAD-βSe administration significantly reduced the amount of CD45^+^ cells (infiltrating leukocytes plus microglia) and slightly reduced CD45^High^CD11b^-^ (lymphocytes) as showed in **Figures 1D, G**, respectively. The percentage of CD45^High^CD11b^+^ (infiltrating macrophages plus activated microglia) was not altered and of CD45^Low^CD11b^+^ (resting microglia) was increased, as illustrated at **Figures 1E, F**, respectively. These two phagocytic cell populations displayed reduced expression of MHC II (**Figures 1H, I**).

### LAD-βSe Administration as a Preventive Therapy 1 Day or 7 Days Controls EAE Development in C57BL/6 Mice Without Causing Side Effects

**Figure 2:**
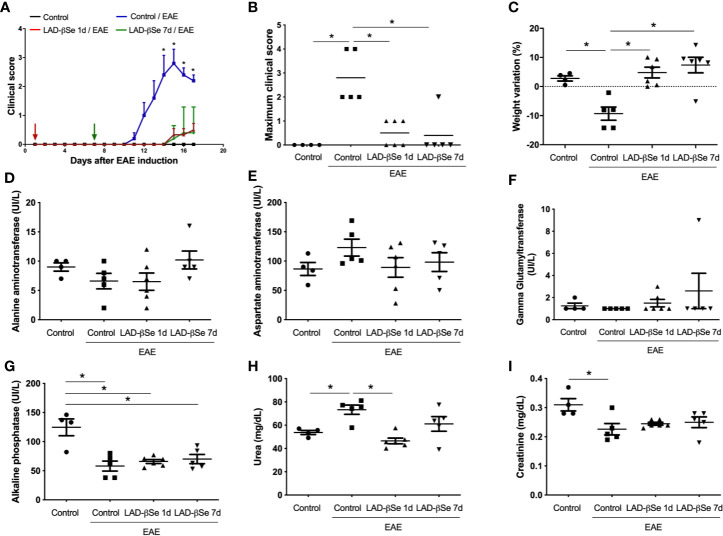
Therapeutic control of chronic experimental encephalomyelitis by administration of beta-selenium-lactic acid derivative (LAD-βSe) displays no side effects. Daily clinical score **(A)** and maximum clinical score **(B)**. Serum quantification of alanine aminotransferase (ALT) **(D)**, aspartate aminotransferase (AST) **(E)**, gamma-glutamyl transferase (GGT) **(F)**, alkaline phosphatase **(G)**, urea **(H)** and creatinine **(I)** was performed by biochemical assays during EAE acute phase (day 18). Body weight changes were determined by comparing the weight observed at the acute phase and at the day of experimental autoimmune encephalomyelitis (EAE) induction **(C)**. Results represent the mean ± SEM of four–six animals per group in each experiment and are representative of 4 independent experiments. *p < 0.05.

A foul breath odor in the animals´ cages during the initial long-term protocol suggested that an excess of Se was being administered to the animals. Two preventive therapeutic procedures comprising a smaller number of doses were therefore tested and initiated 1 and 7 days after EAE induction. Both strategies significantly controlled EAE progress as indicated by reduced clinical scores (**Figures 2A, B**). LAD-βSe treatment did not affect the usual feed consumption (not shown) and prevented weight loss (**Figure 2C**).

Assays to evaluate hepatic and renal functions revealed that treatments were devoid of side effects. Despite some fluctuations, the levels of alanine (**Figure 2D**) and aspartate aminotransferases (**Figure 2E**) were comparable in the 4 experimental groups. An upward trend was detected in the levels of gamma glutamyl transferase in mice treated from the 7th day on (**Figure 2F**). Mice with EAE presented significantly lower levels of alkaline phosphatase than normal mice and these lower levels were kept in treated mice (**Figure 2G**). Urea and creatinine were quantified to investigate possible kidney toxicity. Urea levels were significantly elevated in EAE mice compared to control animals. The treatment started on day 1 normalized urea levels whereas treatment started on day 7 triggered just a trend to normalization (**Figure 2H**). Creatinine levels were downmodulated in EAE mice; treatment started on day 1 normalized its levels and treatment started on day 7 showed a trend to normalization (**Figure 2I**).

### LAD-βSe Administration as a Preventive Therapy 1 Day or 7 Days Tolerizes DCs From Mesenteric Lymph Nodes

**Figure 3:**
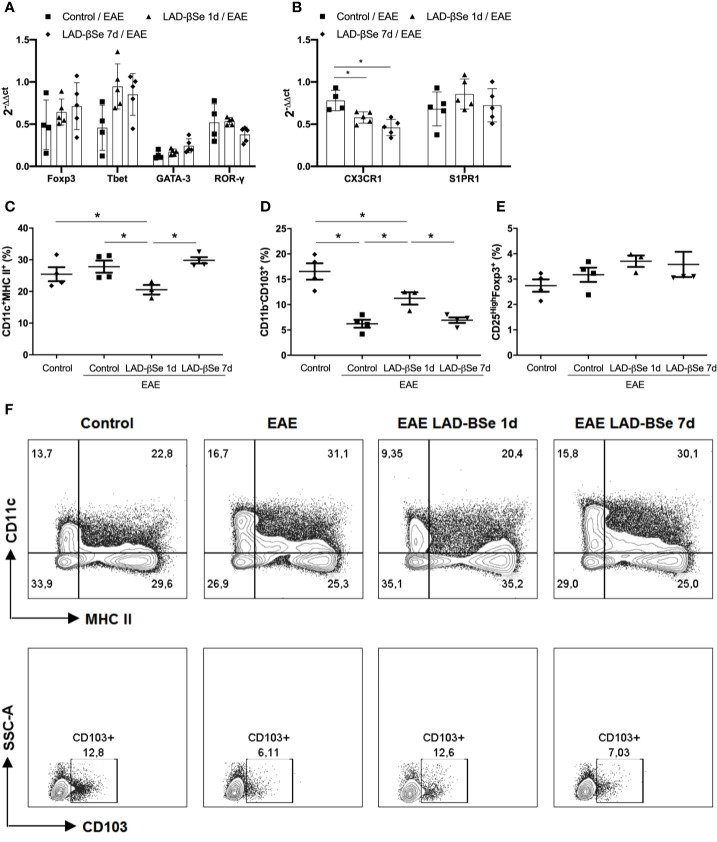
Alterations found in lymph nodes from chronic experimental autoimmune encephalomyelitis (EAE) mice treated with beta-selenium-lactic acid derivative (LAD-βSe). Expression of mRNA transcripts for Foxp3, Tbet, GATA-3 and RORγt **(A)** CX3CR1, and S1PR1 **(B)** in the inguinal lymph nodes were analyzed by real time RT-PCR during EAE acute phase (day 18). The levels of mRNA expression were represented by the values of 2^-ΔΔct^. The values obtained from the analysis of inguinal lymph nodes of healthy mice were used as calibrators of the reaction. The percentage of activated DCs (CD11c^+^ MHCII^+^) **(C)**, tolerogenic DCs (CD11b^-^CD103^+^) **(D)** and Foxp3^+^ Tregs **(E)** in mesenteric lymph nodes were analyzed by flow cytometry. Contour plots representing activated and tolerogenic DCs of a representative animal from each experimental group **(F)**. Results represent the mean ± SEM of three–five animals per group and are representative of 1 experiment. *p < 0.05.

Quantification of mRNA for Foxp3, Tbet, GATA-3, and RORγ in the inguinal lymph nodes showed no significant differences among the experimental groups (**Figure 3A**). Preventive therapy 1 day or 7 days with LAD-βSe significantly decreased the mRNA for CX3CR1 but did not alter the S1PR1 expression (**Figure 3B**).

More pronounced changes were detected in mesenteric lymph nodes. Preventive therapy 1 day reduced the percentage of activated DCs (**Figure 3C**) and augmented the proportion of tolerogenic DCs (**Figure 3D**). The percentage of CD25^High+^Foxp3^+^ cells was not affected by treatment (**Figure 3E**). Contour plots representing the activated (CD11c^+^MHCII^+^) and tolerogenic (CD11b^-^CD103^+^) DCs of one representative animal from each 4 experimental groups are presented in **Figure 3F**. The gateway strategy used to characterize activated (CD11c^+^MHCII^+^) and tolerogenic (CD11b^-^CD103^+^) DCs, and Foxp3^+^ Treg cells are depicted at Supplementary Files.

### LAD-βSe Reaches the CNS and Downregulates Local Inflammation

**Figure 4:**
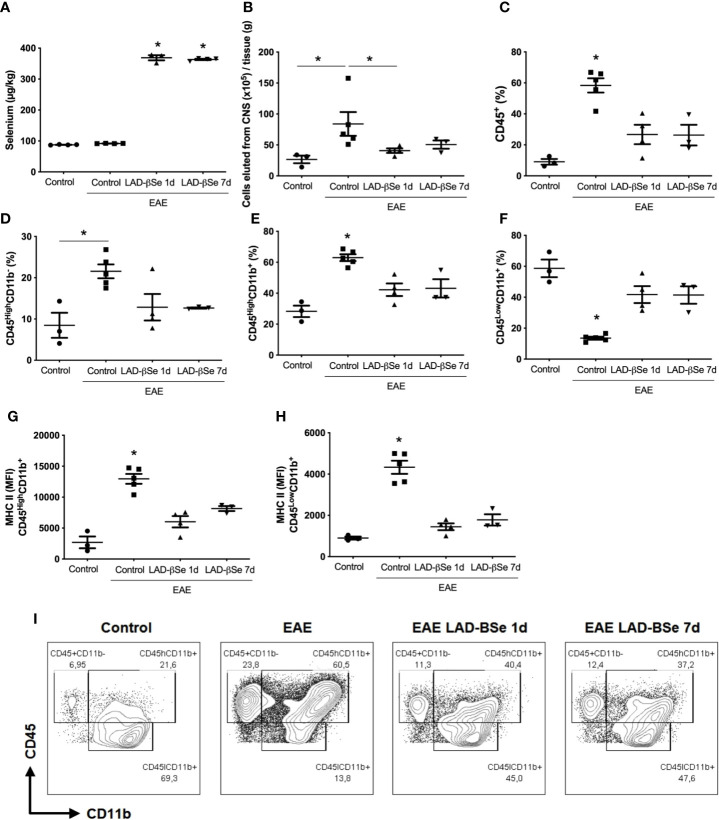
Therapeutic control of chronic experimental encephalomyelitis by administration of beta-selenium-lactic acid derivative (LAD-βSe) is associated with lower infiltration of macrophages and lower expression of MHC in macrophages and microglia during experimental autoimmune encephalomyelitis (EAE) acute phase (day 18). Se concentration in the central nervous system (CNS) was determined by atomic absorption spectrometry **(A)**. Number of cells eluted from the CNS **(B)**, percentage of infiltrating leukocytes plus microglia (CD45^+^) **(C)**, lymphocytes (CD45^High^CD11b^-^) **(D)**, macrophages/activated microglia (CD45^High^CD11b^+^) **(E)**, and resting microglial cells (CD45^Low^CD11b^+^) **(F)**. Mean fluorescence intensity (MFI) of MHCII in macrophages/activated microglia **(G)** and resting microglia **(H)** eluted from the CNS were analyzed by flow cytometry. Contour plots representing leukocytes in the CNS of a representative animal from each experimental group **(I)**. Results represent the median ± SEM of three–five animals per group in each experiment and are representative of two independent experiments. *p < 0.05.

To ascertain whether LAD-βSe formulation was reaching the CNS, Se concentration was evaluated in homogenates from the whole CNS (brain plus spinal cord). As can be observed at **Figure 4A**, Se levels attained high levels in treated mice, that is, about four times higher than the levels found in normal animals. The increase in Se levels was similar in the two therapeutic protocols and, in both, there was downmodulation of infammatory parameters. The number of cells eluted from the CNS at the end of therapy was lower in treated mice; this alteration was even more evident in the early than in the delayed therapy (**Figure 4B**).

The cytometric analysis done with cells eluted from the CNS at the end of therapy pointed to alterations in their proportion and activation status. LAD-βSe administration significantly reduced the percentage of infiltrating leukocytes (**Figure 4C**), and slightly reduced the lymphocyte population (**Figure 4D**). The proportion of infiltrating macrophages/activated microglia was also significantly reduced by both treatments (**Figure 4E**).

**Figure 5:**
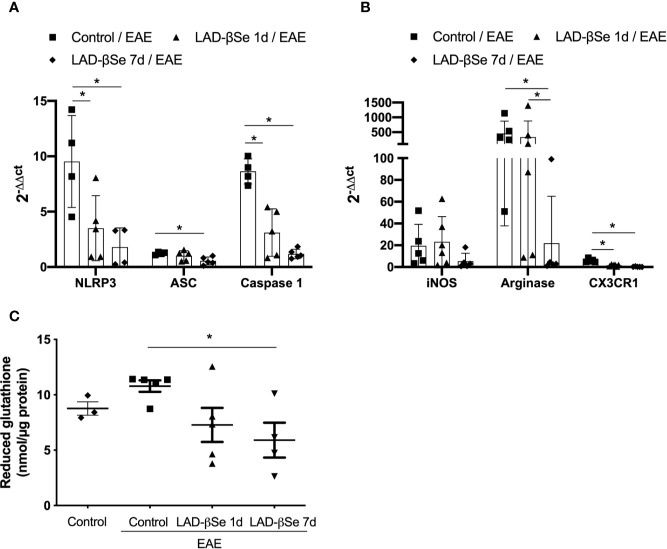
Therapeutic control of chronic experimental encephalomyelitis by administration of beta-selenium-lactic acid derivative (LAD-βSe) controls inflammatory parameters at the lumbar spinal cord. Expression of mRNA transcripts for NOD-like receptor pyrin/domain-containing-3 (NLRP3), caspase-1 and, ASC **(A)**, iNOS, Arginase-1, and CX3CR1 **(B)**. The levels of mRNA expression were determined by real time RT-PCR and the results are represented by the values of 2^-ΔΔct^. The values obtained from the analysis of lumbar spinal cord of healthy mice were used as reaction calibrators. Levels of reduced glutathione **(C)** were determined by biochemical assays. Results represent the median ± SEM of 4-6 animals per group in each experiment and are representative of two independent experiments. *p < 0.05.

The proportion of resting microglia (CD45^Low^ CD11b^+^) (**Figure 4F**) was higher in treated groups. In addition, the treatments significantly decreased the level of MHC expression in infiltrating macrophages/activated microglia (**Figure 4G**) and in resting microglia (**Figure 4H**). Contour plots representing the infiltrating leukocytes of one representative animal from each 4 experimental groups are presented in **Figure 4I**. This downmodulation also included the inflammasome platform. As shown in **Figure 5A**, there was a significant decrease in the mRNA expression of NLRP3, caspase-1 and ASC. iNOS and arginase-1 transcripts were downmodulated in mice treated from the seventh day, but not when the treatment started 24 h after disease induction, as showed in **Figure 5B**. Expression of CX3CR1 mRNA was also significantly reduced by both therapies (**Figure 5B**). Concerning the antioxidant enzymes superoxide dismutase and catalase no significant differences were observed (data not shown). Reduced glutathione was slightly and significantly decreased by early and late therapies, respectively (**Figure 5C**).

### LAD-βSe Downmodulates Neuroinflammation Triggered by LPS

**Figure 6:**
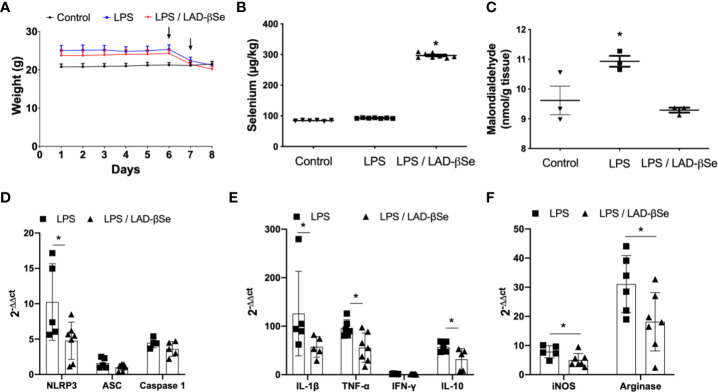
Beta-selenium-lactic acid derivative (LAD-βSe) therapy downmodulates neuroinflammation triggered by LPS. Daily body weight **(A)**, Se concentration in the central nervous system (CNS) determined by atomic absorption spectrometry **(B)** and malondialdehyde levels in the CNS detected by biochemistry analysis **(C)**. Expression of mRNA transcripts for NOD-like receptor pyrin/domain-containing-3 (NLRP3), ASC, and Caspase-1 **(D)**, IL-1β, TNF-α, IFN-γ and IL-10 **(E)**, iNOS and arginase-1 **(F)** in the lumbar spinal cord were analyzed by real time RT-PCR. The levels of mRNA expression were represented by the values of 2^-ΔΔct^. The values obtained from the analysis of lumbar spinal cord of healthy mice were used as reaction calibrators. Results represent the median ± SEM of three–seven animals per group in each experiment and are representative of three independent experiments. *p < 0.05.

To investigate the possibility that LAD-βSe was acting through a direct effect on the CNS, we tested its immunomodulatory activity in a model of neuroinflammation triggered by LPS. **Figure 6A** illustrates the effect of LAD-βSe therapy on body weight loss in mice that were injected with LPS; the two LPS inoculation moments are also indicated by arrows. As can be observed, weight loss was not avoided by the selenized compound. The presence of high Se levels in the CNS of treated mice is illustrated in **Figure 6B**. Many of the local parameters indicative of inflammation were significantly downmodulated by LAD-βSe administration. This was the case of malondialdehyde (**Figure 6C**), NLRP3 (**Figure 6D**), IL-1β (**Figure 6E**), TNF-α (**Figure 6E**), iNOS (**Figure 6F**), arginase 1 (**Figure 6F**), and IL-10 (**Figure 6E**). Other parameters as ASC (**Figure 6D**), caspase-1 (**Figure 6D**), and IFN-γ (**Figure 6E**) displayed just a tendency toward downmodulation.

### Therapeutic Administration of LAD-βSe Controls Relapse in SJL/J Mice

**Figure 7:**
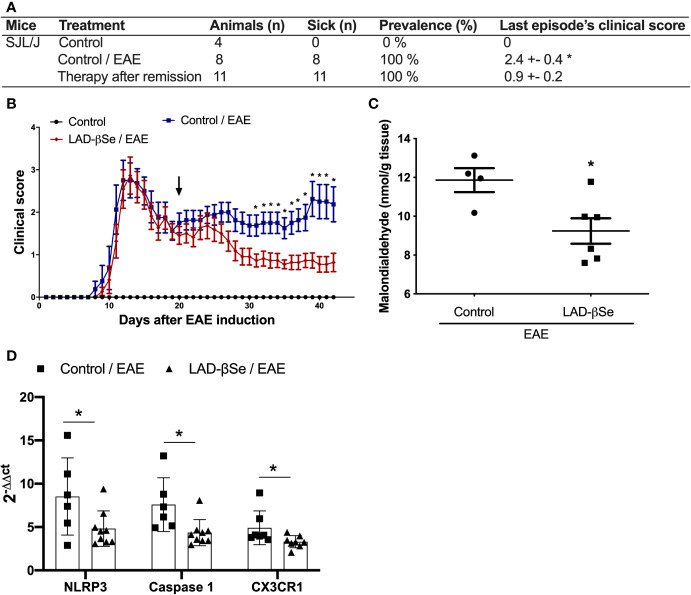
Beta-selenium-lactic acid derivative (LAD-βSe) is also able to control disease reactivation in a relapsing-remitting mice model. Disease prevalence **(A)**, daily clinical score **(B)** and levels of malondialdehyde in the central nervous system (CNS) **(C)**. Expression of the NOD-like receptor pyrin/domain-containing-3 (NLRP3), caspase-1, and CX3CR1 **(D)** mRNA transcripts in the CNS was analyzed by real time PCR 42 days after disease induction. The levels of mRNA expression were represented by the values of 2^-ΔΔct^. The values obtained from the analysis of lumbar spinal cord of healthy mice were used as reaction calibrators. Results represent mean ± SEM of four-nine animals per group in each experiment and are representative of two independent experiments. *p < 0.05.

Differently from the procedure adopted for C57BL/6 mice, the treatment of SJL/J mice started at the 20th day after EAE induction, that is, at the beginning of the first remission. These mice were also daily treated, and therapy was extended until day 42 after disease induction. As showed in **Figure 7A**, the last paralysis episode in the treated group was 2.7 times less intense than in the control one. This significant reduction in the clinical score was already been detected after 10 days of therapy and these reduced scores were maintained throughout the entire treatment (**Figure 7B**). CNS analysis performed after treatment, revealed significant reduction in malondialdehyde levels (**Figure 7C**) and in mRNA expression for NLRP3, caspase-1 and CX3CR1 (**Figure 7D**).

## Discussion

Selenium (Se) is considered a vital microelement being necessary for a plethora of biological pathways like immune system, fertility, thyroid and cardiovascular function (33). Most of its activities are preceded by its incorporation into selenoproteins and, by virtue of its similarity with sulfur, it can replace this chemical element in methionine, cysteine, and cystine and form selenium-containing amino acids. Its effects over the immune system are complex and include participation in the activation, proliferation and differentiation of innate and specific immune cells and also regulation of excessive immune response and chronic inflammation (29). In this context, we tested the potential of an organic Se source constituted by a LAD-βSe, to control the inflammation that compromises functioning of the CNS in two murine models of MS.

Initial experiments were done seeking for a proof of concept that this product under development was able to restrict EAE development. To this end, C57BL/6 mice were continuously supplemented with LAD-βSe for 32 days, beginning 14 days before and ending 18 days after EAE induction, to achieve a condition of supra physiological Se levels. This procedure was highly effective being able to significantly decrease disease severity. CNS analysis indicated clear local effects characterized by less inflammation and less activation of microglia and infiltrating macrophages. Although the results were promising, some evidences as foul breath odor in the animal’s cages suggested toxic effects associated with this prolonged protocol. LAD-βSe was then administered from the 1st or from the 7th day on after EAE induction till the acute disease phase. Both procedures were able to control disease severity without triggering any relevant changes in renal and liver functions. Interestingly, from the point of view of possible transposition to humans, the average maximum clinical score in these two groups was even lower than the observed in animals that underwent the prophylactic-therapeutic schedule.

Se supplementation is already being considered a therapeutic alternative to control inflammatory pathologies as systemic inflammatory response syndrome, asthma, type-2 diabetes, cystic fibrosis and Hashimoto’s thyroiditis (29). Nonetheless, tests with an experimental model of MS, as is the case of EAE, have not being properly carried out. Only one publication showed that diphenyl disselenide, a synthetic organoselenium compound administered by oral route, was able to reduce EAE development (45).

As with the prophylactic-therapeutic scheme, the therapeutic ones also reduced the amount and the activation status of cells directly involved in antigen presentation and neurodegeneration as infiltrating macrophages and microglia. The pivotal role of these cells to both MS and EAE immunopathogenesis is already very well established (46). We then quantified the expression of pro-inflammatory parameters usually associated with the status of infiltrating macrophages and local microglia activation, considering that Se derivatives are able to attenuate the inflammatory responses of these cells (47, 48). The observed results reinforced the local suppression of inflammation by LAD-βSe, including decreased activation of the inflammasome system and reduced expression of mRNA for iNOS and CX3CR1. The critical contribution of the inflammasome in neurodegenerative diseases is supported by many evidences (49). Pharmacological inhibition of inflammasome components is able to control EAE development and therefore is under consideration to treat MS patients (50, 51). The drop in iNOS levels, verified in treated mice, is supported by many reports that found similar downregulation after therapy (52, 53). The impaired expression of CX3CR1 is worth mentioning because this receptor is exquisitely involved in antagonistic functions during EAE/MS development. The hallmark of those two pathologies is the heavy leukocyte infiltration into the CNS (54) and the main role of this receptor, during the initial disease phase, has been attributed to its ability of recruiting leukocytes to the CNS (55). The relevance of this function is illustrated by the demonstration that pharmacological inhibitors of CX3CR1 attenuated disease severity determining reduction in paralysis score, number of relapses and CNS pathology in Dark Agouti rats (56). Otherwise, fractalkine receptor (CX3CR1) deficient mice developed a worse disease that was attributed to decreased migration of NK cells to the CNS (57). NK cells local mechanism of protection has been attributed to their toxicity against encephalitogenic T cells (58). More recently, it was described that CX3CR1 expression, mainly at the microglia, was crucial for both, myelin debris removal and ensuing remyelination (58).

The marked immunomodulation locally observed suggested that LAD-βSe was reaching and acting, at least partially, in the CNS. Effectively, high Se levels, almost 4 times more than the levels found in EAE mice, were detected in the CNS of treated mice. Inspired by the knowledge that Se can upregulate CD4(+) CD25(+) regulatory T cells in other inflammatory diseases (59, 60) and that RORc transcript positive T cells play a pivotal encephalitogenic role in MS and EAE (61, 62), we evaluated the mRNA expression for their signature transcription factors. This was done with samples from the lumbar spinal cord and could help to clarify the possible protective mechanism of LAD-βSe. However, this possibility has not been confirmed (not shown). The high Se levels found in the CNS could, alternatively, be responsible for a local antioxidant therapeutic effect considering that the degree of MS severity has been directly related to the intensity of the oxidative stress (63). Unexpectedly, the most affected parameter was reduced glutathione that was significantly decreased when therapy was delayed to the 7th day after EAE induction. This finding was distinct from the results described in the literature (64) that found higher GSH levels in the brain of EAE rats treated with S-allyl cysteine. It is important to emphasize, however, that Se-containing amino acids, including selenocysteine, can afford direct antioxidant effect (28). To further reinforce the possibility that LAD-βSe was acting locally to control EAE evolution, its effect was tested in a model of neurodegeneration triggered by LPS. Different protocols indicate that peripheral LPS injections in mice trigger neuroinflammation (65, 66). It is believed that LPS effect is mainly due to local activation of microglial cells (67, 68). In this scenario, our results showed that LAD-βSe was able to reach the CNS in this model and, in a great extension, to reduce local inflammatory parameters. Similarly to our findings, reduction in NLRP3, iNOS and TNF-α mRNA expression have been used to ascertain the effect of anti-inflammatory drugs (69, 70).

Even though the peripheral immunological alterations have been discrete, we do not discard the possibility that they contributed to protection by additional mechanisms. As disease was induced by subcutaneous immunization at the lower back, accumulation of effector cells was expected to occur at the inguinal lymph node; they were therefore used to estimate the amount of T cell subsets by assessing their respective signature transcription factors. No significant alterations were, however, detected at the expression of mRNA coding for Foxp3, Tbet, RORc and GATA-3. As LAD-βSe was administered by oral route, the mesenteric lymph nodes were chosen to check for the presence of Tregs and DCs. Although literature data shows that Se is indeed able to increase the number of CD4^+^CD25^+^ regulatory T cells by up-regulating the expression of Foxp3 mRNA in mice (59), there was not a significant increase in the levels of Foxp3 mRNA in the mesenteric lymph nodes. Modulation regarding DCs was detected after the long-term LAD-βSe administration procedure and involved DCs with distinct phenotypes. The clear decrease in activated DCs (CD11b^+^MHCII^+^) associated with an increase in tolerogenic DCs (CD11b^-^CD103^+^) and the known differential outcome triggered by these two APCs populations (effector and regulatory T cells differentiation, respectively) suggest that LAD-βSe is possibly interfering with this stage of the immune response. Interesting, we also do not rule out the possibility that tolerogenic DCs are migrating from the periphery to the CNS where they could shut down lymphocyte proliferation and activation. The phenomenon of migration of this cell type and its potential therapeutic application are under investigation (71, 72).

Among MS murine models, the SJL/J strain immunized with PLP is seen as a model that mimics the relapsing-remitting form of the disease which predominates in human beings and is characterized by a series of flare-ups followed by periods of remission (73). This model was used to assess whether the treatment could restrain disease development after the first disease episode. The results clearly showed that LAD-βSe was also able to significantly reduce the severity of flare-ups.

Our findings are consistent with the pivotal role of Se in neurological health and its relevance in the context of autoimmune diseases. In murine models, for example, the genetic inactivation of selenoprotein P or its neuronal receptor, determines low Se levels in the brain, which leads to spontaneous neurological deficits and neurodegeneration (74). According to a systematic review by Sahebari et al., 2019 (75), reduced serum Se levels were detected in patients with autoimmune diseases. Notably, an adequate dietary Se intake has contributed to the management of complications in autoimmune disorders and has improved patient survival. According to this review, Se effect may be mainly mediated by its anti-inflammatory potential and this micronutrient deserves, therefore, to be considered as a possible nutritional intervention in autoimmune diseases.

Although we consider that the above results could contribute to MS control, we are aware of the existence of limitations that need to be taken into consideration. Unfortunately, we were not able, due to the reduced grant, to perform additional experiments with a pure prophylactic or a pure therapeutic regimen. This is one of the clear limitations of this investigation because this approach could help to define the most appropriate time to administer LAD-βSe to MS patients in the future. As can be realized by the results discussed above, we were also not able to provide a robust data concerning the alterations triggered by LAD-βSe on the cellular lymphoid compartment and the cellular origin of the cytokines locally produced. This limitation deserves to be addressed in future studies. This clarification will allow to confirm if this compound is acting, at least partially, by modulating the plethora of cell subsets that reach or that are being expanded inside the CNS itself. Lastly, and as a consequence of the short mice supply, some healthy control groups were constituted by only 3 animals. This situation did not interfere in the comparison between EAE and EAE-treated experimental groups. However, this did not allow us to know, in some situations, whether the treatment determined or not normalization of the analyzed parameter because this could lead to false positive results when treated and normal healthy control groups were statistically compared.

Altogether our findings indicate that LAD-βSe decreases EAE severity, possibly by modulating the local immune response at the CNS. Results from both models suggest that efficacy does not depend upon a precocious and prolonged LAD-βSe administration. In addition, despite the elevated Se levels found at the CNS, it does not seem to trigger relevant side effects. Future and more detailed preclinical studies will help to predict if LAD-βSe is adequate as an add-on therapy for MS patients.

## Data Availability Statement

The raw data supporting the conclusions of this article will be made available by the authors, without undue reservation.

## Ethics Statement

The animal study was reviewed and approved by Local Ethics Committee on Use of Animals (CEUA) from São Paulo State University (UNESP), Institute of Biosciences, Botucatu, São Paulo, Brazil.

## Author Contributions

JT, AS, and SZ-P conceived and designed the experiments. JT, TF-S, PB, LO, and SZ-P performed the experiments. TF-S performed the flow cytometric analysis. JT, AS, and SZ-P analyzed the data. EO contributed to neuroinflammation induced by LS. LP and CH-L were responsible for oxidative stress determination. AALS contributed to biochemistry analysis. PP was responsible for the spectrophotometric determination of selenium. MP-B, AAS, and CO synthesized the LAD-βSe. JT, AS, and SZ-P wrote the paper. All authors contributed to the article and approved the submitted version.

## Funding

This work was supported by São Paulo Research Foundation (FAPESP), grant number 2016/23318-8, National Council for Scientific and Technological Development (CNPq), grants numbers 307269/2017-5 and 152920/2018-8, and Biorigin Company. JT thanks Coordination for the Improvement of Higher Education Personnel (CAPES) for master’s degree scholarship. MP-B thanks CNPq for PhD scholarship grant number 141779/2014-4.

## Conflict of Interest

CO was employed by Biorigin Company.

The remaining authors declare that the research was conducted in the absence of any commercial or financial relationships that could be construed as a potential conflict of interest.
