# Supplementary material for: Organic Selenium Reaches the Central Nervous System and Downmodulates Local Inflammation: A Complementary Therapy for Multiple Sclerosis?
Source: Front Immunol. 2020 Oct 30;11:571844. doi: 10.3389/fimmu.2020.571844 (PMC7664308; doi:10.3389/fimmu.2020.571844)

(12) DEMANDE INTERNATIONALE PUBLIÉE EN VERTU DU TRAITÉ DE COOPÉRATION EN MATIÈRE DE BREVETS (PCT)

(19) **Organisation Mondiale de la Propriété Intellectuelle**
Bureau international

(43) **Date de la publication internationale**
15 octobre 2015 (15.10.2015)

WIPO | PCT

(10) **Numéro de publication internationale**
WO 2015/155453 A2

(51) **Classification internationale des brevets** : Non classée

(21) **Numéro de la demande internationale** : PCT/FR2015/050886

(22) **Date de dépôt international** : 7 avril 2015 (07.04.2015)

(25) **Langue de dépôt** : français

(26) **Langue de publication** : français

(30) **Données relatives à la priorité** :
1453136 8 avril 2014 (08.04.2014) FR

(71) **Déposant** : **TETRAHEDRON** [FR/FR]; 14 Avenue de l'Opéra, 75001 Paris (FR).

(72) **Inventeurs** : **YADAN, Jean-Claude**; c/o Tetrahedron, 14 Avenue de l'Opéra, 75001 Paris (FR). **ERDELMEIER, Irène**; c/o Tetrahedron, 14 Avenue de l'Opéra, 75001 Paris (FR). **MOUTET, Marc**; c/o Tetrahedron, 14 Avenue de l'Opéra, 75001 Paris (FR). **LEBEL, Rémi**; c/o Tetrahedron, 14 Avenue de l'Opéra, 75001 Paris (FR).

(74) **Mandataire** : **CHAILLOT, Geneviève**; Cabinet Chaillot, 16/20 Avenue de l'Agent Sarre, B.P. 74, 92703 Colombes Cedex (FR).

(81) **États désignés** *(sauf indication contraire, pour tout titre de protection nationale disponible)* : AE, AG, AL, AM, AO, AT, AU, AZ, BA, BB, BG, BH, BN, BR, BW, BY, BZ, CA, CH, CL, CN, CO, CR, CU, CZ, DE, DK, DM, DO, DZ, EC, EE, EG, ES, FI, GB, GD, GE, GH, GM, GT, HN, HR, HU, ID, IL, IN, IR, IS, JP, KE, KG, KN, KP, KR, KZ, LA, LC, LK, LR, LS, LU, LY, MA, MD, ME, MG, MK, MN, MW, MX, MY, MZ, NA, NG, NI, NO, NZ, OM, PA, PE, PG, PH, PL, PT, QA, RO, RS, RU, RW, SA, SC, SD, SE, SG, SK, SL, SM, ST, SV, SY, TH, TJ, TM, TN, TR, TT, TZ, UA, UG, US, UZ, VC, VN, ZA, ZM, ZW.

(84) **États désignés** *(sauf indication contraire, pour tout titre de protection régionale disponible)* : ARIPO (BW, GH, GM, KE, LR, LS, MW, MZ, NA, RW, SD, SL, ST, SZ, TZ, UG, ZM, ZW), eurasien (AM, AZ, BY, KG, KZ, RU, TJ, TM), européen (AL, AT, BE, BG, CH, CY, CZ, DE, DK, EE, ES, FI, FR, GB, GR, HR, HU, IE, IS, IT, LT, LU, LV, MC, MK, MT, NL, NO, PL, PT, RO, RS, SE, SI, SK,

[Suite sur la page suivante]

(54) **Title** : NOVEL ORGANOSELENIUM COMPOUNDS, METHOD FOR PRODUCING SAME, AND PHARMACEUTICAL USES THEREOF AS ANTITUMOUR AGENTS

(54) **Titre** : NOUVEAUX COMPOSES ORGANO-SELENIES, PROCÉDÉ DE FABRICATION ET APPLICATIONS EN PHARMACIE NOTAMMENT COMME AGENTS ANTI-TUMORAUX

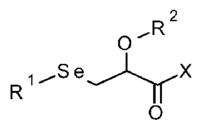

(I)

Figure 2 : Pourcentage de viabilité des cellules DU145 et LS174T en fonction de la concentration en composé 4, après 96 heures de traitement
AA

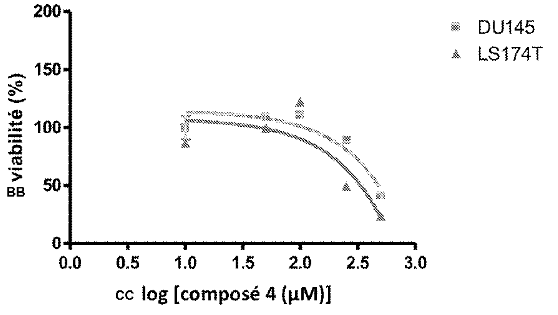

AA Viability percentage of cells DU145 and LS174T versus the concentration of compound 4, after 96 hours of treatment
BB Viability (%)
CC log [compound 4 (μM)]

(57) **Abstract** : The invention relates to a selenium compound. Said selenium compound has formula (I), where R^1 = alkyl; R^2 = H, $R^4C(=0)$, $R^4OC(=0)$, a-aminoacyl, $CH_3SeCH_2CH_2CH(NH_2)C(=0)$, $CH_3SeCH_2CH_2CH(OH)C(=0)$; X = OH, OR^3, NH_2, NR^4R^5, α-amino acid, $CH_3SeCH_2CH_2CH(COOH)NH$-, $CH_3SeCH_2CH_2CH(COOH)0$-; R^3 = alkyl; R^4 = alkyl, aryl; R^5 = H, alkyl, aryl; R^4 and R^5 which can together form a 5- or 6-membered cycloalkyl radical which can comprise a heteroatom; provided that when X = NH-terbutyl, $R^2 \neq C(=0)CH_3$. Said compound can be used as a pharmaceutical substance, in particular as an anitumour ssubstance.

(57) **Abrégé** : L'invention concerne un composé sélénié. Ce composé sélénié répond à la formule (I): dans laquelle R^1= alkyle; R^2 = H, $R^4C(=0)$, $R^4OC(=0)$, a-amino-acyle, $CH_3SeCH_2CH_2CH(NH_2)C(=0)$, $CH_3SeCH_2CH_2CH(OH)C(=0)$; X = OH, OR^3, NH_2, NR^4R^5, α-amino-acide, $CH_3SeCH_2CH_2CH(COOH)NH$-, $CH_3SeCH_2CH_2CH(COOH)0$-; R^3 = alkyle; R^4 = alkyle, aryle; R^5 = H, alkyle, aryle; R^4 et R^5 pouvant former ensemble un radical cyclo-alkyle à 5 ou 6 chaînons pouvant comporter un hétéroatome; étant entendu que lorsque X = NHterbutyle alors $R^2 \neq C(=0)CH_3$. Ce composé est utile comme substance pharmaceutique notamment antitumorale.

WO 2015/155453 A2

SM, TR), OAPI (BF, BJ, CF, CG, CI, CM, GA, GN, GQ, GW, KM, ML, MR, NE, SN, TD, TG).

Déclarations en vertu de la règle 4.17 :

— *relative à la qualité d'inventeur (règle 4.17.iv))*

Publiée :

— *sans rapport de recherche internationale, sera republiée dès réception de ce rapport (règle 48.2.g))*

NOUVEAUX COMPOSES ORGANO-SELENIES, PROCÉDÉ DE FABRICATION ET APPLICATIONS EN PHARMACIE NOTAMMENT COMME AGENTS ANTI-TUMORAUX

La présente invention a pour objet :

- des nouveaux composés organo-séléniés;
- leurs procédés de préparation ;
- leur utilisation en tant que principe actif en pharmacie ;
- ainsi que des compositions pharmaceutiques les renfermant, notamment comme nouveaux agents anti-tumoraux.

Etat de l'Art

Cette invention se rapporte à de nouveaux composés organo-séléniés, leur procédé de préparation, leur utilisation en tant que principe actif pharmaceutique, ainsi que des compositions pharmaceutiques les renfermant, notamment comme nouveaux agents anti-tumoraux. Plus particulièrement, cette invention concerne la préparation de composés organo-séléniés comprenant l'acide 2-hydroxy-3-méthylsélénopropanoïque, ses sels ainsi que les esters et les amides dérivés de l'acide 2-hydroxy-3-méthylsélénopropanoïque, doués d'activité pharmaceutique et notamment d'activité anti-tumorale ou anti-cancer, seuls ou en association avec d'autres agents pharmaceutiques et notamment des agents anti-tumoraux.

Le cancer reste l'une des principales causes de décès dans les pays industrialisés. En dépit des nombreux progrès dans le développement de nouvelles thérapies anticancéreuses, un traitement curatif reste encore un besoin réel pour la plupart des tumeurs solides. Le potentiel anti-tumoral du « sélénium » a été identifié, à la fin des années 60, suite à des études mettant en évidence des taux de mortalité due au cancer plus faibles dans certaines régions dont le sol était riche en sélénium inorganique. Depuis une vingtaine d'années, de nombreux travaux ont mis en évidence, dans le cadre d'expérimentations animales, l'activité anti-cancéreuse du « sélénium » dans un certain nombre d'organes [Papp LV *et al.*, 2007, *Antioxid Redox Signal*, 9:775]. Sur un total de huit essais cliniques d'évaluation de l'effet du « sélénium » sur l'incidence de cancers, sept ont abouti à des résultats positifs [Whanger PD, 2004, *Br. J. Nutr.*, 91(1):11]. Ceci confirme les nombreuses études réalisées chez l'animal.

L'activité anti-tumorale de la méthylsélénocystéine (MeSeCys) a été mise en évidence par Ip et coll. au début des années 2000 [Medina D *et al.*, 2001, *Nutrition and Cancer*, 40(1):12]. Pour être efficace, la MeSeCys doit être « activée » en méthylsélénol par la β-lyase [Ip C. *et al.*, 2002 *Cancer & Metast. Rev.*, 21(3-4):281]. Les cellules tumorales humaines de cancer du sein ou de la prostate présentent un faible niveau d'activité β-lyase, ce qui entraine une faible sensibilité de ces cellules tumorales à des concentrations physiologiquement acceptables de MeSeCys. Par contre des doses journalières de 3mg équivalent Sélénium/kg (eqSe/kg) de MeSeCys administrées pendant 18 à 26 semaines à des souris transgéniques portant un adénome prostatique retardent la progression des lésions tumorales, augmentent l'apoptose et réduisent la prolifération des cellules tumorales [Wang L *et al.,* 2009, *Cancer Prev. Res.*, 2:484].

La MeSeCys induit l'apoptose des cellules de carcinomes par activation de la voie des caspases [Suzuki M. *et al.*, 2010, *Cancer Chemother. Pharmacol.*, 66(3):475]. La MeSeCys inhibe la croissance de cellules mammaires, et induit l'apoptose selon un mécanisme caspase-dépendent et implique la libération du cytochrome C mitochondrial ainsi que la fragmentation de l'ADN nucléosomal [Hu H *et al.*, 2005, *Carcinogenesis,* 26:1374]. Ce même composé inhibe la progression du cancer de la prostate dans un modèle murin et augmente la survie des souris [Wang L *et al.*, 2009, *Cancer Prev. Res.*, 2:484].

La MeSeCys, n'est pas facilement synthétisable et nécessite, de plus, une activation métabolique pour être efficace.

L'acide méthylséléninique (MSA) a été utilisé pour sa capacité à générer du méthylsélénol CH_3SeH après réduction par des réducteurs biologiques tel que le glutathion intracellulaire. Ceci lui confère une activité anti-cancéreuse dont il a été démontré qu'elle est supérieure à celle de la MeSeCys [Ip C *et al.*, 2000, *Cancer Res.*, 60:2882] ainsi qu'à celle de la sélénométhionine [Li GX *et al.*, 2008, *Carcinogenesis*, 29:1005]. Le MSA est capable de moduler plusieurs bio-marqueurs spécifiques ce qui aboutit à une diminution de la prolifération cellulaire et une activation de l'apoptose [Dong Y. *et al.*, 2002, *Anticancer Res.*, 22:27]. Il induit un stress au niveau du réticulum endoplasmique par oxydation des thiols de protéines qui conduit à un repliement anormal de ces dernières. Si le système de réparation est « débordé », alors la cellule s'engage dans un processus d'apoptose [Wu Y *et al.*, 2005, *Cancer Res.*, 65(19):9073]. Par ailleurs, le MSA diminue, de façon dose-dépendante, le niveau du facteur inductible d'hypoxie HIF-1α dans des cellules d'adénome prostatique hormono-indépendantes [Sinha R *et al.*, 2008, *Cancer Prev. Res.*, 1, 7, Suppl., abstract n°B117]. Le MSA réduit les métastases spontanées de cellules cancéreuses de poumon chez la souris [Yan L *et al.*, 2011, *Int. J. Cancer,*

131:1260]. Le MSA, bien que très actif, est difficilement utilisable en tant que principe actif de médicament pour des raisons évidentes liées à sa formulation et à sa stabilité.

Enfin, d'autres composés organo-séléniés ont été décrits comme ayant des propriétés anti-cancéreuses tels que le S,S'-1,4-phenylènebis(1,2-éthanediyl)-isoséléno-urée [SubbaRao VM *et al.*, 2008, *Mol. Cancer Ther.*, 7(5):1297], le 1,4-phenylènebis(méthylène)-sélénocyanate [Chen KM, 2007, *Cancer Res.*, 67(21):10475] ainsi qu'une série d'isosélénocyanates [Sharma A, 2009, *Clin. Cancer Res.*, DOI 10.1158/1078-0432]. Pour les mêmes raisons que le MSA, ces composés sont très difficilement utilisables comme médicaments.

Le document SHAABAN, dans European Journal of Medicinal Chemistry, 58,(2012), P192-205, décrit divers composés contenant des chalcogènes actifs redox et des quinones comme agents anti-cancer potentiels. A la page 195, SHAABAN décrit des composés dans lesquels R1 pourrait comprendre un noyau aromatique (pouvant être aryle) et R2= C(=O)R4 et R4O(C=O) qui ont une similitude avec R4 = aryle des composés de la formule (I) de la présente invention décrite ci-après.

Cependant, dans les composés de formule (I) de la présente invention décrite ci-après, un noyau aromatique n'est prévu qu'en second, c'est-à-dire sous forme alkyl-aryle avec alkyle attaché au Sélénium.

De ce fait, les composés de formule (I) sont nouveaux par rapport aux composés 4 à 18, page 195 de SHAABAN,.

Par ailleurs, pour les composés 4, 5, 7, 10 et 11 de SHAABAN qui ont un groupe NH-tertbutyle, correspondant à X de la formule (I) de la présente invention, les composés de formule (I) de la présente invention sont en outre différents car dans ce cas R2 est ≠ de C(=O) CH3 et aussi R2 est seulement alkyle ou aryle.

Egalement, les composés de formule (I) n'ont pas de groupe redox type quinone.

Par ailleurs, le document ABBAS , publié dans www.rsc.org/chemcomm, Royal Society of Chemistry, 2006, P541-543, décrit divers composés n'ayant pas d'activité anti-cancer parmi lesquels un composé 7t à la page 542 qui a une substitution méthyle sur Se correspondant à R1 de la formule (I) de la présente invention décrite ci-après.

Cependant, le substituant en haut de la formule de 7t correspond au substituant X de la formule (I) de la présente invention décrite ci-après mais la définition donnée prévoit pour X =**alpha**-aminoacide un radical de type -NHCH(Y)COOH (**alpha**-aminoacide) qui exclut le radical -NH-(CH2)5-COOBn (**epsilon**-aminoacide) du composé 7t de ABBAS.

En outre le substituant en bas de la formule de 7t= –O- C(=O)-CH2-NH-C(=O)-O-tertButyl correspond au substituant –O-R2 lorsque R2= R4(C=O) mais R4

= alkyle ou aryle dans le cadre de la présente invention.
Et, la définition interprétée correctement de « alkyle » de la présente invention décrite ci-après prévoit une substitution notamment par un groupe amine et/ou acide ou ester mais pas une fonction NH-C(=O)-O-R qui permettrait de correspondre à 7t décrit dans ABBAS.

Ainsi, la définition de alkyle dans la formule (I) de la présente invention ne couvre pas le composé 7t de ABBAS.

Pour les raisons exposées ci-dessus, les produits de l'art antérieur ayant des propriétés anti-cancéreuses ne sont pas satisfaisants en tant qu'agents anti-tumoraux.

BUTS DE L'INVENTION

L'un des buts de la présente invention est de concevoir de nouveaux composés organo-séléniés facilement synthétisables et formulables.
Un autre des buts de la présente invention est de préparer ces nouveaux composés organo-séléniés pouvant être synthétisés en grande quantité par des procédés utilisables à l'échelle industrielle.

Un autre des buts de la présente invention est de préparer de nouveaux composés organo-séléniés facilement formulables en tant qu'agents pharmaceutiquement actifs et notamment en tant qu'agents anti-tumoraux ou anti-cancer.

Un autre des buts de la présente invention est de préparer de nouvelles compositions pharmaceutiques contenant au moins l'un de ces nouveaux composés organo-séléniés, seul ou en association avec un autre agent anti-tumoral ou anti-cancer.

Ces buts sont atteints grâce à la présente invention qui repose sur la conception et la préparation de nouveaux composés organo-séléniés et leurs esters et amides ainsi que leurs applications en pharmacie notamment en tant qu'agents anti-tumoraux. Ceci a été exemplifié dans la présente invention.

Description de l'invention

La présente invention a donc pour but :

1) de résoudre le nouveau problème technique consistant en la fourniture de nouveaux composés organo-séléniés et leurs dérivés esters et amides constituant ainsi des principes actifs de compositions pharmaceutiques ;

2) de résoudre ce nouveau problème technique selon une solution qui englobe un procédé de préparation utilisable à l'échelle industrielle de ces nouveaux composés organo-séléniés et de leurs dérivés esters et amides ;

3) de proposer des compositions pharmaceutiques de ces composés organo-séléniés répondant à la formule générale (I) seuls ou en association avec d'autres agents pharmaceutiques, notamment d'autres agents anti-tumoraux.

Les problèmes techniques énoncés ci-dessus sont résolus pour la première fois d'une manière simultanée par la présente invention, de façon très aisée et économique, le procédé de préparation desdits nouveaux composés étant très simple à mettre en œuvre tout en fournissant de bons rendements.

Selon son premier aspect, la présente invention a pour objet de nouveaux agents organo-séléniés répondant à la formule générale (I) suivante :

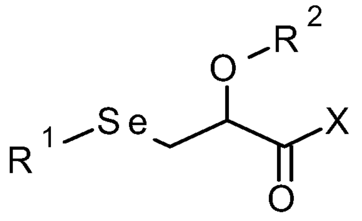

FORMULE GÉNÉRALE (I)

dans laquelle

R[1] = alkyle;

R[2] = H, **R**[4]C(=O), **R**[4]OC(=O), α-amino-acyle, $CH_3SeCH_2CH_2CH(NH_2)C(=O)$, $CH_3SeCH_2CH_2CH(OH)C(=O)$;

X = OH, O**R**[3], NH_2, N**R**[4]**R**[5], α-amino-acide, $CH_3SeCH_2CH_2CH(COOH)NH$- , $CH_3SeCH_2CH_2CH(COOH)O$-;

R[3] = alkyle;

R[4] = alkyle, aryle ;

R[5] = H, alkyle, aryle ;

R[4] et **R**[5] pouvant former ensemble un radical cyclo-alkyle à 5 ou 6 chainons pouvant comporter un hétéroatome;

étant entendu que lorsque **X** = NHterbutyle alors **R**[2] ≠ $C(=O)CH_3$

L'invention englobe tous les stéréoisomères, diastéréoisomères et énantiomères notamment au niveau de l'atome de carbone portant le groupement O**R**[2], ainsi qu'au niveau des radicaux **R**[1] à **R**[5], ainsi que tous oligomères (dimères, trimères, ...) et polymères, linéaires ou ramifiés, acycliques ou cycliques, obtenus entre deux ou plusieurs molécules de dérivés de formule (I) décrits dans l'invention par réaction

d'estérification entre les fonctions alcool et acide carboxylique présentes le cas échéant, pris isolément ou en mélange.

Elle englobe également tous les sels d'acides ou de bases pharmaceutiquement acceptables desdits composés de formule générale (I), ainsi que les sels de sodium, de calcium, de zinc et de magnésium.

Parmi les composés de formule générale (I), l'invention a notamment pour objet :

- ceux caractérisés en ce que $\mathbf{R^1}$ représente un groupe méthyle, éthyle ou allyle;
- ceux caractérisés en ce que $\mathbf{R^2}$ est choisi dans le groupe constitué par H, α-amino-acyles, $CH_3SeCH_2CH_2CH(OH)C(=O)$, $R_4(C=O)$, $R_4O(C=O)$;
- ceux caractérisés en ce que **X** est choisi dans le groupe OH, α-amino-acide, $CH_3SeCH_2CH_2CH(COOH)NH$-, $CH_3SeCH_2CH_2CH(COOH)O$- ;
- ceux caractérisés en ce que R^1 représente un groupe méthyle, éthyle, allyle ; R^2 représente R4(C=O), R4O(C=O) et X représente OH ou OR3.
- ceux préparés dans la partie expérimentale, notamment les composés 4, 10, 38, 46 et 48.

Parmi les acides pharmaceutiquement acceptables, on peut citer, à titre non limitatif, les acides minéraux tels que les acides chlorhydrique, bromhydrique, iodhydrique, sulfurique, tartrique, phosphorique ou avec les acides organiques tels que les acides formique, acétique, trifluoroacétique, propionique, benzoïque, maléique, fumarique, succinique, citrique, oxalique, glyoxylique, aspartique, alcanesulfoniques tels que les acides méthane-sulfonique, trifluorométhane-sulfonique, éthane-sulfonique, aryl-sulfoniques tels que les acides benzène- et paratoluène-sulfonique.

Parmi les bases pharmaceutiquement acceptables, on peut citer, à titre non limitatif, les bases minérales telles que les hydroxydes de sodium, de lithium, de calcium, de potassium, de magnésium, d'ammonium ou de zinc, les carbonates de métaux alcalins ou alcalino-terreux tels que les carbonates et bicarbonates de sodium, de lithium, de calcium, de potassium, de magnésium, d'ammonium ou de zinc ou des bases organiques comme la méthylamine, la propylamine, la triméthylamine, la diéthylamine, la triéthylamine, la N,N-diméthyléthanolamine, le tris(hydroxy-méthyl)aminométhane, l'éthanolamine, la pyridine, la picoline, la dicyclohexyl-amine, la morpholine, la proceïne, la lysine, l'arginine, l'histidine, la N-méthylglucamine ou encore les sels de phosphonium tels que les sels d'alkyl-phosphonium, les sels d'aryl-phosphonium, les sels d'alkyl-aryl-phosphonium, les alkènyl-aryl-phosphonium ou les sels d'ammonium quaternaires tels que les sels de tétra-n-butyl-ammonium.

Dans la formule (I) ci-dessus :

- par alkyle, il est entendu un radical carboné saturé de 1 à 26 atomes de carbone linéaire, ramifié ou cyclique, pouvant être substitué par un ou plusieurs atomes de fluor, ou pouvant être substitué par une ou plusieurs doubles liaisons carbone-carbone, ou pouvant être substitué par au moins un groupe OH ou par au moins un groupe amine, ou par au moins un groupe COOH ou $COOR^6$, R^6 étant un groupe également choisi parmi un radical carboné saturé ou non saturé de 1 à 26 atomes de carbone linéaire, ramifié ou cyclique non substitué tel que par exemple méthyle, éthyle, isopropyle, ou pouvant être substitué par un ou plusieurs atomes de fluor tel que par exemple trifluorométhyle, ou pouvant être substitué par une groupe phényle tel que par exemple benzyle, ou pouvant être substitué par un radical allyle tel que par exemple linoléyle, linolényle, palmitoyle, allyle.

- par aryle, il est entendu un radical phényle pouvant être substitué par un ou plusieurs atomes de fluor, ou un radical pyridinyle pouvant être substitué par un ou plusieurs atomes de fluor, ou un radical imidazole.

- par α-aminoacyle, il est entendu un radical de type –C(=O)CH(Y)NH2, Y correspondant à la chaine latérale de l'un des aminoacides protéogèniques c'est-à-dire des aminoacides constituant les protéines.

- par α-aminoacide, il est entendu un radical de type –NHCH(Y)COOH, Y correspondant à la chaine latérale de l'un des aminoacides protéogèniques c'est-à-dire des aminoacides constituant les protéines.

- par oligomère, il est entendu tout composé constitué par l'enchaînement de 2 à 15 monomères tels que décrits dans l'invention reliés entre eux par l'intermédiaire d'une liaison de type ester.

- par polymère, il est entendu tout composé constitué par l'enchaînement de plus de 15 monomères tels que décrits dans l'invention reliés entre eux par l'intermédiaire d'une liaison de type ester.

- par dimère cyclique, il est entendu le composé constitué par la formule suivante :

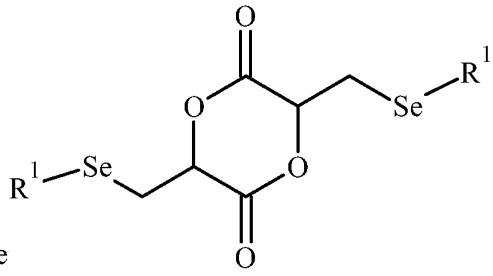

qui relie entre [illegible]ule générale (I) tels que décrits dans l'inve[illegible]ons ester entre les 2 groupements hydroxy-acides.

Dans un autre mode de réalisation, le composé de formule (I) est choisi parmi les composés cités en exemples.

Ainsi, le composé sélénié de formule (I) peut être choisi parmi le groupe consistant de :

l'ester méthylique de l'acide 2-hydroxy-3-(méthylséléno)propanoïque ;
l'ester méthylique de l'acide (*R*)-2-hydroxy-3-(méthylséléno)propanoïque ;
l'ester méthylique de l'acide (*S*)-2-hydroxy-3-(méthylséléno)propanoïque ;
l'ester éthylique de l'acide 2-hydroxy-3-(méthylséléno)propanoïque ;
l'ester *tert*-butylique de l'acide 2-hydroxy-3-(méthylséléno)propanoïque ;
l'ester benzylique de l'acide (*S*)-(2-hydroxy-3-(méthylséléno)propanoïque ;
l'ester benzylique de l'acide (*S*)-(2-hydroxy-3-(méthylséléno)propanoïque ;
l'ester méthylique de l'acide 3-(éthylséléno)-2-hydroxypropanoïque ;
l'ester méthylique de l'acide 2-hydroxy-3-(isobutylséléno)propanoïque ;
l'ester isopropyle de l'acide 2-hydroxy-3-(méthylséléno)propanoïque ;
l'acide 2-hydroxy-3-(méthylséléno)propanoïque ;
l'acide (*R*)-2-hydroxy-3-(méthylséléno)propanoïque ;
l'acide (S)-2-hydroxy-3-(méthylséléno)propanoïque ;
l'acide 3-éthylséléno-2-hydroxypropanoïque ;
l'acide 2-hydroxy-3-(isobutylséléno)propanoïque ;
le sel dicyclohexylammonium 2-hydroxy-3-(méthylséléno)propanoate ;
le sel de sodium 2-hydroxy-3-(méthylséléno)propanoate ;
le sel de magnesium bis(2-hydroxy-3-(méthylséléno)propanoate ;
le sel du zinc bis(2-hydroxy-3-(méthylséléno)propanoate ;
le sel du calcium bis(2-hydroxy-3-(méthylséléno)propanoate ;
le 2-hydroxy-3-(méthylséléno)propanamide ;
le N-cyclopropyl-2-hydroxy-3-(méthylséléno)propanamide ;
le N-[2-(diméthylamino)éthyl]-2-hydroxy-3-(méthylséléno)propanamide ;
la 2-hydroxy-3-(méthylséléno)-1-(pyrrolidin-1-yl)propan-1-one ;
la 2-hydroxy-3-(méthylséléno)-1-(pipéridin-1-yl)propan-1-one ;
la 2-hydroxy-3-(méthylséléno)-1-(morpholin-4-yl)propan-1-one ;
le N,N-diéthyl-2-hydroxy-3-(méthylséléno)-propanamide ;
le 2-hydroxy-N-(2-hydroxyéthyl)-3-(méthylséléno)-propanamide ;
l'ester *tert*-butylique du [(2*RS*)-2-hydroxy-3-méthylsélénopropanoyl]-(*S*)-alanine ;
la [(2*RS*)-2-hydroxy-3-méthylsélénopropanoyl]-(*S*)-alanine ;
l'ester méthylique du [(2*RS*)-2-hydroxy-3-méthylsélénopropanoyl]-(*S*)-méthionine ;
la [(2*RS*)-2-hydroxy-3-méthylsélénopropanoyl]-(*S*)-méthionine ;
l'ester méthylique de la [(2*R*)-2-hydroxy-3-méthylsélénopropanoyl]-(*S*)-méthionine ;
la [(2*R*)-2-hydroxy-3-méthylsélénopropanoyl]-(*S*)-méthionine ;
l'ester méthylique de la [(2*S*)-2-hydroxy-3-méthylsélénopropanoyl]-(*S*)-méthionine ;
la [(2*S*)-2-hydroxy-3-méthylsélénopropanoyl]-(*S*)-méthionine ;

l'ester méthylique de la N(α)-[(2*RS*)-2-hydroxy-3-méthylsélénopropanoyl]-N(□)-*tert*-butoxycarbonyl-(*S*)-lysine ;

l'ester méthylique de la N(α)-[(2*RS*)-2-hydroxy-3-méthylsélénopropanoyl]-N(□)-fluorenylméthyloxycarbonyl-(*S*)-lysine ;

l'ester méthylique de la N(α)-[(2*RS*)-2-hydroxy-3-méthylsélénopropanoyl]-N(□)-benzyloxycarbonyl-(*S*)-lysine ;

l'ester méthylique de la [(2*RS*)-2-hydroxy-3-méthylsélénopropanoyl]-(*S*)-sélénométhionine ;

la [(2*RS*)-2-hydroxy-3-méthylsélénopropanoyl]-(*S*)-sélénométhionine ;

l'ester méthylique de la [(2*R*)-2-hydroxy-3-méthylsélénopropanoyl]-(*S*)-sélénométhionine ;

la [(2*R*)-2-hydroxy-3-méthylsélénopropanoyl]-(*S*)-sélénométhionine ;

l'ester méthylique de la [(2*S*)-2-hydroxy-3-méthylsélénopropanoyl]-(*S*)-sélénométhionine ;

la [(2*S*)-2-hydroxy-3-méthylsélénopropanoyl]-(*S*)-sélénométhionine ;

l'acide 2-(acétyloxy)-3-(méthylséléno)propanoïque ;

l'acide 2-(dodécanoyloxy)-3-(méthylséléno)propanoïque ;

l'ester méthylique de l'acide 2-(benzoyloxy)-3-(méthylséléno)propanoïque ;

l'acide 2-(benzoyloxy)-3-(méthylséléno)propanoïque ;

l'ester méthylique de l'acide 3-(méthylséléno)-2-[(3'-pyridine)oxycarbonyl]propanoïque ;

l'ester méthylique de l'acide 2-[(*tert*-butoxycarbonyl)oxy]-3-(méthylséléno)propanoïque ;

l'acide 2-[(*tert*-butoxycarbonyl)oxy]-3-(méthylséléno)propanoïque ;

l'ester méthylique de l'acide (2*RS*)-[N-(*tert*-butoxycarbonyl)-*S*-methionyl]-3-(méthylséléno)propanoïque ;

l'ester méthylique de l'acide 4-méthylséléno-2-(2'-acétyloxy-3'-méthylsélénopropanoyl)butyrique ;

l'acide 4-méthylséléno-2-(2'-acétyloxy-3'-méthylsélénopropanoyl)butyrique ;

l'ester méthylique de l'acide 3-méthylséléno-2-(2'-acétyloxy-4'-méthylsélénobutanoyl)propanoïque ;

l'acide 3-méthylséléno-2-(2'-hydroxy-4'-méthylsélénobutanoyl)-propanoïque ;

l'acide 2-(pentanoyloxy)-3-(méthylséléno)-propanoïque ;

l'acide 2-(nonanoyloxy)-3-(méthylséléno)-propanoïque ;

l'acide 2-(linoleoyloxy)-3-(méthylséléno)-propanoïque ;

l'ester méthylique de l'acide 2-(linoleoyloxy)-3-(méthylséléno)-propanoïque ;

l'acide 2-(pivaloyloxy)-3-(méthylséléno)-propanoïque ;

l'acide 2-(3-chloropropanoyloxy)-3-(méthylséléno)-propanoïque ;

l'acide 2-{(1H-imidazoyl-4-ylcarbonyl)oxy)}-3-(méthylséléno)-propanoïque ;
l'ester méthylique de l'acide 2-(pivaloyloxy)-3-(méthylséléno)-propanoïque ;
ainsi que leurs mélanges en toutes proportions.

Selon un second aspect, l'invention concerne un procédé de préparation des nouveaux composés organo-séléniés et de leurs dérivés esters et amides de formule générale (I), explicité sur la Figure 1, et caractérisé en ce qu'il comprend les étapes suivantes :

1) la réaction d'un ester de l'acide oxirane-2-carboxylique racémique (DL) ou de l'un de ses énantiomères (D ou L) disponibles commercialement (par exemple chez SAF France), avec

a/ soit avec un dérivé dialkyle aluminium alkylsélénolate de formule $Al(R^1)_2SeR^1$, lui-même généré *in situ* à partir du trialkyle aluminium $Al(R^1)_3$ correspondant, disponible commercialement (par exemple chez SAF, France) et du sélénium élémentaire Se° disponible commercialement (par exemple chez SAF, France) (selon Kozikowski AP and Ames A, 1978, *J. Org. Chem.*, 43(13):2735).

b soit un alkylsélénole R^1SeH, lui-même préparé *in situ* à partir d'un sel alcalin d'alkyle sélénolate de formule R^1-Se-M_1 obtenu lui-même par réduction du dialkyle disélénure correspondant disponible commercialement (par exemple chez SAF, France) ou par réaction entre du sélénium métal Se(0) et un sel d'alkyle lithium correspondant disponible commercialement (par exemple chez SAF, France) dans laquelle M_1 représente un atome de métal alcalin), mis en réaction avec du chlorure d'ammonium (en analogie avec l'ouverture des alkyles oxirane carboxylates avec un thiol tel que décrit dans *Org. Letters*, 2004, 6(4), 497) ;

2) le cas échéant une, ou plusieurs, des réactions ou séries de réactions suivantes (voir Figure 1) bien connues de l'homme de l'art:

- hydrolyse de la fonction ester, puis
- acidification du milieu réactionnel pour obtenir les acides correspondants de formule (I) où X = OH; puis
- estérification des acides de formule (I) ou de leurs sels alcalins de formule (Ia) avec un alcool ou un halogénure d'alkyle pour obtenir les esters correspondants de formule générale (I) dans laquelle X = OR^3, avec R^3 tel que défini précédemment;
- amidification des acides de formule (I) avec une amine appropriée de formule R^4R^5NH, ou NH_3 dans laquelle R^5 est tel que défini précédemment, pour obtenir le composé de formule générale (I) dans laquelle X = NH2, NR^4R^5 ou α-aminoacide, $CH_3SeCH_2CH_2CH(COOH)NH$- ;

- estérification, lorsque R^2 = H, de la fonction hydroxyle par un acide aproprié pour obtenir le composé de formule générale (I) dans laquelle OR^2 est différent du groupe OH ;
- salification par un acide ou par une base.

Selon une mise en œuvre particulière du procédé selon l'invention :

le réactif sélénié est :

* soit un dialkyle aluminium alkylsélénolate, et par exemple le diméthyle aluminium méthylsélénolate généré *in situ* à partir de sélénium métal Se° et du triméthyle aluminium $Al(CH_3)_3$ dans un solvant aprotique tel que par exemple le tétrahydrofurane (THF).

* soit un alkylsélénole, généré *in situ* à partir de sélénium métal Se(0) et d'alkyle lithium, dans un solvant aprotique tel que par exemple le tétrahydrofurane (THF), puis mis en présence de chlorure d'ammonium

On opère au sein d'un solvant polaire aprotique tel que par exemple le THF.

Les réactions subséquentes conduisant aux différents composés de formule (I), soit acidification, estérification, amidification, salification, sont effectuées dans des conditions bien connues de l'homme du métier.

Les oligomères (dimères, trimères) et polymères, linéaires ou ramifiés, acycliques ou cycliques, obtenus entre deux ou plusieurs molécules de dérivés de formule (I) décrits dans l'invention par réaction d'estérification entre les fonctions alcool et acide carboxylique présentes le cas échéant, pris isolément ou en mélange, sont obtenus par condensation et déhydratation en analogie avec les exemples décrits dans Acta Chemica Scandinavica B, 1980, 34, 633-636 ou encore selon EP2238124.

Dans le cadre de la préparation d'esters séléno-méthyliques (RCOSeMe), Kosikowski et Ames décrivent l'obtention, en proportion minoritaire, d'un sous-produit non désiré qui est le 3-méthylséléno-2-hydroxy-propanoate d'éthyle [Kosikowski, AP and Ames A, 1978, *J. Org. Chem.*, 43(13):2735]. Bien que n'en faisant pas partie, ce composé est structuralement proche des composés de formule générale (I) décrits dans la présente invention.

Selon un troisième aspect l'invention a également pour objet l'utilisation d'au moins un composé sélénié de formule générale (I) tel que précédemment défini, comme agent pharmaceutique, en particulier comme agent anti-tumoral, seul ou associé à au moins un autre agent pharmaceutique et notamment à au moins un agent anti-tumoral.

Par agent anti-tumoral on entend tout agent ayant la propriété de traiter une tumeur ou communément appelée « cancer ».

Selon un quatrième aspect, l'invention concerne aussi une composition pharmaceutique comprenant au moins un principe pharmaceutiquement actif comprenant au moins un composé sélénié de formule générale (I), tel que précédemment défini, seul ou associé avec au moins un autre principe pharmaceutiquement actif.

En particulier, le composé sélénié de formule générale (I), tel que précédemment défini selon l'invention, permet de réaliser le traitement de tumeurs ou cancers, soit seul soit en association avec un ou plusieurs autres agents anti-cancéreux ou cytotoxiques connus, et soit en pré-administration soit en co-administration, tels que ceux notamment de la prostate, du foie, des reins, du pancréas, des poumons, du colon et de la peau ;

Comme autres agents anti-cancéreux, il est entendu les composés suivants : les inhibiteurs de métalloprotéinase de matrice tels que l'aminoglutéthimide, l'estramustine, le medroxyprogesteroneacetate, le leuprolide, le flutamide, le torémifène, le Zoladex; les inhibiteurs du VEGF, tels que les anticorps anti-VEGF (Avastin (R)) et les petites molécules comme le ZD6474 et le SU6668; le vatalanib, le BAY-43-9006, le SU11248, le CP-547632, et le CEP-7055; les inhibiteurs de l'EGFR, tels que le gefitinib, l'erlotinib, le ABX-EGF, l'EMD72000, le 11F8, et le cetuximab ; les inhibiteurs d'Eg5, tels que le SB-715992, le SB-743921, et le MKI-833; les inhibiteurs de PAN, tels que le canertinib, le EKB-569, le CI-1033, l'AEE-788, et le XL-647; les inhibiteurs de kinase, terls que le 2C4, le GW-572016, le Gleevec (R) et le dasatinib (Sprycel (R); le Casodex (R) (bicalutamide, Astra Zeneca), le tamoxifène; les inhibiteurs de MAPK kinase, les inhibiteurs de PI3 kinase, les inhibiteurs de PDGF, tels que l'imatinib; les inhibiteurs de récepteurs tyrosine kinases, les inhibiteurs de la signalisation de l'intégrine; la tubuline ; les agents agissant comme la vinblastine, la vincristine, la vinorelbine, vinflunine, le paclitaxel, le docétaxel, le 7-O-methylthiomethylpaclitaxel, le 4-désacétyl-4-methylcarbonatepaclitaxel, le C-4 méthyl carbonate paclitaxel, l'epothilone A, l'epothilone B, l'épothilone C, l'epothilone D, la désoxyépothilone A, la désoxyépothilone B, l'oxabicyclo [14.1.0], l'heptadécane-5-9-dione (ixabepilone), et des dérivés de ceux-ci; les inhibiteurs de CDK, les inhibiteurs du cycle cellulaire anti-prolifératifs, l'epidophyllotoxin, l'étoposide, le VM-26; les inhibiteurs de topoisomérase I ou II, tels que la camptothécine, le topotécan, la SN-38, la procarbazine, la mitoxantrone ; les complexes de coordination du platine tels que le cisplatine, le carboplatine et l'oxaliplatine; les inhibiteurs de croissance, les agents thérapeutiques anti-hormonal; la leucovorine; le tégafur; les antimétabolites tels que les antagonistes de la purine (par exemple la 6-thioguanine et la 6-mercaptopurine);

les antagonistes de la glutamine.

Comme autres agents cytotoxiques, il est entendu les composés suivants : le cyclophosphamide, la doxorubicine, la daunorubicine, la mitoxantrone, le melphalan, l'hexaméthyle mélamine, le thiotépa, la cytarabine, l'idatrexate, trimétrexate, la dacarbazine, la L-asparaginase, le bicalutamide, le leuprolide, les dérivés du pyridobenzoindole, les interférons, les interleukines.

Selon un cinquième aspect l'invention a pour objet les compositions pharmaceutiques renfermant les composés séléniés de formule générale (I) à titre de principe actif.

Selon ce cinquième aspect de la présente invention, les composés de formule générale (I) sont par exemple utilisés dans des quantités se situant entre 0,02% et 0,15% équivalents Sélénium (eq. Se) en poids de la préparation.

Selon ce cinquième aspect de la présente invention, les compositions pharmaceutiques comprennent un support pharmaceutiquement acceptable et une quantité thérapeutiquement efficace d'un ou plusieurs des composés de la présente invention ou d'un stéréoisomère, d'un tautomère, d'un sel pharmaceutiquement acceptable. Ces supports peuvent notamment consister en :

* une solution injectable ou buvable,

* un milieu solide composé d'un ou plusieurs excipients pouvant être sélectionnés parmi des vitamines, des antioxydants naturels tel que la L-ergothionéine, des sels minéraux, des mono-, di- ou polysaccharides, notamment l'acide folique, les vitamines B_6, E ou C, du lactose, de l'amidon. Ce milieu solide composé d'un ou plusieurs excipients tels que définis ci-dessus, et comprenant au moins l'un des composés de formule générale (I), peut être formulé sous la forme d'une gélule, d'un comprimé ou d'une poudre.

Selon ce cinquième aspect de la présente invention, les compositions pharmaceutiques renfermant au moins l'un des composés de formule générale (I) à titre de principe actif, peuvent selon le cas être administrées par voie orale, intraveineuse, par voie parentérale, par voie topique incluant la voie transdermique ou nasale ou oculaire, ou par inhalation. Les quantités des différents constituants de ces compositions, autres que les composés de formule générale (I), sont celles habituellement utilisées pour les applications citées.

La présente invention englobe toutes les combinaisons des aspects et / ou modes de réalisation de l'invention cités ci-dessus. Il est entendu que tout mode de réalisation de la présente invention peut être pris en conjonction avec un autre mode de réalisation pour décrire des modes de réalisation supplémentaires plus préférés. Il est également bien entendu que chaque élément individuel des modes de réalisation

préférés est son propre mode de réalisation préféré indépendant.

Description des figures :

Figure 1 : Schéma du procédé de synthèse des composés selon la formule générale (I)

Figure 2 : Pourcentage de viabilité des cellules DU145 et LS174T en fonction de la concentration en composé 4, après 96 heures de traitement

Figure 3 : Pourcentage de viabilité des cellules DU145, LS174T et HT-29 en fonction de la concentration en composé 10, après 96 heures de traitement

Figure 4 : Pourcentage de viabilité des cellules PC3, DU145, PANC-1 et MIA PaCa-2 en fonction de la concentration en composé 38, après 96 heures de traitement

Figure 5 : Pourcentage de viabilité des cellules HT-29, LS174T, HepG2 et MCF-7 en fonction de la concentration en composé 38, après 96 heures de traitement

Exemples

Les exemples ci-après, de même que le schéma du procédé de l'invention (Figure 1) et les autres figures, sont fournis simplement à titre d'illustration et ne sauraient, en aucune façon, limiter la portée de l'invention.

Dans les exemples, décrits ci-après, tous les pourcentages sont donnés en poids, la température est la température ambiante ou est donnée en degré Celsius, et la pression est la pression atmosphérique, sauf indication contraire.

Les réactifs utilisés sont disponibles commercialement chez des fournisseurs internationaux tel que SAF (France), Alfa Aesar , Fisher Scientific, TCI Europe, Bachem (Suisse) sauf les composés suivants, qui ont été préparés selon les protocoles cités : l'ester éthylique de l'oxirane-2-carboxylate (selon *Org . Synth.* 2006, 83, 162-169) ; l'ester *tert*-butylique de l'oxirane-2-carboxylate (selon *J. Am. Chem. Soc.* 2008, 130 (31), 10096-10102) l'ester benzylique du (*R*)-oxirane-2-carboxylate (*J. Org. Chem.* 1992, 57 (11), 3380-3387).

I. Exemples de préparation des composés selon l'invention

1a- Préparation des composés A par introduction du sélénium: Esters de l'acide 3-(alkylséléno)-2-hydroxy-propanoïque

Les composés A sont préparés par réaction d'un alkylsélénole R^1SeH ou d'un dialkyle aluminium alkylsélénolate $Al(R^1)_2SeR^1$ (généré *in situ* à partir du trialkyle aluminium et du sélénium élémentaire Se° selon *A. P. Kozikowski and A. Ames, J. Org. Chem. 1978, 43, 2735*), avec un alkyle oxirane carboxylate.

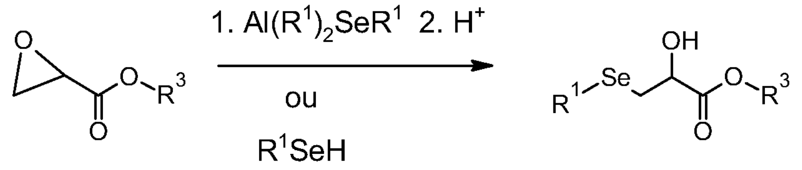

Composés A ($X = OR^3$)

Exemple A1 : Préparation de l'ester méthylique de l'acide 2-hydroxy-3-(méthylséléno)propanoïque (Composé 1) utilisant le diméthylaluminium méthylsélénolate

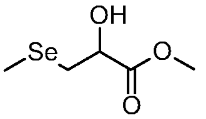

Composé 1

8,6 g (12,6 ml; 25,2 mmoles; 1,1 équiv.) d'une solution 2 M de triméthyle aluminium dans le toluène sont ajoutés goutte à goutte (temps d'addition 15 min) sous azote à 2,0 g (25,2 mmoles; 1,1 équiv.) de sélénium Se°. La suspension est agitée 15 min à température ambiante puis 2 h à reflux en milieu clos. Le milieu est laissé revenir à température ambiante puis refroidie à 0°C sous azote.

2,386 g (22,9 mmoles) de méthyle oxirane-2-carboxylate en solution dans 12 ml de dichlorométhane sont additionnés goutte à goutte au milieu réactionnel (temps d'addition 15 min). Le milieu est laissé sous agitation pendant 30 min à 0°C puis 16 h à température ambiant.

Le milieu réactionnel est refroidi à - 4°C pendant 15 min. 1,286 g (24,05 mmoles; 1,05 équiv.) d'ammonium chlorure dans 10 mL d'eau sont additionnés goutte à goutte très lentement au milieu réactionnel (temps d'addition 15 min) car un fort dégagement gazeux se produit (*Attention : génération et dégagement de méthane*). 20 mL de dichlorométhane sont additionnés goutte à goutte puis le milieu est laissé sous agitation pendant 10 min à froid. 20 mL d'une solution aqueuse saturée de

NH_4Cl sont additionnés goutte à goutte puis le milieu est laissé sous agitation pendant 10 min à froid.

Le milieu réactionnel est filtré sur Célite qui est rincée avec du dichlorométhane (7x20 mL). La phase organique est décantée et la phase aqueuse est extraite avec du dichlorométhane (3x20 mL). Les phases organiques sont regroupées, séchées avec Na_2SO_4, filtrées et concentrées.

L'huile jaune obtenue est distillée sous pression réduite de 8 mbar (125°C). On obtient 1,998 g (42 %) du composé **1** sous forme d'une huile jaune.

^{1}H-RMN ($CDCl_3$, 400 MHz) : δ (ppm) = 2,09 (s, 3H) ; 2,91 (m, 1H) ; 3,01 (m, 1H) ; 3,18 (m, 1H) ; 3,83 (s, 3H) ; 4,51 (m, 1H).

^{13}C-RMN ($CDCl_3$, 75 MHz) : δ (ppm) = 5,9; 29,9; 53,1; 70,7; 174,1

UPLC-MS (AP+) : 220,8 $(M+Na)^+$

Exemple A2 : Préparation de l'ester méthylique de l'acide 2-hydroxy-3-(méthylséléno)propanoïque (Composé 1) utilisant le méthylsélénol

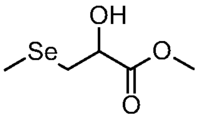

Composé 1

992 mg (12,5 mmoles; 1 éq.) de sélénium Se° sont mis en suspension sous azote dans 44 ml de THF. La suspension est refroidie à -3°C puis 6,2 ml (18,6 mmoles; 1,49 éq.) d'une solution 3 M de méthyllithium dans le diéthoxyméthane sont ajoutés goutte à goutte (temps d'addition 7 min). Le milieu complètement décoloré est agité 20 min à froid puis 802 mg (15 mmoles; 1,2 éq.) d'ammonium chlorure en solution dans 44 ml de méthanol sont ajoutés goutte à goutte. Le milieu est agité 20 min à froid puis 2,11 ml (15 mmoles; 1,2 éq.) de triéthylamine sont additionnés. Le milieu est agité 20 min à froid puis 1,71 g (16,25 mmoles; 1,3 éq.) de méthyl oxirane-2-carboxylate sont additionnés. Le milieu est laissé sous agitation pendant 1 h à 0°C puis 22 h à température ambiant.

Le milieu réactionnel est refroidi à 0°C pendant 15 min. 90 mL de dichlorométhane sont additionnés goutte à goutte puis le milieu est laissé sous agitation pendant 10 min à froid. 90 mL d'une solution aqueuse saturée de NH_4Cl sont additionnés goutte à goutte puis le milieu est laissé sous agitation pendant 10 min à froid. Le milieu est

dilué avec 90 mL d'eau. La phase organique est récupérée et la phase aqueuse est extraite avec du dichlorométhane (2x90 mL). Les phases organiques sont regroupées, séchées avec Na_2SO_4, filtrées et concentrées. L'huile jaune obtenue est purifiée sur colonne de silice (cyclohexane/acétate d'éthyle).

On obtient 330 mg (14 %) du composé **1**.

Exemple A3 : Préparation de l'ester méthylique de l'acide (*R*)-2-hydroxy-3-(méthylséléno)propanoïque (Composé 2)

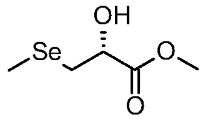

Composé 2

Le composé **2** est obtenu en utilisant les conditions de l'exemple A1 en partant de 4,959 g de méthyle (S)-oxirane-2-carboxylate. Après rinçage de la Célite et évaporation des solvants, l'huile obtenue est purifiée sur colonne de silice (cyclohexane/acétate d'éthyle).

On obtient 3,257 g (33 %) du composé **2** sous forme d'une huile jaune.

Le spectre ^{1}H RMN est identique à celui obtenu dans l'exemple A1.

$[\alpha]_D$: +11,5 (c = 1,0 ; MeOH)

Exemple A4 : Préparation de l'ester méthylique de l'acide (*S*)-2-hydroxy-3-(méthylséléno)propanoïque (Composé 3)

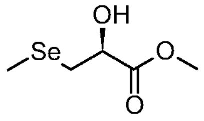

Composé 3

Le composé **3** est obtenu en utilisant les conditions de l'exemple A1 en partant de 7 g de méthyle (*R*)-oxirane-2-carboxylate. Après rinçage de la Célite et évaporation des solvants, l'huile obtenue est purifiée sur colonne de silice (cyclohexane/acétate d'éthyle).

On obtient 4,04 g (29 %) du composé **3** sous forme d'une huile orange.

Le spectre ^{1}H RMN est identique à celui obtenu dans l'exemple A1.

$[\alpha]_D$: -11,1 (c = 1,0 ; MeOH)

Exemple A5 : Préparation de l'ester éthylique de l'acide 2-hydroxy-3-(méthylséléno)propanoïque (Composé 4)

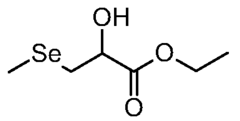

Composé 4

Le composé **4** est obtenu en utilisant les conditions de l'exemple A1 en partant de 1,56 g d'éthyle oxirane-2-carboxylate. Après rinçage de la Célite et évaporation des solvants, l'huile obtenue est purifiée sur colonne de silice (cyclohexane/acétate d'éthyle).

On obtient 1,188 g (38 %) du composé **4** sous forme d'une huile jaune.

^{1}H-RMN ($CDCl_3$, 400 MHz) : δ (ppm) = 1,35 (t, J=7,0 Hz, 3H) ; 2,10 (s, 3H) ; 2,91 (dd, J=13,0 Hz, J=5,5 Hz, 1H) ; 3,01 (dd, J=13,0 Hz, J=4,0 Hz, 1H) ; 3,16 (d, J=6,0 Hz, 1H) ; 4,3 (q, J=7,0 Hz, 2H) ; 4,5 (td, J=5,5 Hz, J=4,0 Hz, 1H).

^{13}C-RMN ($CDCl_3$, 75 MHz) : δ (ppm) = 6,0; 14,6; 29,9; 62,4; 70,8; 173,7

UPLC-MS (AP+) : 234,8 $(M+Na)^+$

Exemple A6 : Préparation de l'ester *tert*-butylique de l'acide 2-hydroxy-3-(méthylséléno)propanoïque (Composé 5)

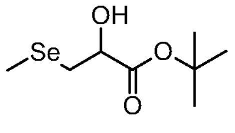

Composé 5

Le composé **5** est obtenu en utilisant les conditions de l'exemple A1 en partant de 1,0 g de *ter*-butyle oxirane-2-carboxylate. Après rinçage de la Célite et évaporation des solvants, l'huile obtenue est purifiée sur colonne de silice (cyclohexane/acétate d'éthyle).

On obtient 889 mg (54 %) du composé **5** sous forme d'une huile légèrement jaune.

^{1}H-RMN ($CDCl_3$, 400 MHz) : δ (ppm) = 1,53 (s, 9H) ; 2,13 (s, 3H) ; 2,87 (dd, J=13,0 Hz, J=5,5 Hz, 1H) ; 2,97 (dd, J=13,0 Hz, J=4,0 Hz, 1H) ; 3,19 (d, J=5,5 Hz, 1H) ; 4,39 (td, J=5,5 Hz, J=4,0 Hz, 1H).

Exemple A7 : Préparation de l'ester benzylique de l'acide (*S*)-(2-hydroxy-3-(méthylséléno)propanoïque (Composé 6)

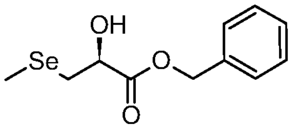

Composé 6

Le composé **6** est obtenu en utilisant les conditions de l'exemple A1 en partant de 622 mg de benzyle (*R*)-oxirane-2-carboxylate. Après rinçage de la Célite et évaporation des solvants, l'huile obtenue est purifiée sur colonne de silice (cyclohexane/acétate d'éthyle).

On obtient 325 mg (33 %) du composé **6** sous forme d'une huile légèrement jaune.

^{1}H-RMN ($CDCl_3$, 400 MHz) : δ (ppm) = 2,05 (s, 3H) ; 2,91 (dd, J=13,0 Hz, J=5,5 Hz, 1H) ; 3,02 (dd, J=13,0 Hz, J=4,5 Hz, 1H) ; 3,18 (d, J=5,5 Hz, 1H) ; 4,55 (td, J=5,5 Hz, J=4,5 Hz, 1H) , 5,26 (s, 2H) ; 7,41 (m, 5H).

Exemple A8 : Préparation de l'ester méthylique de l'acide 3-(éthylséléno)-2-hydroxypropanoïque (Composé 7)

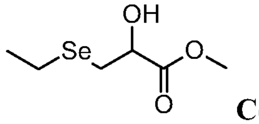

Composé 7

Le composé **7** est obtenu en utilisant les conditions de l'exemple A1 en partant de 2 g de sélénium Se°, 10,98 g d'une solution 25 % de triéthyle aluminium dans le toluène, et 2,386 g de méthyle oxirane-2-carboxylate. Après rinçage de la Célite et évaporation des solvants, l'huile obtenue est purifiée sur colonne de silice (cyclohexane/acétate d'éthyle).

On obtient 1,977 g (40 %) du composé **7** sous forme d'une huile jaune.

^{1}H-RMN ($CDCl_3$, 400 MHz) : δ (ppm) = 1,41 (t, J=7,5 Hz, 3H) ; 2,67 (q, J=7,5 Hz, 2H) ; 2,92 (dd, J=13,0 Hz, J=5,5 Hz, 1H) ; 3,02 (dd, J=13,0 Hz, J=4,5 Hz, 1H) ; 3,18 (d, J=4,5 Hz, 1H) ; 3,82 (s, 3H) , 4,5 (m, 1H).

^{13}C-RMN ($CDCl_3$, 75 MHz) : (ppm) = 16,2; 18,9; 27,8; 53,1; 70,8; 174,1

Exemple A9 : Préparation de l'ester méthylique de l'acide 2-hydroxy-3-(isobutylséléno)propanoïque (Composé 8)

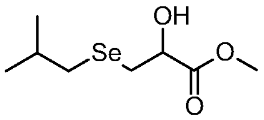

Composé 8

Le composé **8** est obtenu en utilisant les conditions de l'exemple A1 en partant de 2,0 g de sélénium Se°, 19,08 g d'une solution 25 % de triisobutyle aluminium dans le toluène et 2,386 g de méthyle oxirane-2-carboxylate. Après rinçage de la Célite et évaporation des solvants, l'huile obtenue est purifiée sur colonne de silice (cyclohexane/acétate d'éthyle).

On obtient 2,291 g (42 %) du composé **8** sous forme d'une huile jaune.

^{1}H-RMN ($CDCl_3$, 300 MHz) : δ (ppm) = 0,77 (d, J=6,5 Hz, 6H) ; 1,61 (m, 1H) ; 2,39 (d, J=7,0 Hz, 2H) ; 2,66 (dd, J=13,0 Hz, J=5,5 Hz, 1H) ; 2,76 (dd, J=13,0 Hz, J=4,5 Hz, 1H) ; 3,58 (s, 3H) ; 4,25 (m, 1H).

^{13}C-RMN ($CDCl_3$, 75 MHz) : (ppm) = 22,9; 29,0; 29,7; 35,7; 53,1; 70,7; 174,0

<u>1b- Préparation des composés A par estérification</u>

Les composés A peuvent être obtenus par introduction des réactifs séléniés, comme décrit dans le paragraphe 1a ci-dessus, mais également par estérification des composés B (pour leur préparation voir paragraphe 2 ci-après):

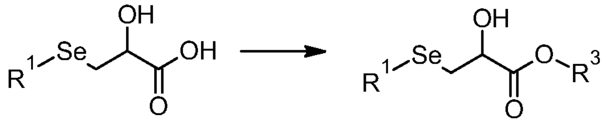

composés B composés A

Exemple A10: Préparation de l'ester éthylique de l'acide 2-hydroxy-3-(méthylséléno)propanoïque (Composé 4)

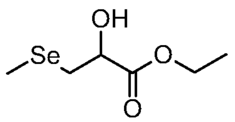

Composé 4

500 mg (2,68 mmoles; 1 éq.) de l'acide 2-hydroxy-3-(méthylséléno)propanoïque (**10**, paragraphe 2) sont mis en solution dans 11 mL d'éthanol absolu sous azote. 66 mg

(1,07 mmoles; 0,4 éq.) d'acide orthoborique sont ajoutés au milieu. Le milieu est laissé au reflux sous agitation et sous azote pendant 48 h.

33 mg (535 µmoles; 0,2 éq.) d'acide orthoborique sont ajoutés de nouveau au milieu. Le milieu est laissé au reflux pendant 24 h.

Le milieu est concentré à sec puis le concentrât est repris avec une solution aqueuse de NH4Cl demi saturée (40 mL). Le milieu est extrait par acétate d'éthyle (3x40 mL). Les phases organiques sont regroupées, séchées avec Na_2SO_4, filtrées et concentrées.

On obtient 446 mg (77 %) du composé **4** sous forme d'une huile jaune.

Le spectre ^{1}H RMN est identique à celui obtenu dans l'exemple A5.

Exemple A11 : Préparation de l'ester isopropyle de l'acide 2-hydroxy-3-(méthylséléno)propanoïque (Composé 9)

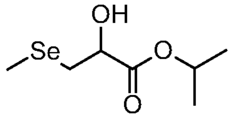

Composé 9

374 mg (2 mmoles; 1 éq.) de l'acide 2-hydroxy-3-(méthylséléno)propanoïque (**10**) sont mis en solution dans 8 mL de propanol-2 sous azote. 49 mg (800 µmoles; 0,4 éq.) d'acide orthoborique sont ajoutés au milieu. Le milieu est laissé au reflux sous agitation et sous azote pendant 48 h.

25 mg (400 µmoles; 0,2 éq.) d'acide orthoborique sont ajoutés de nouveau au milieu. Le milieu est laissé au reflux pendant 24 h.

Le milieu est concentré à sec puis le concentrât est repris avec une solution aqueuse de $NaHCO_3$ demi saturée (40 mL). Le milieu est extrait par acétate d'éthyle (3x40 mL). Les phases organiques sont regroupées, séchées avec Na_2SO_4, filtrées et concentrées. L'huile obtenue est purifiée sur colonne de silice (cyclohexane/acétate d'éthyle).

On obtient 285 mg (62 %) du composé **9** sous forme d'une huile jaune.

^{1}H-RMN ($CDCl_3$, 400 MHz) : δ (ppm) = 1,32 (d, J=6,0 Hz, 6H) ; 2,11 (s, 3H) ; 2,89 (dd, J=13,0 Hz, J=5,5 Hz, 1H) ; 2,99 (m, 1H) ; 3,20 (s, 1H) ; 4,45 (m, 1H) ; 5,14 (m, 1H).

^{13}C-RMN ($CDCl_3$, 75 MHz) : δ (ppm) = 6,0; 22,2; 29,8; 70,4; 70,9; 173,2

UPLC-MS (AP+) : 248,9 $(M+Na)^+$

2- <u>Préparation des composés B et C: les acides 3-(alkylséléno)-2-hydroxypropanoique et les sels correspondants</u>

Les composés B sont préparés par hydrolyse de la fonction ester des composés A, et les sels correspondants C sont obtenus par réaction des composés B avec des oxydes ou hydroxydes :

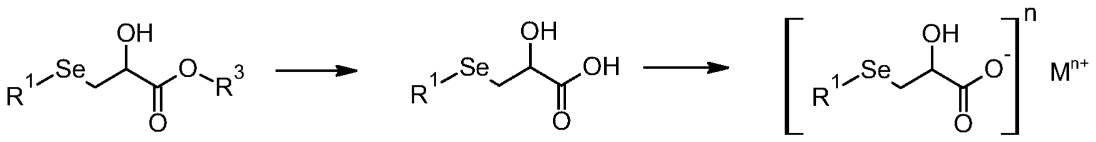

composés A **composés B** **composés C**

Exemple B1 : Préparation de l'acide 2-hydroxy-3-(méthylséléno)propanoïque (Composé 10) à partir de l'ester de méthyle

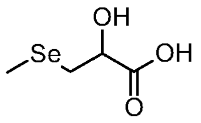

Composé 10

4,908 g (23,53 mmoles) de composé **1** sont solubilisés dans 14 ml de THF, 5 ml de méthanol et 5 ml d'eau déminéralisée. 47,1 ml (47,07 mmoles; 2 équiv.) d'une solution aqueuse d'hydroxyde de lithium à 1M sont ajoutés, la solution est agitée à température ambiante pendant 16 h.

Le pH du milieu est ajusté à 1 par l'ajout d'une solution d'acide chlorhydrique 2 M (14 mL). Le milieu est extrait à l'acétate d'éthyle (4x100 mL). Les phases organiques sont regroupées, séchées sur Na_2SO_4, filtrées et concentrées.

On obtient 4,177 g (95 %) du composé **10** sous forme d'un solide jaune pâle.

^{1}H-RMN (D_2O, 400 MHz) : δ (ppm) = 2,08 (s, 3H) ; 2,92 (dd, J=13,5 Hz, J=6,5 Hz, 1H) ; 3,03 (dd, J=13,5 Hz, J=4,5 Hz, 1H) ; 4,57 (dd, J=6,5 Hz, J=4,5 Hz, 1H).

^{1}H-RMN (DMSO, 400 MHz) : δ (ppm) = 1,99 (s, 3H) ; 2,70 (dd, J=12,5 Hz, J=6,5 Hz, 1H) ; 2,79 (dd, J=12,5 Hz, J=5,0 Hz, 1H) ; 4,18 (dd, J=6,5 Hz, J=5,0 Hz, 1H).

^{13}C-RMN ($CDCl_3$, 75 MHz) : δ (ppm) = 6,0; 29,6; 70,1; 178,0

UPLC-MS (AP-) : 182,6 $(M-H^+)$

Analyse élémentaire : $C_4H_8O_3Se$; Théorique : C (26,24 %) ; H (4,4 %) ; Trouvé : C (26,9 %) ; H (4,42 %)

Exemple B2 : Préparation de l'acide 2-hydroxy-3-(méthylséléno)propanoïque (Composé 10) à partir de l'ester *tert*-butylique

Le composé **10** est obtenu en utilisant les conditions de l'exemple B1 en partant de 100 mg de *ter*-butyle 2-hydroxy-3-(méthylséléno)propanoate (**5**).

On obtient 64 mg (82 %) du produit désiré sous forme d'un solide.

Le spectre ^{1}H RMN est identique à celui obtenu dans l'exemple B1.

Exemple B3 : Préparation de l'acide (*R*)-2-hydroxy-3-(méthylséléno)propanoïque (Composé 11)

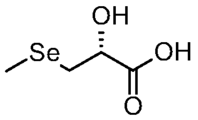

Composé 11

Le composé **11** est obtenu en utilisant les conditions de l'exemple B1 en partant de 2,117 g de méthyle (*R*)-2-hydroxy-3-(méthylséléno)propanoate (**2**).

On obtient 1,875 g (96 %) du produit désiré sous forme d'un solide jaune.

Le spectre ^{1}H RMN est identique à celui obtenu dans l'exemple B1.

$[\alpha]_D$: +1,07 (c = 6,0 ; EtOH)

Exemple B4 : Préparation de l'acide (S)-2-hydroxy-3-(méthylséléno)propanoïque (Composé 12) à partir de l'ester de méthyle

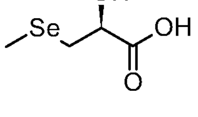

Composé 12

Le composé **12** est obtenu en utilisant les conditions de l'exemple B1 en partant de 2,95 g de méthyle (S)-2-hydroxy-3-(méthylséléno)propanoate (**3**).

On obtient 2,61 g (95 %) du produit désiré sous forme d'un solide jaune.

Le spectre ^{1}H RMN est identique à celui obtenu dans l'exemple B1.

$[\alpha]_D$: -1,03 (c = 6,0 ; EtOH)

Exemple B5 : Préparation de l'acide (*S*)-2-hydroxy-3-(méthylséléno)propanoïque (Composé 12) à partir de l'ester benzylique

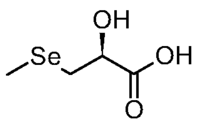

Composé 12

Le composé **12** est obtenu en utilisant les conditions de l'exemple B1 en partant de 494 mg de benzyle (*S*)-2-hydroxy-3-(méthylséléno)propanoate (**6**).

Après 16 h d'agitation à température ambiante, le milieu réactionnel est extrait à l'acétate d'éthyle (2x30 mL). Le pH du milieu est ajusté à 1 par l'ajout d'une solution aqueuse d'acide chlorhydrique à 2 M. Le milieu est extrait à l'acétate d'éthyle (2x30 mL). Les phases organiques de cette deuxième extraction sont regroupées, séchées sur Na_2SO_4, filtrées et concentrées.

On obtient 280 mg (84 %) du composé **12** sous forme d'un solide jaune pâle.

Le spectre ^{1}H RMN est identique à celui obtenu dans l'exemple B1.

Exemple B6 : Préparation de l'acide 3-éthylséléno-2-hydroxypropanoïque (Composé 13) à partir de l'ester de méthyle

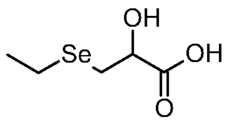

Composé 13

Le composé **13** est obtenu en utilisant les conditions de l'exemple B1 en partant de 500 mg de l'ester méthylique de l'acide 3-(éthylséléno)-2-hydroxypropanoïque (**7**).

On obtient 471 mg (100 %) du composé **13** sous forme d'un solide jaune pâle.

^{1}H-RMN ($CDCl_3$, 400 MHz) : δ (ppm) = 1,44 (t, J=7,5 Hz, 3H) ; 2,71 (q, J=7,5 Hz, 2H) ; 3,00 (dd, J=13,5 Hz, J=6,0 Hz, 1H) ; 3,11 (dd, J=13,5 Hz, J=4,5 Hz, 1H) ; 4,55 (dd, J=6,0 Hz, J=4,5 Hz, 1H).

^{13}C-RMN ($CDCl_3$, 75 MHz) : (ppm) = 16,1; 19,1; 27,6; 70,3; 177,9

UPLC-MS (AP-) : 197,2 ($M-H^+$)

Exemple B7 : Préparation de l'acide 2-hydroxy-3-(isobutylséléno)propanoïque (Composé 14) à partir de l'ester de méthyle

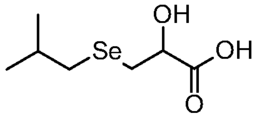

Composé 14

Le composé **14** est obtenu en utilisant les conditions de l'exemple B1 en partant de 500 mg de l'ester méthylique de l'acide 2-hydroxy-3-(isobutylséléno)propanoïque (**8**).

On obtient 423 mg (90 %) du composé **14** sous forme d'un solide jaune pâle.

^{1}H-RMN ($CDCl_3$, 400 MHz) : δ (ppm) = 1,03 (d, J=6,5 Hz, 6H) ; 1,88 (m, 1H) ; 1,95 (dd, J=7,0 Hz, 2H) ; 2,97 (dd, J=13,0 Hz, J=6,0 Hz, 1H) ; 3,08 (dd, J=13,0 Hz, J=4,5 Hz, 1H) ; 4,53 (m, 1H).

^{13}C-RMN ($CDCl_3$, 75 MHz) : (ppm) = 22,9; 28,7; 29,7; 35,8; 70,2; 177,9

UPLC-MS (AP-) : 225,3 ($M-H^+$)

Exemple C1 : Préparation du sel dicyclohexylammonium 2-hydroxy-3-(méthylséléno) propanoate (Composé 15)

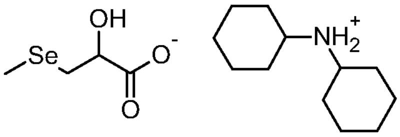

Composé 15

373 mg (2 mmoles) de l'acide 2-hydroxy-3-(méthylséléno)propanoïque sont solubilisés dans 2 ml d'acétone. 733 mg (805 µl; 4 mmoles; 2 éq.) de dicyclohexylamine sont ajoutés au milieu

Un précipité apparaît instantanément et est filtré, rincé avec de l'acétone (2x10 ml) puis avec un mélange acétate d'éthyle/cyclohexane 1/1 (2x10 ml).

On obtient 524 mg (70 %) du composé **15** sous forme d'un solide blanc.

^{1}H-RMN ($CDCl_3$, 400 MHz) : δ (ppm) = 1,26 (m, 7H) ; 1,50 (m, 4H) ; 1,68 (m, 2H) ; 1,84 (m, 4H) ; 2,06 (m, 4H) ; 2,12 (s, 3H) ; 2,91 (dd, J=12,5 Hz, J=6 Hz, 1H) ; 3,02 (m, 3H) ; 4,22 (m, 1H).

^{13}C-RMN ($CDCl_3$, 75 MHz) : δ (ppm) = 5,7; 25,2; 25,5; 29,4; 31,6; 53,2; 72,2; 177,7

Exemple C2 : Préparation du sel de sodium 2-hydroxy-3-(méthylséléno)propanoate (Composé 16)

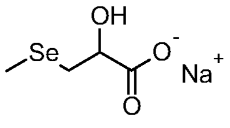

Composé 16

80 mg (2 mmoles) d'hydrure de sodium à 60 % dans l'huile minérale sont mis en suspension sous azote dans 2 ml de THF. 373 mg (2 mmoles) de l'acide 2-hydroxy-3-(méthylséléno)propanoïque solubilisés dans 2 ml de THF sont ajoutés goutte à goutte en 5 min.

Le milieu est refroidi dans un bain d'eau-glace puis du cyclohexane (3 ml) est ajouté. Un précipité se forme. Le milieu est filtré puis le solide est lavé avec du cyclohexane (3 ml) puis avec du TBME (3x3 ml).

On obtient 315 mg (75 %) du composé **16** sous forme d'un solide blanc cassé.

^{1}H-RMN (D_2O, 400 MHz) : δ (ppm) = 2,08 (s, 3H) ; 2,88 (dd, J=13,0 Hz, J=6,5 Hz, 1H) ; 2,99 (dd, J=13,0 Hz, J=4,0 Hz, 1H) ; 4,32 (dd, J=6,5 Hz, J=4,0 Hz, 1H).

^{13}C-RMN (D_2O, 75 MHz) : δ (ppm) = 4,8; 30,0; 71,7; 179,4

UPLC-MS (AP-) : 182,7 (M-Na^+)

Exemple C3 : Préparation du sel de magnésium bis(2-hydroxy-3-(méthylséléno)propanoate (Composé 17)

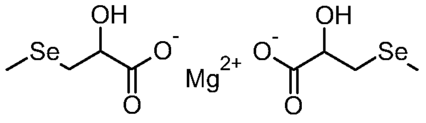

Composé 17

495 mg (2,65 mmoles) de l'acide 2-hydroxy-3-(méthylséléno)propanoïque sont solubilisés dans 1 ml d'eau déminéralisée puis 50 mg (1,25 mmoles) d'oxyde de magnésium sont ajoutés. Le milieu est agité à température ambiante pendant 16 h.

Le milieu est dilué avec de l'acétone (2 ml) puis le milieu est filtré sur verre fritté. Le solide est rincé avec de l'eau (0,5 mL) puis avec du TBME (2 ml). Le solide est séché sous vide.

On obtient 565 mg du composé **17** sous forme d'un solide blanc (quantitatif).

^{1}H-RMN (D_2O, 400 MHz) : δ (ppm) = 2,04 (s, 3H) ; 2,83 (dd, J=13,0 Hz, J=6,5 Hz, 1H) ; 2,95 (dd, J=13,0 Hz, J=4,0 Hz, 1H) ; 4,25 (dd, J=6,5 Hz, J=4,0 Hz, 1H).

Exemple C4 : Préparation du sel du zinc bis(2-hydroxy-3-(méthylséléno)propanoate (Composé 18)

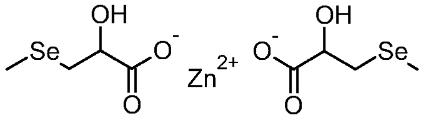

Composé 18

500 mg (2,65 mmoles) de l'acide 2-hydroxy-3-(méthylséléno)propanoïque sont solubilisés dans 1 ml d'eau déminéralisée puis 145 mg de carbonate de zinc basique sont ajoutés. 1 ml d'eau déminéralisée sont ajoutés puis le milieu est agité à température ambiante pendant 16 h.

Le milieu est dilué avec de l'acétone (2 ml) puis le milieu est filtré sur verre fritté. Le solide est rincé avec de l'eau (1 mL) puis avec de l'acétone (3x2 ml). Le solide est séché sous vide.

On obtient 496 mg (79 %) du composé **18** sous forme d'un solide blanc.

^{1}H-RMN (D_2O, 400 MHz) : δ (ppm) = 2,05 (s, 3H) ; 2,87 (dd, J=13,0 Hz, J=6,5 Hz, 1H) ; 2,98 (dd, J=13,0 Hz, J=4,0 Hz, 1H) ; 4,32 (dd, J=6,5 Hz, J=4,0 Hz, 1H).

Exemple C5 : Préparation du sel du calcium bis(2-hydroxy-3-(méthylséléno)propanoate (Composé 19)

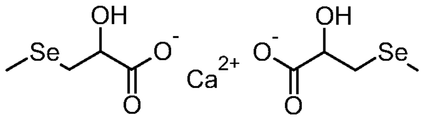

Composé 19

374 mg (2 mmoles) de l'acide 2-hydroxy-3-(méthylséléno)propanoïque sont solubilisés dans 0,9 ml d'eau déminéralisée puis 70 mg (945 µmoles) d'hydroxyde de calcium sont ajoutés. 1,1 ml d'eau déminéralisée sont ajoutés puis le milieu est agité à température ambiante pendant 16 h..

Le milieu est filtré sur verre fritté. Le solide est rincé avec de l'eau (2x2 mL) puis avec d'éther terbutylméthylique (TBME) (2x2 ml). Le solide est séché sous vide.

On obtient 74 mg (19 %) du produit désiré sous forme d'un solide blanc cassé.

Le filtrat est concentré à sec. Le résidu est trituré avec du TBME (5 ml). Le solide est séché sous vide.

On obtient 205 mg (53 %) du composé **19** sous forme d'un solide blanc cassé.

^{1}H-RMN (D_2O, 400 MHz) : δ (ppm) = 2,07 (s, 3H) ; 2,88 (dd, J=13,0 Hz, J=6,5 Hz, 1H) ; 2,99 (dd, J=13,0 Hz, J=4,0 Hz, 1H) ; 4,30 (dd, J=6,5 Hz, J=4,0 Hz, 1H).

^{13}C-RMN (D_2O, 300 MHz) : δ (ppm) = 4,8; 30,4; 72,1; 180,1

3- <u>Préparation des composés D : Amides dérivés de l'acide 2-hydroxy-3-(alkylséléno)propanoique</u>

Les composés D sont préparés soit par aminolyse des composés A, ou synthétisés à partir des composés B par couplage peptidique.

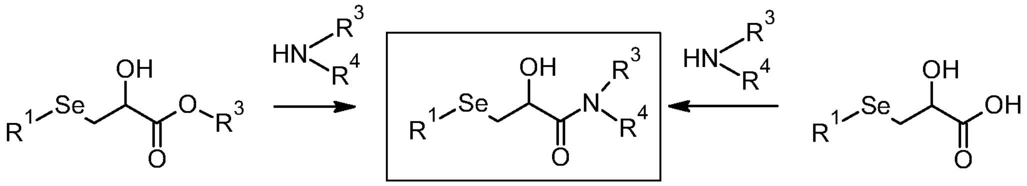

composés A composés D composés B

Exemple D1 : Préparation du 2-hydroxy-3-(méthylséléno)propanamide (Composé 20)

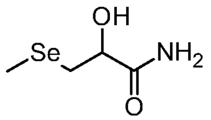

Composé 20

295 mg (1,5 mmoles) de composé **1** sont solubilisés dans 4,04 g (5,25 ml; 36,76 mmoles; 24 équiv.) d'une solution aqueuse d'ammoniaque à 20 %. Le milieu est agité à température ambiante pendant 22h.

Le milieu est évaporé à sec puis co-évaporé avec de l'acétate d'éthyle.

On obtient 284 mg (100 %) du composé **20** sous forme d'un solide blanc cassé.

^{1}H-RMN (MeOD, 400 MHz) : δ (ppm) = 2,11 (s, 3H) ; 2,85 (dd, J=13,0 Hz, J=7,0 Hz, 1H) ; 2,98 (dd, J=13,0 Hz, J=4,0 Hz, 1H) ; 4,3 (dd, J=7,0 Hz, J=4,0 Hz, 1H).

^{1}H-RMN (DMSO, 400 MHz) : δ (ppm) = 1,97 (s, 3H) ; 2,68 (dd, J=12,5 Hz, J=7,0 Hz, 1H) ; 2,80 (dd, J=12,5 Hz, J=4,0 Hz, 1H) ; 4,04 (m, 1H) ; 5,58 (d, J=5,5 Hz, 1H) ; 7,19 (d, J=15,5 Hz, 2H)

^{13}C-RMN (MeOD, 75 MHz) : δ (ppm) = 5,2; 31,0; 73,3; 179,2

UPLC-MS (AP+) : 183,8 $(M+H)^+$

UPLC-MS (AP+) : 206,0 $(M+Na)^+$

Exemple D2 : Préparation du N-cyclopropyl-2-hydroxy-3-(méthylséléno)propanamide (Composé 21)

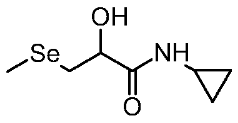

Composé 21

513 mg (2,5 mmoles) de composé **1** sont solubilisés dans 428 mg (519 µl; 7,5 mmoles; 3 équiv.) de cyclopropylamine. Le milieu est agité à 85°C pendant 72 h.
Le milieu est laissé revenir à température ambiante, évaporé à sec puis purifiée sur colonne de silice (dichlorométhane/méthanol).
On obtient 452 mg (79 %) du composé **21** sous forme d'un solide jaune pâle.

^{1}H-RMN ($CDCl_3$, 400 MHz) : δ (ppm) = 0,56 (m, 2H) ; 0,82 (m, 2H) ; 2,03 (s, 3H) ; 2,76 (m, 1H) ; 2,89 (dd, J=13,0 Hz, J=7,5 Hz, 1H) ; 3,08 (dd, J=13,0 Hz, J=4,5 Hz, 1H) ; 3,52 (d, J=3,5 Hz, 1H) ; 4,17 (m, 1H) ; 6,88 (s, 1H).
^{13}C-RMN ($CDCl_3$, 75 MHz) : δ (ppm) = 4,9; 6,8; 6,9; 22,7; 31,6; 69,9; 173,8
UPLC-MS (AP+) : 245,9 $(M+Na)^+$

Exemple D3 : Préparation du N-[2-(diméthylamino)éthyl]-2-hydroxy-3-(méthylséléno)propanamide (Composé 22)

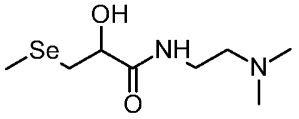

Composé 22

417 mg (2 mmoles) de composé **1** sont solubilisé dans 539 mg (674 µl; 6 mmoles; 3 équiv.) de N,N-diméthyléthylènediamine. Le milieu est agité à 85°C pendant 72 h.
Le milieu est laissé revenir à température ambiante, évaporé à sec puis purifiée sur colonne de silice (dichlorométhane/méthanol).
On obtient 460 mg (89 %) du composé **22** sous forme d'une huile orange.

^{1}H-RMN ($CDCl_3$, 400 MHz) : δ (ppm) = 2,05 (s, 3H) ; 2,28 (s, 6H) ; 2,48 (t, J=6,0 Hz, 2H) ; 2,91 (dd, J=13,0 Hz, J=7,0 Hz, 1H) ; 3,06 (dd, J=13,0 Hz, J=4,0 Hz, 1H) ; 3,40 (m, 2H) ; 4,26 (dd, J=7,0 Hz, J=4,0 Hz, 1H) ; 7,34 (m, 1H).
^{13}C-RMN ($CDCl_3$, 75 MHz) : δ (ppm) = 5,2; 31,6; 36,7; 45,6; 58,6; 71,0; 172,9

UPLC-MS (AP+) : 254,9 $(M+H)^+$

Exemple D4 : Préparation du 2-hydroxy-3-(méthylséléno)-1-(pyrrolidin-1-yl)propan-1-one (Composé 23)

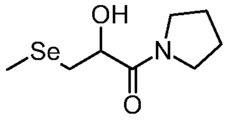

Composé 23

513 mg (2,5 mmoles) de composé **1** sont solubilisé dans 533 mg (616 µl; 7,5 mmoles; 3 équiv.) de pyrrolidine. Le milieu est agité à 85°C pendant 48 h.
Le milieu est laissé revenir à température ambiante, évaporé à sec puis purifiée sur colonne de silice (cyclohexane/acétate d'éthyle).
On obtient 402 mg (66 %) du composé **23** sous forme d'une huile orange.

^{1}H-RMN ($CDCl_3$, 400 MHz) : δ (ppm) = 1,85-2,07 (m, 4H) ; 2,11 (s, 3H) ; 2,77 (dd, J=13,0 Hz, J=7,0 Hz, 1H) ; 2,85 (dd, J=13,0 Hz, J=4,5 Hz, 1H) ; 3,45-3,65 (m, 4H) ; 3,78 (d, J=8,0 Hz, 1H) ; 4,45 (m, 1H).
^{13}C-RMN ($CDCl_3$, 75 MHz) : δ (ppm) = 5,8; 24,3; 26,5; 29,7; 46,7; 46,8; 70,2; 171,6
UPLC-MS (AP+) : 237,9 $(M+H)^+$
UPLC-MS (AP+) : 259,9 $(M+Na)^+$

Exemple D5 : Préparation du 2-hydroxy-3-(méthylséléno)-1-(pipéridin-1-yl)propan-1-one (Composé 24)

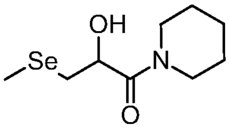

Composé 24

417 mg (2 mmoles) de composé **1** sont solubilisé dans 517 mg (600 µl; 6 mmoles; 3 équiv.) de pipéridine. Le milieu est agité à 85°C pendant 72 h.
Le milieu est laissé revenir à température ambiante, évaporé à sec puis purifiée sur colonne de silice (dichlorométhane/acétone).
On obtient 151 mg (29 %) du composé **24** sous forme d'une huile jaune.

^{1}H-RMN ($CDCl_3$, 400 MHz) : δ (ppm) = 1,56-1,74 (m, 6H) ; 2,13 (s, 3H) ; 2,72 (dd, J=13,0 Hz, J=7,0 Hz, 1H) ; 2,82 (dd, J=13,0 Hz, J=4,0 Hz, 1H) ; 3,40 (d, J=4,0 Hz, 2H) ; 3,62 (m, 2H) ; 4,03 (d, J=7,0 Hz, 1H) ; 4,61 (m, 1H).

^{13}C-RMN ($CDCl_3$, 75 MHz) : δ (ppm) = 6,0; 24,8; 25,8; 26,6; 30,4; 44,2; 46,5; 68,7; 171,3

UPLC-MS (AP+) : 251,9 $(M+H)^+$

UPLC-MS (AP+) : 273,9 $(M+Na)^+$

Exemple D6 : Préparation du 2-hydroxy-3-(méthylséléno)-1-(morpholin-4-yl)propan-1-one (Composé 25)

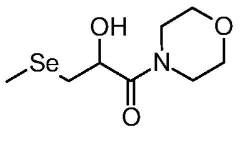

Composé 25

486 mg (2,4 mmoles) de composé **1** sont solubilisé dans 641 µl (7,2 mmoles; 3 équiv.) de morpholine. Le milieu est agité à 85°C pendant 72 h.

Le milieu est laissé revenir à température ambiante, évaporé à sec puis purifiée sur colonne de silice (dichlorométhane/acétone).

On obtient 262 mg (42 %) du composé **25** sous forme d'une huile jaune pâle.

^{1}H-RMN ($CDCl_3$, 400 MHz) : δ (ppm) = 2,12 (s, 3H) ; 2,79 (m, 2H) ; 3,52 (m, 2H) ; 3,65 (m, 1H) ; 3,74 (m, 5H) ; 3,82 (d, J=8,0 Hz, 1H) ; 4,59 (m, 1H).

^{13}C-RMN ($CDCl_3$, 75 MHz) : δ (ppm) = 6,1; 30,1; 43,3; 46,2; 66,8; 67,1; 68,6; 171,8

UPLC-MS (AP+) : 253,9 $(M+H)^+$

UPLC-MS (AP+) : 276,0 $(M+Na)^+$

Exemple D7 : Préparation du N,N-diéthyl-2-hydroxy-3-(méthylséléno)-propanamide (Composé 26)

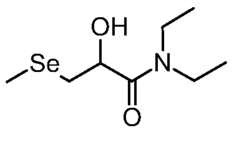

Composé 26

417 mg (2 mmoles) de composé **1** sont solubilisé dans 443 mg (633 µl;6 mmoles; 3 équiv.) de diéthylamine. Le milieu est agité à 85°C pendant 72 h.

Le milieu est laissé revenir à température ambiante, évaporé à sec puis purifiée sur colonne de silice (dichlorométhane/méthanol).

On obtient 105 mg (19 %) du composé **26** sous forme d'une huile orange.

^{1}H-RMN ($CDCl_3$, 400 MHz) : δ (ppm) = 1,38 (t, J=7,0 Hz, 6H) ; 2,10 (s, 3H) ; 2,91 (dd, J=12,5 Hz, J=6,0 Hz, 1H) ; 3,02 (m, 5H) ; 4,33 (m, 1H).

Exemple D8 : Préparation du 2-hydroxy-N-(2-hydroxyéthyl)-3-(méthylséléno)-propanamide (Composé 27)

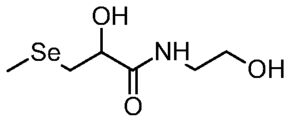

Composé 27

250 mg (1,22 mmoles) de composé **1** sont solubilisé dans 228 mg (225 µl; 3,66 mmoles; 3 équiv.) d'éthanolamine. Le milieu est agité à 85°C pendant 72 h.

Le milieu est laissé revenir à température ambiante, évaporé à sec puis purifiée sur colonne de silice (dichlorométhane/méthanol).

On obtient 203 mg (72 %) du composé **27** sous forme d'une huile verdâtre.

^{1}H-RMN ($CDCl_3$, 400 MHz) : δ (ppm) = 2,06 (s, 3H) ; 2,92 (dd, J=13,0 Hz, J=7,5 Hz, 1H) ; 2,95 (s, 1H) ; 3,11 (dd, J=13,0 Hz, J=4,0 Hz, 1H) ; 3,49 (m, 2H) ; 3,65 (d, J=4,5 Hz, 1H) ; 3,78 (m, 2H) ; 4,26 (m, 1H) ; 7,26 (s, 1H).

^{13}C-RMN ($CDCl_3$, 75 MHz) : δ (ppm) = 5,2; 31,2; 42,5; 62,2; 70,6; 173,9

UPLC-MS (AP+) : 227,9 $(M+H)^+$

UPLC-MS (AP+) : 249,8 $(M+Na)^+$

Exemple D9 : Préparation du [(2*RS*)-2-hydroxy-3-méthylsélénopropanoyl]-(*S*)-alanine (Composé 29)

D9.1. Préparation de l'ester *tert*-butylique du [(2*RS*)-2-hydroxy-3-méthylsélénopropanoyl]-(*S*)-alanine (Composé 28)

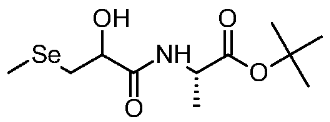

Composé 28

100 mg (546 µmoles) de l'acide 2-hydroxy-3-(méthylséléno)propanoïque (**10**) sont solubilisés sous azote dans 5 ml de dichlorométhane. Le milieu est refroidi à 0°C puis 183 mg (600 µmoles; 1,1 éq.) de 3-(diethoxyphosphoryloxy)-1,2,3-benzotriazin-4(3H)-one sont ajoutés. Le milieu est agité pendant 30 min à 0°C puis 1h30 à température ambiante. Le milieu est refroidi à 0°C puis 99 mg (546 µmoles; 1 éq.) du

chlorhydrate de l'ester *tert*-butylique de la (*S*)-alanine et 156 mg (200 µl; 1,20 mmoles; 2,2 éq.) de N,N-diisopropyléthylamine sont ajoutés. Le milieu est agité pendant 30 min à 0°C puis 16h à température ambiante.

Le milieu réactionnel est dilué avec du dichlorométhane (25 ml) puis le milieu est lavé avec une solution aqueuse d'acide chlorhydrique 1N (2x10 mL) puis avec une solution aqueuse de $NaHCO_3$ (1N, 2x10 mL) puis avec une solution aqueuse de NaCl saturée (10 mL). La phase organique est séchée sur Na_2SO_4, filtrée et concentrée. Le résidu est purifié sur colonne de silice (cyclohexane/acétate d'éthyle).

On obtient 116 mg (67 %) du composé **28** sous forme d'une huile incolore.

^{1}H-RMN ($CDCl_3$, 400 MHz) mélange 50/50 de 2 diastéréoisomères **28a** (*R*,*S*) et **28b** (*S*,*S*) : δ (ppm) = 1,42 et 1,43 (2d, J=3,5 Hz, 3H) ; 1,49 et 1,50 (2s, 9H) ; 2,04 et 2,05 (2s, 3H) ; 2,92 (dd, J=13,0 Hz, J=7,5 Hz, 1H) ; 3,09 (m, 1H) ; 3,43 (d, J=3,5 Hz, 1H) ; 4,21 (m, 1H) ; 4,48 (m, 1H) ; 7,27 et 7,33 (2d, J=6,5 Hz, 1H).

UPLC-MS (AP-) : 309,8 (M-H+)

UPLC-MS (AP+) : 333,9 $(M+Na)^+$

D9.2 : Préparation du [(2*RS*)-2-hydroxy-3-méthylsélénopropanoyl]-(*S*)-alanine (Composé 29)

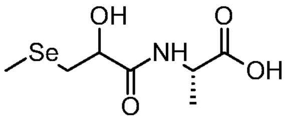

Composé 29

Le composé **29** est obtenu en utilisant les conditions de l'exemple B1 en partant de 58 mg de l'ester **28**.

On obtient 40 mg (81 %) du composé **29** sous forme d'une huile incolore.

^{1}H-RMN ($CDCl_3$, 400 MHz) mélange 50/50 de 2 diastéréoisomères **29a** (*R*,*S*) et **29b** (*S*,*S*) : δ (ppm) = 1,51 et 1,53 (2d, J=3,0 Hz, 3H) ; 2,05 et 2,06 (2s, 3H) ; 2,90 (m, 1H) ; 3,10 (m, 1H) ; 3,52 (s, 1H) ; 4,24 et 4,31 (2dd, J=8,0 Hz, J=4,0 Hz, 1H) ; 4,61 (m, 1H) ; 7,36 et 7,43 (2d, J=7,5 Hz, 1H).

Exemple D10 : Préparation du [(2*RS*)-2-hydroxy-3-méthylsélénopropanoyl]-(*S*)-méthionine (Composé 31)

D10.1. Préparation de l'ester méthylique du [(2*RS*)-2-hydroxy-3-méthylsélénopropanoyl]-(*S*)-méthionine (Composé 30)

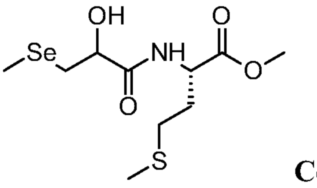

Composé 30

Le composé **30** est obtenu en utilisant les conditions de l'exemple D9 en partant de 500 mg (2,65 mmoles; 1 éq.) de l'acide 2-hydroxy-3-(méthylséléno)propanoïque (**10**) et 557 mg (2,65 mmoles; 1 éq.) de l'ester méthylique du chlorhydrate de la (*S*)-méthionine. On obtient 549 mg (60 %) du produit désiré sous forme d'une huile légèrement jaune.

^{1}H-RMN ($CDCl_3$, 400 MHz) mélange 50/50 de 2 diastéréoisomères **30a** (*R*,*S*) et **30b** (*S*,*S*) : δ (ppm) = 2,05 (m, 4H) ; 2,13 (m, 3H) ; 2,23 (m, 1H) ; 2,56 (t, J=7,5 Hz, 2H) ; 2,92 (m, 1H) ; 3,09 (m, 1H) ; 3,45 (m, 1H) ; 3,79 et 3,80 (2s, 3H) ; 4,25 (m, 1H) ; 4,75 (m, 1H) ; 7,39 (m, 1H).

D10.2. : Préparation du [(2*RS*)-2-hydroxy-3-méthylsélénopropanoyl]-(*S*)-méthionine (Composé 31)

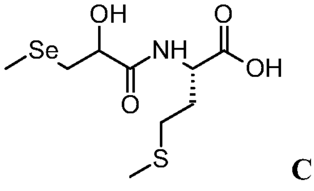

Composé 31

Le composé **31** est obtenu en utilisant les conditions de l'exemple B1 en partant de 445 mg du composé **30**.

Après 16 h d'agitation à température ambiante, le milieu réactionnel est dilué dans l'eau (40 ml) puis extrait à l'acétate d'éthyle (2x25 mL). Le pH du milieu est ajusté à 1 par l'ajout d'une solution aqueuse d'acide chlorhydrique 2 M. Le milieu est extrait à l'acétate d'éthyle (4x25 mL). Les phases organiques de cette deuxième extraction sont regroupées, séchées sur Na_2SO_4, filtrées et concentrées.

On obtient 400 mg (92 %) du produit désiré sous forme d'une huile jaune pâle.

^{1}H-RMN ($CDCl_3$, 400 MHz) mélange 50/50 de 2 diastéréoisomères **31a** (*R*,*S*) et **31b** (*S*,*S*) : δ (ppm) = 2,05 et 2,08 (2s, 3H) ; 2,11 (m, 1H) ; 2,15 (s, 3H) ; 2,27 (m, 1H) ; 2,62 (t, J=7,5 Hz, 2H) ; 2,91 (m, 1H) ; 3,10 (m, 1H) ; 4,28 et 4,38 (2dd, J=8,0 Hz, J=4,5 Hz, 1H) ; 4,75 (m, 1H) ; 7,51 et 7,57 (2d, J=8,0 Hz, 1H).

Exemple D11 : Préparation de la [(2*R*)-2-hydroxy-3-méthylsélénopropanoyl]-(*S*)-méthionine (Composé 31a)

D11.1. Préparation de l'ester méthylique de la [(2*R*)-2-hydroxy-3-méthylsélénopropanoyl]-(*S*)-méthionine (Composé 30a)

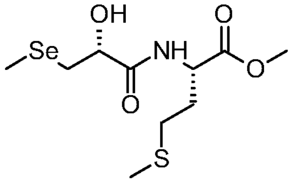

Composé 30a

Le composé **30a** est obtenu en utilisant les conditions de l'exemple D9 en partant de 100 mg (513 µmoles; 1 éq.) de l'acide (*R*)-2-hydroxy-3-(méthylséléno)propanoïque (**11**) et 108 mg (513 µmoles; 1 éq.) de l'ester méthylique du chlorhydrate de la (*S*)-méthionine. On obtient 89 mg (50 %) du produit désiré sous forme d'une huile incolore.

^{1}H-RMN ($CDCl_3$, 400 MHz) : δ (ppm) = 2,06 (s, 3H) ; 2,07 (m, 1H) ; 2,13 (s, 3H) ; 2,22 (m, 1H) ; 2,57 (t, J=7,5 Hz, 2H) ; 2,93 (dd, J=13,0 Hz, J=7,5 Hz, 1H) ; 3,09 (dd, J=13,0 Hz, J=4,5 Hz, 1H) ; 3,40 (d, J=4,0 Hz, 1H) ; 3,80 (s, 3H) ; 4,28 (m, 1H) ; 4,75 (m, 1H) ; 7,40 (d, J=8,0 Hz, 1H).

D11.2. Préparation de la [(2*R*)-2-hydroxy-3-méthylsélénopropanoyl]-(*S*)-méthionine (Composé 31a)

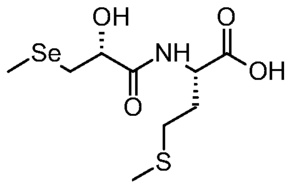

Composé 31a

Le composé **31a** est obtenu en utilisant les conditions de l'exemple D10.2 en partant de 89 mg du composé **30a**.

On obtient 82 mg (99 %) du produit désiré sous forme d'une huile incolore.

^{1}H-RMN ($CDCl_3$, 400 MHz) : δ (ppm) = 2,08 (s, 3H) ; 2,11 (m, 1H) ; 2,15 (s, 3H) ; 2,28 (m, 1H) ; 2,63 (m, 2H) ; 2,91 (dd, J=13,0 Hz, J=7,0 Hz, 1H) ; 3,08 (dd, J=13,0 Hz, J=4,0 Hz, 1H) ; 4,39 (dd, J=7,0 Hz, J=4,0 Hz, 1H) ; 4,74 (m, 1H) ; 7,59 (d, J=8,0 Hz, 1H).

Exemple D12 : Préparation de la [(2*S*)-2-hydroxy-3-méthylsélénopropanoyl]-(*S*)-méthionine (Composé 31b)

D12.1. Préparation de l'ester méthylique de la [(2*S*)-2-hydroxy-3-méthylsélénopropanoyl]-(*S*)-méthionine (Composé 30b)

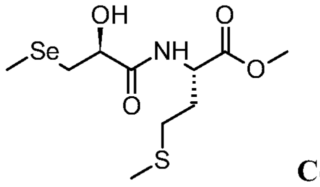

Composé 30b

Le composé **30b** est obtenu en utilisant les conditions de l'exemple D9 en partant de 100 mg (535 µmoles; 1 éq.) de l'acide (*S*)-2-hydroxy-3-(méthylséléno)propanoïque (**12**) et 112 mg (535 µmoles; 1 éq.) de l'ester méthylique du chlorhydrate de la (*S*)-méthionine. On obtient 115 mg (57 %) du produit désiré sous forme d'une huile incolore.

^{1}H-RMN ($CDCl_3$, 400 MHz) : δ (ppm) = 2,04 (s, 3H) ; 2,07 (m, 1H) ; 2,13 (s, 3H) ; 2,23 (m, 1H) ; 2,57 (t, J=7,5 Hz, 2H) ; 2,92 (dd, J=13,0 Hz, J=8,0 Hz, 1H) ; 3,10 (dd, J=13,0 Hz, J=4,5 Hz, 1H) ; 3,42 (d, J=4,5 Hz, 1H) ; 3,79 (s, 3H) ; 4,22 (m, 1H) ; 4,75 (m, 1H) ; 7,35 (d, J=8,0 Hz, 1H).

D12.2. Préparation de la [(2*S*)-2-hydroxy-3-méthylsélénopropanoyl]-(*S*)-méthionine (Composé 31b)

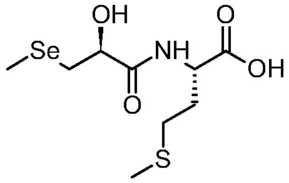

Composé 31b

Le composé **31b** est obtenu en utilisant les conditions de l'exemple D10.2 en partant de 115 mg du composé **30b**.

On obtient 98 mg (98 %) du produit désiré sous forme d'une huile jaune.

^{1}H-RMN ($CDCl_3$, 400 MHz) : δ (ppm) = 2,05 (s, 3H) ; 2,10 (m, 1H) ; 2,15 (s, 3H) ; 2,28 (m, 1H) ; 2,62 (t, J=7,5 Hz, 2H) ; 2,91 (dd, J=13,0 Hz, J=8,0 Hz, 1H) ; 3,11 (dd, J=13,5 Hz, J=4,5 Hz, 1H) ; 4,27 (dd, J=8,0 Hz, J=4,5 Hz, 1H) ; 4,75 (m, 1H) ; 7,48 (d, J=8,0 Hz, 1H).

Exemple D13 : Préparation de l'ester méthylique de la N(α)-[(2*RS*)-2-hydroxy-3-méthylsélénopropanoyl]-N(ω)-*tert*-butoxycarbonyl-(*S*)-lysine (Composé 32)

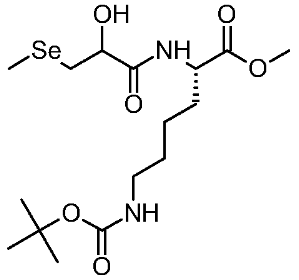

Composé 32

Le composé **32** est obtenu en utilisant les conditions de l'exemple D9 en partant de 100 mg (535 μmoles; 1 éq.) de l'acide 2-hydroxy-3-(méthylséléno)propanoïque (**10**) et 162 mg (546 μmoles; 1 éq.) de l'ester méthylique du chlorhydrate de la N(ω)-*tert*-butoxycarbonyl-(*S*)-lysine.

On obtient 171 mg (73 %) du produit désiré sous forme d'une huile incolore.

^{1}H-RMN ($CDCl_3$, 400 MHz) mélange 50/50 de 2 diastéréoisomères (*R*,*S*) et (*S*,*S*) : δ (ppm) = 1,37 (m, 2H) ; 1,44 (s, 9H) ; 1,48 (m, 2H) ; 1,73 (m, 1H) ; 1,89 (m, 1H) ; 2,02 et 2,04 (2s, 3H) ; 2,89 (m, 1H) ; 3,07 (m, 3H) ; 3,74 et 3,75 (s, 3H) ; 3,85 (m, 1H) ; 4,26 (m, 1H) ; 4,59 (m, 1H) ; 4,69 (m, 1H) ; 7,30 (m, 1H).

UPLC-MS (AP+) : 449,0 $(M+Na)^+$

Exemple D14 : Préparation de l'ester méthylique de la N(α)-[(2*RS*)-2-hydroxy-3-méthylsélénopropanoyl]-N(ω)-fluorenylméthyloxycarbonyl-(*S*)-lysine (Composé 33)

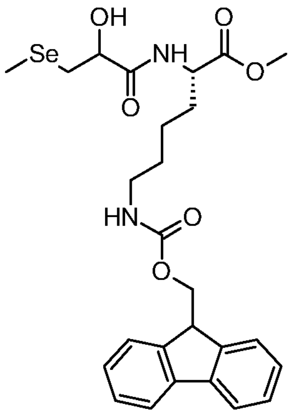

Composé 33

Le composé **33** est obtenu en utilisant les conditions de l'exemple D9 en partant de 200 mg (1,059 mmoles; 1 éq.) de l'acide 2-hydroxy-3-(méthylséléno)propanoïque (**10**) et 448 mg (1,059 mmoles; 1 éq.) de l'ester méthylique du chlorhydrate de la N(ω)-fluorenylméthyloxycarbonyl-(*S*)-lysine.

On obtient 430 mg (59 %) du produit désiré sous forme d'une huile incolore.

^{1}H-RMN ($CDCl_3$, 400 MHz) : δ (ppm) = 1,42 (m, 2H) ; 1,58 (m, 2H) ; 1,76 (m, 2H) ; 1,91 (m, 1H) ; 2,03 (s, 3H) ; 2,92 (m, 1H) ; 3,10 (m, 1H) ; 3,21 (m, 2H) ; 3,78 (s, 3H) ; 4,25 (m, 2H) ; 4,43 (m, 2H) ; 4,64 (m, 1H) ; 4,90 (m, 1H) ; 7,25 (m, 1H) ; 7,35 (m, 2H) ; 7,42 (m, 2H) ; 7,63 (m, 2H) ; 7,80 (m, 2H).

UPLC-MS (AP+) : 571,3 $(M+Na)^+$

Exemple D15 : Préparation de l'ester benzylique de la N(α)-[(2*RS*)-2-hydroxy-3-méthylsélénopropanoyl]-N(ω)-benzyloxycarbonyl-(*S*)-lysine (Composé 34)

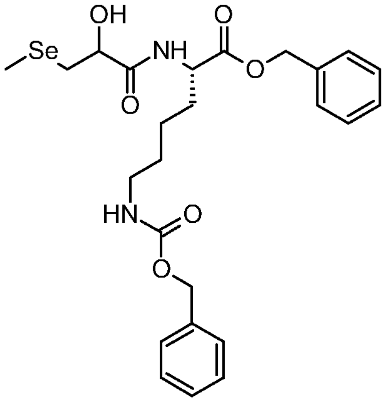

Composé 34

Le composé **34** est obtenu en utilisant les conditions de l'exemple D9 en partant de 400 mg (2,12 mmoles; 1 éq.) de l'acide 2-hydroxy-3-(méthylséléno)propanoïque (**10**) et 862 mg (2,12 mmoles; 1 éq.) de l'ester benzylique du chlorhydrate de la N(ω)-benzyloxycarbonyl-(*S*)-lysine.

On obtient 944 mg (76 %) du produit désiré sous forme d'une huile jaune.

^{1}H-RMN ($CDCl_3$, 400 MHz) mélange 50/50 de 2 diastéréoisomères **34a** (*R*,*S*) et **34b** (*S*,*S*) : δ (ppm) = 1,35 (m, 2H) ; 1,51 (m, 2H) ; 1,75 (m, 2H) ; 1,91 (m, 1H) ; 2,00 et 2,03 (2s, 3H) ; 2,89 (m, 1H) ; 3,07 (m, 1H) ; 3,17 (m, 2H) ; 3,37 et 3,49 (2s, 1H) ; 4,23 (m, 1H) ; 4,67 (m, 1H) ; 4,81 (m, 1H) ; 5,12-5,26 (m, 4H) ; 7,33-7,43 (m, 10H).

Exemple D16 : Préparation de la [(2*RS*)-2-hydroxy-3-méthylsélénopropanoyl]-(*S*)- (Composé 36)

D16.1. Préparation de l'ester méthylique de la [(2*RS*)-2-hydroxy-3-méthylsélénopropanoyl]-(*S*)-sélénométhionine (Composé 35)

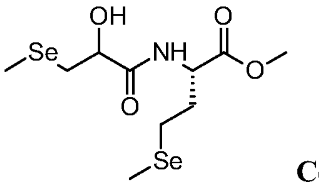

Composé 35

Le composé **35** est obtenu en utilisant les conditions de l'exemple D9 en partant de 136 mg (720 µmoles; 1 éq.) de l'acide 2-hydroxy-3-(méthylséléno)propanoïque (**10**) et 187 mg (720 µmoles; 1 éq.) de l'ester méthylique du chlorhydrate de la (*S*)-sélénométhionine.

On obtient 180 mg (63 %) du produit désiré sous forme d'une huile incolore.

^{1}H-RMN ($CDCl_3$, 400 MHz) mélange 50/50 de 2 diastéréoisomères **35a** (*R*,*S*) et **35b** (*S*,*S*) : δ (ppm) = 2,03 (s, 3H) ; 2,04 et 2,07 (2s, 3H) ; 2,13 (m, 1H) ; 2,28 (m, 1H) ; 2,57 (t, J=7,5 Hz, 2H) ; 2,92 (m, 1H) ; 3,10 (m, 1H) ; 3,38 (m, 1H) ; 3,79 et 3,80 (2s, 3H) ; 4,25 (m, 1H) ; 4,75 (m, 1H) ; 7,33 et 7,37 (2d, J=8,5 Hz, 1H).

D16.2. Préparation de la [(2*RS*)-2-hydroxy-3-méthylsélénopropanoyl]-(*S*)-sélénométhionine (Composé 36)

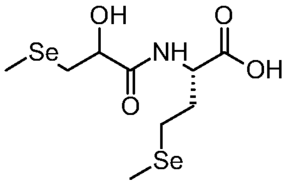

Composé 36

Le composé **36** est obtenu en utilisant les conditions de l'exemple B1 en partant de 130 mg du composé **35**.

Après 16 h d'agitation à température ambiante, le milieu réactionnel est dilué dans l'eau (20 ml) puis extrait à l'acétate d'éthyle (2x10 mL). Le pH du milieu est ajusté à 1 par l'ajout d'une solution aqueuse d'acide chlorhydrique 2 M. Le milieu est extrait à l'acétate d'éthyle (3x10 mL). Les phases organiques de cette deuxième extraction sont regroupées, séchées sur Na_2SO_4, filtrées et concentrées.

On obtient 112 mg (89 %) du produit désiré sous forme d'une huile jaune.

^{1}H-RMN ($CDCl_3$, 400 MHz) mélange 50/50 de 2 diastéréoisomères **36a** (*R*,*S*) et **36b** (*S*,*S*): δ (ppm) = 2,05 (s, 3H) ; 2,08 (s, 3H) ; 2,17 (m, 1H) ; 2,33 (m, 1H) ; 2,63 (m, 2H) ; 2,92 (m, 1H) ; 3,10 (m, 1H) ; 4,26 et 4,35 (2dd, J=8,0 Hz, J=4,5 Hz, 1H) ; 4,75 (m, 1H) ; 7,43 et 7,49 (2d, J=8,0 Hz, 1H).

Exemple D17 : Préparation de la [(2*R*)-2-hydroxy-3-méthylsélénopropanoyl]-(*S*)-sélénométhionine (Composé 36a)

D17.1. Préparation de l'ester méthylique de la [(2*R*)-2-hydroxy-3-méthylsélénopropanoyl]-(*S*)-sélénométhionine (Composé 35a)

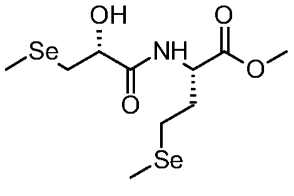

Composé 35a

Le composé **35a** est obtenu en utilisant les conditions de l'exemple D9 en partant de 185 mg (950 µmoles; 1 éq.) de l'acide (*R*)-2-hydroxy-3-(méthylséléno)propanoïque (**11**) et 260 mg (950 µmoles; 1 éq.) de l'ester méthylique du chlorhydrate de la (*S*)-sélénométhionine.

On obtient 233 mg (62 %) du produit désiré sous forme d'une huile jaune pâle.

^{1}H-RMN ($CDCl_3$, 400 MHz) : δ (ppm) = 2,03 (s, 3H) ; 2,07 (s, 3H) ; 2,12 (m, 1H) ; 2,29 (m, 1H) ; 2,57 (m, 2H) ; 2,93 (dd, J=13,0 Hz, J=7,5 Hz, 1H) ; 3,09 (dd, J=13,0 Hz, J=4,5 Hz, 1H) ; 3,32 (m, 1H) ; 3,80 (s, 3H) ; 4,27 (m, 1H) ; 4,75 (m, 1H) ; 7,37 (d, J=8,0 Hz, 1H).

D17.2. Préparation de la [(2*R*)-2-hydroxy-3-méthylsélénopropanoyl]-(*S*)-sélénométhionine (Composé 36a)

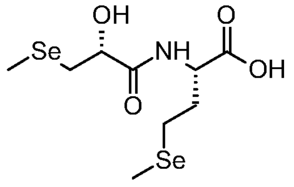

Composé 36a

Le composé **36a** est obtenu en utilisant les conditions de l'exemple B1 en partant de 183 mg du composé **35a**.

Le milieu réactionnel est dilué dans l'eau (20 ml) puis extrait à l'acétate d'éthyle (2x10 mL). Le pH du milieu est ajusté à 1 par l'ajout d'une solution aqueuse d'acide chlorhydrique 2 M. Le milieu est extrait à l'acétate d'éthyle (3x10 mL). Les phases organiques sont regroupées, séchées sur Na_2SO_4, filtrées et concentrées.

On obtient 178 mg (100 %) du produit désiré sous forme d'un solide crème.

^{1}H-RMN ($CDCl_3$, 400 MHz) : δ (ppm) = 2,05 (s, 3H) ; 2,08 (s, 3H) ; 2,18 (m, 1H) ; 2,35 (m, 1H) ; 2,64 (m, 2H) ; 2,91 (dd, J=13,0 Hz, J=7,0 Hz, 1H) ; 3,09 (dd, J=13,0 Hz, J=4,0 Hz, 1H) ; 4,38 (dd, J=7,0 Hz, J=4,0 Hz, 1H) ; 4,74 (m, 1H) ; 7,53 (d, J=8,0 Hz, 1H).

Exemple D18 : Préparation de l'ester méthylique de la [(2*S*)-2-hydroxy-3-méthylsélénopropanoyl]-(*S*)-sélénométhionine (Composé 36b)

D18.1. Préparation de l'ester méthylique de la [(2*S*)-2-hydroxy-3-méthylsélénopropanoyl]-(*S*)-sélénométhionine (Composé 35b)

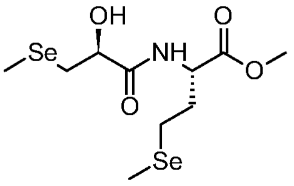

Composé 35b

Le composé **35b** est obtenu en utilisant les conditions de l'exemple D9 en partant de 177 mg (949 µmoles; 1 éq.) de l'acide (*S*)-2-hydroxy-3-(méthylséléno)propanoïque (**12**) et 260 mg (950 µmoles; 1 éq.) de l'ester méthylique du chlorhydrate de la (*S*)-sélénométhionine.

On obtient 236 mg (58 %) du produit désiré sous forme d'une huile jaune pâle.

^{1}H-RMN ($CDCl_3$, 400 MHz) : δ (ppm) = 2,03 (s, 3H) ; 2,04 (s, 3H) ; 2,12 (m, 1H) ; 2,29 (m, 1H) ; 2,57 (t, J=7,5 Hz, 2H) ; 2,92 (dd, J=13,0 Hz, J=8,0 Hz, 1H) ; 3,10 (dd, J=13,0 Hz, J=4,5 Hz, 1H) ; 3,79 (s, 3H) ; 4,22 (dd, J=8,0 Hz, J=4,5 Hz, 1H) ; 4,75 (m, 1H) ; 7,33 (d, J=8,5 Hz, 1H).

D18.2. Préparation de la [(2*S*)-2-hydroxy-3-méthylsélénopropanoyl]-(*S*)-sélénométhionine (Composé 36b)

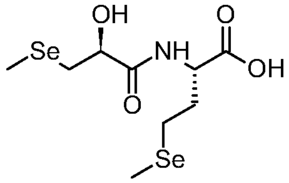

Composé 36b

Le composé **36b** est obtenu en utilisant les conditions de l'exemple B1 en partant de 186 mg du composé **35b**.

Après 16 h d'agitation à température ambiante, le milieu réactionnel est dilué dans l'eau (20 ml) puis extrait à l'acétate d'éthyle (2x10 mL). Le pH du milieu est ajusté à 1 par l'ajout d'une solution aqueuse d'acide chlorhydrique 2 M. Le milieu est extrait à l'acétate d'éthyle (3x10 mL). Les phases organiques sont regroupées, séchées sur Na_2SO_4, filtrées et concentrées. Le résidu est purifié sur colonne de silice (cyclohexane/acétate d'éthyle puis dichlorométhane/méthanol). L'huile obtenue est triturée avec du cyclohexane puis du pentane, solubilisée dans TBME et dichlorométhane puis concentré à sec.

On obtient 90 mg (50 %) du produit désiré sous forme d'une huile jaune.

^{1}H-RMN ($CDCl_3$, 400 MHz) : δ (ppm) = 2,05 (s, 6H) ; 2,19 (m, 1H) ; 2,34 (m, 1H) ; 2,63 (m, 2H) ; 2,91 (dd, J=13,5 Hz, J=8,0 Hz, 1H) ; 3,12 (dd, J=13,5 Hz, J=4,5 Hz, 1H) ; 4,26 (dd, J=8,0 Hz, J=4,5 Hz, 1H) ; 4,75 (m, 1H) ; 7,41 (d, J=8,0 Hz, 1H).

4- <u>Préparation des composés E : les acides 2-acyloxy-3-(alkylséléno)propanoique et esters correspondants</u>

Les composés E sont préparés soit en une étape par réaction des composés B avec des anhydrides carboxyliques, soit en deux étapes à partir de composés A par réactions avec des anhydrides carboxyliques suivi par hydrolyse.

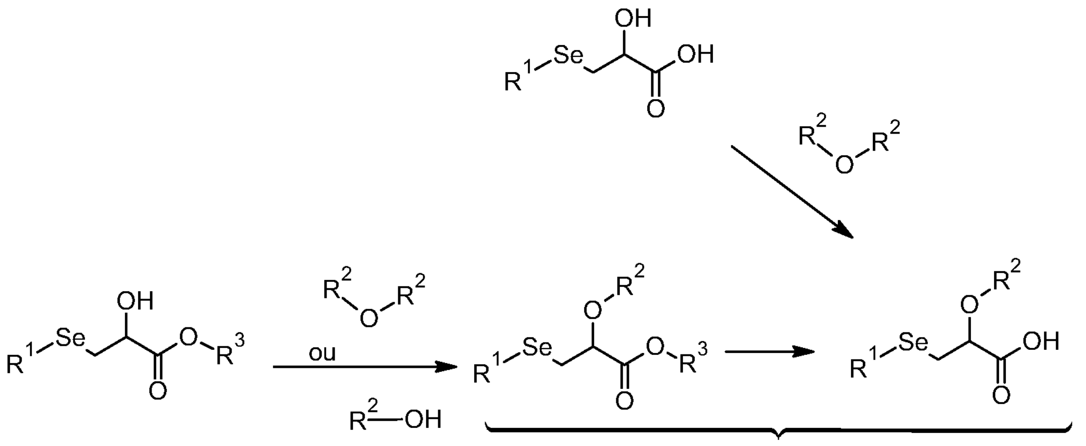

composés E

Exemple E1 : Préparation de l'acide 2-(acétyloxy)-3-(méthylséléno)propanoïque (Composé 37)

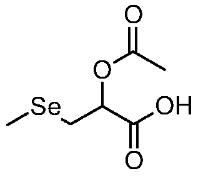

Composé 37

374 mg (2 mmoles) de l'acide 2-hydroxy-3-(méthylséléno)propanoïque (**10**) sont solubilisés sous azote dans 33 ml de dichlorométhane. 817 mg (756 µl; 8 mmoles; 4 éq.) d'anhydride acétique puis 2,5 mg (20 µmoles; 0,01 éq.) de 4-diméthylaminopyridine sont ajoutés au milieu. Le milieu est laissé sous agitation sous azote et à température ambiante pendant 6 h.

817 mg (756 µl; 8 mmoles; 4 éq.) d'anhydride acétique sont de nouveau ajoutés au milieu. Le milieu est laissé sous agitation sous azote et à température ambiante pendant 16 h.

10 mL d'eau sont additionnés puis le dichlorométhane du milieu est éliminé par évaporation. 40 mL d'une solution aqueuse de NH_4Cl saturée sont additionnés puis le milieu est extrait par acétate d'éthyle (3x40 mL). Les phases organiques sont regroupées, séchées avec Na_2SO_4, filtrées et concentrées. L'huile obtenue est purifiée sur colonne de silice (cyclohexane/acétate d'éthyle avec 1% TFA). On obtient 292 mg (63 %) du composé **37** sous forme d'une huile jaune.

^{1}H-RMN ($CDCl_3$, 400 MHz) : δ (ppm) = 2,15 (s, 3H) ; 2,20 (s, 3H) ; 2,97 (dd, J=13,5 Hz, J=7,5 Hz, 1H) ; 3,04 (dd, J=13,5 Hz, J=4,5 Hz, 1H) ; 5,37 (dd, J=7,5 Hz, J=4,5 Hz, 1H) ; 9,90 (s, 1H).

^{13}C-RMN ($CDCl_3$, 75 MHz) : δ (ppm) = 6,2; 21,0; 25,0; 72,6; 170,7

UPLC-MS (AP-) : 224,7 ($M-H^+$)

Exemple E2 : Préparation de l'acide 2-(dodécanoyloxy)-3-(méthylséléno)propanoïque (Composé 38)

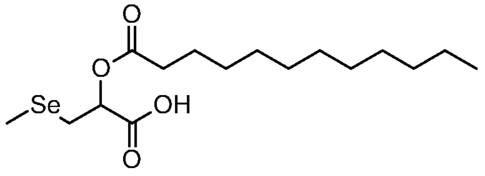

Composé 38

400 mg (2,14 mmoles) de l'acide 2-hydroxy-3-(méthylséléno)propanoïque (**10**) sont solubilisés sous azote dans 35 ml de dichlorométhane. 3,344 g (8,56 mmoles; 4 éq.) de l'anhydride laurique puis 2,6 mg (21 µmoles; 0,01 éq.) de 4-diméthylaminopyridine sont ajoutés au milieu. Le milieu est laissé sous agitation sous azote et à température ambiant pendant 6 h.

3,344 g (8,56 mmoles; 4 éq.) de l'anhydride laurique sont ajoutés de nouveau au milieu. Le milieu est laissé sous agitation sous azote et à température ambiant pendant 16 h.

10 mL d'eau sont additionnés puis le milieu est concentré à sec. Le résidu est purifié sur colonne de silice (cyclohexane/acétate d'éthyle puis dichlorométhane/méthanol). On obtient 394 mg (47 %) du composé **38** sous forme d'une huile jaune.

^{1}H-RMN ($CDCl_3$, 400 MHz) : δ (ppm) = 0,91 (m, 3H) ; 1,29 (m, 16H) ; 1,68 (m, 2H) ; 2,13 (s, 3H) ; 2,44 (t, J=7,5 Hz, 2H) ; 3,00 (m, 2H) ; 5,30 (m, 1H).

Exemple E3 : Préparation de l'acide 2-(benzoyloxy)-3-(méthylséléno)-propanoïque (Composé 40)

E3.1: Préparation de l'ester méthylique de l'acide 2-(benzoyloxy)-3-(méthylséléno)propanoïque (Composé 39)

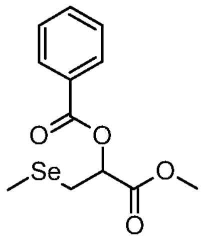

Composé 39

500 mg (2,46 mmoles) de composé **1** sont solubilisé sous azote dans 40 ml de dichlorométhane. 1,136 g (4,92 mmoles; 2 éq.) de l'anhydride benzoïque puis 30 mg (246 µmoles; 0,1 éq.) de 4-diméthylaminopyridine sont ajoutés au milieu. Le milieu est laissé sous agitation sous azote et à température ambiant pendant 25 h.

Le milieu est concentré à sec. Le résidu est purifié sur colonne de silice (cyclohexane/acétate d'éthyle). On obtient 633 mg (81 %) du composé **39** sous forme d'une huile incolore.

^{1}H-RMN ($CDCl_3$, 400 MHz) : δ (ppm) = 2,17 (s, 3H) ; 3,13 (m, 2H) ; 3,83 (s, 3H) ; 5,60 (t, J=6,0 Hz, 1H) ; 7,50 (t, J=7,5 Hz, 2H) ; 7,63 (t, J=7,5 Hz, 1H) ; 8,13 (d, J=7,5 Hz, 2H).

E3.2: Préparation de l'acide 2-(benzoyloxy)-3-(méthylséléno)propanoïque (Composé 40)

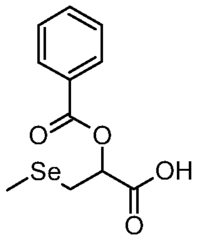

Composé 40

Le composé **40** est obtenu en utilisant les conditions de l'exemple B1 en partant du 372 mg du composé **39**.

Après 16 h d'agitation à température ambiante, le milieu réactionnel est dilué dans l'eau (10 ml) puis est extrait à l'acétate d'éthyle (2x10 mL). Le pH du milieu est ajusté à 1 par l'ajout d'une solution aqueuse d'acide chlorhydrique 2 M. Le milieu est extrait à l'acétate d'éthyle (3x10 mL). Les phases organiques de cette deuxième extraction sont regroupées, séchées sur Na_2SO_4, filtrées et concentrées.

On obtient 283 mg (81 %) du produit désiré sous forme d'un solide blanc.

^{1}H-RMN ($CDCl_3$, 400 MHz) : δ (ppm) = 2,13 (s, 3H) ; 2,99 (dd, J=13,5 Hz, J=6,0 Hz, 1H) ; 3,09 (dd, J=13,5 Hz, J=4,5 Hz, 1H) ; 4,58 (dd, J=6,0 Hz, J=4,5 Hz, 1H) ; 7,52 (t, J=7,5 Hz, 2H) ; 7,66 (t, J=7,5 Hz, 1H) ; 8,15 (d, J=7,5 Hz, 2H).

Exemple E4: Préparation de l'ester méthylique de l'acide 3-(méthylséléno)-2-[(3'-pyridine)oxycarbonyl]propanoïque (Composé 41)

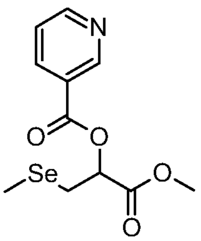

Composé 41

384 mg (1,85 mmoles) de composé **1** sont solubilisé sous azote dans 30 ml de dichlorométhane. 872 mg (3,7 mmoles; 2 éq.) de l'anhydride 3-pyridinecarboxylique puis 23 mg (185 µmoles; 0,1 éq.) de 4-diméthylaminopyridine sont ajoutés au milieu. Le milieu est laissé sous agitation sous azote et à température ambiant pendant 18 h. Le milieu est filtré sur verre fritté puis le filtrat est concentré à sec. Le résidu est purifié sur colonne de silice (cyclohexane/acétate d'éthyle).

On obtient 490 mg (83 %) du composé **41** sous forme d'une huile incolore.

^{1}H-RMN ($CDCl_3$, 400 MHz) : δ (ppm) = 2,16 (s, 3H) ; 3,13 (m, 2H) ; 3,84 (s, 3H) ; 5,60 (dd, J=7,0 Hz, J=5,0 Hz, 1H) ; 7,47 (dd, J=8,0 Hz, J=5,0 Hz, 1H) ; 8,39 (d, J=8,0 Hz, 1H) ; 8,85 (d, J=5,0 Hz, 1H) ; 9,31 (s, 1H).

Exemple E5: Préparation de l'acide 2-[(*tert*-butoxycarbonyl)oxy]-3-(méthylséléno)propanoïque (Composé 43)

E5.1 : Préparation de l'ester méthylique de l'acide 2-[(*tert*-butoxycarbonyl)oxy]-3-(méthylséléno)propanoïque (Composé 42)

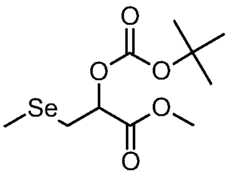

Composé 42

100 mg (507 µmoles) de composé **1** sont mis en solution dans 5 ml d'acétate d'éthyle. Le milieu est refroidi à 10°C puis 103 mg (143 µl; 1,01 mmoles; 2 éq.) de triéthylamine sont ajoutés goutte à goutte. 120 mg (533 µmoles; 1,05 éq.) de di-*tert*-butyl dicarbonate en solution dans 1 ml d'acétate d'éthyle sont ajoutés goutte à goutte rapidement. Le milieu est chauffé à 90°C pendant 48h.

Le milieu réactionnel est dilué avec de l'acétate d'éthyle (25 ml) puis le milieu est lavé avec une solution aqueuse d'acide citrique à 5 % (2x10 mL) puis avec une solution aqueuse saturée en NaCl (10 mL). La phase organique est séchée sur Na_2SO_4, filtrée et concentrée. Le résidu est purifié sur colonne de silice (cyclohexane/acétate d'éthyle).

On obtient 101 mg (63 %) du composé **42** sous forme d'une huile incolore.

^{1}H-RMN ($CDCl_3$, 400 MHz) : δ (ppm) = 1,55 (s, 9H) ; 2,13 (s, 3H) ; 2,94 (dd, J=13,5 Hz, J=7,5 Hz, 1H) ; 3,00 (dd, J=13,5 Hz, J=5,0 Hz, 1H) ; 3,82 (s, 3H) ; 5,17 (dd, J=7,5 Hz, J=5,0 Hz, 1H).

E5.2: Préparation de l'acide 2-[(*tert*-butoxycarbonyl)oxy]-3-(méthylséléno)propanoïque (Composé 43)

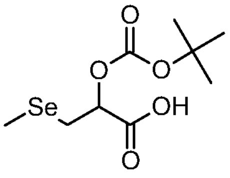

Composé 43

Le composé **43** est obtenu en utilisant les conditions de l'exemple B1 en partant de 363 mg du composé **42**.

Après 16 h d'agitation à température ambiante, le milieu réactionnel est dilué dans l'eau (25 ml) puis est extrait à l'acétate d'éthyle (2x20 mL). Le pH du milieu est ajusté à 4 par l'ajout d'une solution aqueuse d'acide citrique à 5 % (2x10 mL). Le milieu est extrait à l'acétate d'éthyle (5x25 mL). Les phases organiques de cette deuxième extraction sont regroupées, séchées sur Na_2SO_4, filtrées et concentrées.

Le résidu est purifié sur colonne de silice (dichlorométhane/méthanol) puis repurifié sur colonne de silice (cyclohexane/acétate d'éthyle).

On obtient 39 mg (11 %) du produit désiré sous forme d'une huile incolore.

^{1}H-RMN ($CDCl_3$, 400 MHz) : δ (ppm) = 1,52 (s, 9H) ; 2,14 (s, 3H) ; 2,96 (dd, J=13,5 Hz, J=7,5 Hz, 1H) ; 3,03 (dd, J=13,5 Hz, J=4,5 Hz, 1H) ; 5,20 (dd, J=7,5 Hz, J=4,5 Hz, 1H) ; 10,04 (s, 1H).

Exemple E6: Préparation de l'ester méthylique de l'acide (2*RS*)-[N-(*tert*-butoxycarbonyl)-*S*-methionyl]-3-méthylséléno-propanoïque (Composé 44)

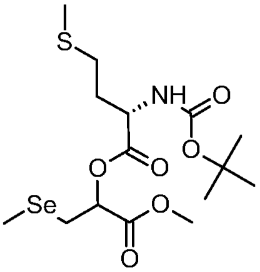

Composé 44

500 mg (2,0 mmoles) de la BOC-(*S*)-méthionine sont mis en solution dans 25 ml de dichlorométhane. 417 mg (2,0 mmoles; 1 éq.) de N,N'-dicyclohexylcarbodiimide, sont ajoutés. Le milieu est agité pendant 10 min à température ambiante puis 416 mg (2,0 mmoles; 1 éq.) de composé **1** et 25 mg (200 µmoles; 0,1 éq.) de 4-Diméthylaminopyridine sont ajoutés. Le milieu est agité à température ambiante pendant 16 h.

Le milieu est concentré à sec. Le résidu est purifié sur colonne de silice (cyclohexane/acétate d'éthyle).

On obtient 709 mg (78 %) du composé **44** sous forme d'une huile incolore.

^{1}H-RMN ($CDCl_3$, 400 MHz) mélange 50/50 de 2 diastéréoisomères **44a** (*R*,*S*) et **44b** (*S*,*S*) : δ (ppm) = 1,48 (s, 9H) ; 2,04 (m, 1H) ; 2,12 et 2,14 (2s, 3H) ; 2,15 et 2,16 (2s, 3H) ; 2,26 (m, 1H) ; 2,65 (m, 2H) ; 2,99 (m, 2H) ; 3,80 et 3,81 (2s, 3H) ; 4,58 (m, 1H) ; 5,17 (m, 1H) ; 5,39 (m, 1H).

UPLC-MS (AP+) : 451,9 $(M+Na)^+$

Exemple E7 : Préparation de l'ester méthylique de l'acide 4-méthylséléno-2-(2'-acétyloxy-3'-méthylsélénopropanoyl)butyrique (Composé 45) et de l'acide 4-méthylséléno-2-(2'-acétyloxy-3'-méthylsélénopropanoyl)butyrique (Composé 46)

E7.1. Préparation de l'ester méthylique de l'acide 4-méthylséléno-2-(2'-acétyloxy-3'-méthylsélénopropanoyl)butyrique (Composé 45)

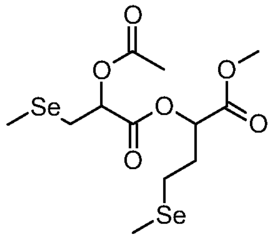

Composé 45

128 mg (522 µmoles) de l'acide 2-(acétyloxy)-3-(méthylséléno)propanoïque (**37**) sont solubilisé sous azote dans 5 ml de dichlorométhane. 108,7 mg (522 µmoles) de N,N'-Dicyclohexylcarbodiimide sont ajoutés au milieu. Le milieu est laissé sous agitation sous azote et à température ambiant pendant 10 min. 116 mg (522 µmoles) de méthyle 2-hydroxy-4-(méthylséléno)butanoate (*EP1778706*, préparé en analogie avec l'exemple 9 en utilisant méthanol au lieu d'éthanol) en solution dans 5 ml de dichlorométhane puis 6,4 mg (52 µmoles; 0,01 éq.) de 4-diméthylaminopyridine sont ajoutés. Le milieu est agité à température ambiante pendant 16 h.

Le milieu est concentré à sec. Le résidu est purifié sur colonne de silice (cyclohexane/acétate d'éthyle).

On obtient 189 mg (77 %) du composé **45** sous forme d'une huile incolore.

^{1}H-RMN ($CDCl_3$, 400 MHz) mélange de 2 diastéréoisomères : δ (ppm) = 2,03 et 2,04 (2s, 3H) ; 2,15 et 2,16 (2s, 3H) ; 2,20 (s, 3H) ; 2,23-2,3 (m, 2H) ; 2,54-2,7 (m, 2H) ; 2,95-3,02 (m, 1H) ; 3,11 (dd, J=13,5 Hz, J=4,0 Hz, 1H) ; 3,79 et 3,80 (2s, 3H) ; 5,15 à 5,45 (m, 2H).

UPLC-MS (AP+) : 442,9 $(M+Na)^+$

E7.2. Préparation de l'acide 4-méthylséléno-2-(2'-acétyloxy-3'-méthylsélénopropanoyl)butyrique (Composé 46) par hydrolyse de l'ester de méthyle

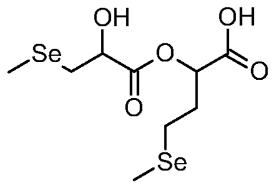

Composé 46

Le composé **46** est obtenu en utilisant les conditions de l'exemple B1 en partant de 180 mg du composé **45**.

Après 16 h d'agitation à température ambiante, le milieu réactionnel est dilué dans l'eau (20 ml) puis extrait à l'acétate d'éthyle (2x5 mL). La phase aqueuse est lyophilisée. Le lyophilisat est repris avec une solution à 4 N d'acide chlorhydrique dans le dioxane. Le milieu est agité 10 min puis concentré à sec. Le concentrât est repris dans 10 mL d'eau puis la solution est lyophilisée.

On obtient 138 mg (71 %) du produit désiré contenant 2 LiCl sous forme d'un solide collant jaune.

^{1}H-RMN (D_2O, 400 MHz) : δ (ppm) = 2,00 (s, 3H) ; 2,05 (s, 3H) ; 2,07-2,24 (m, 2H) ; 2,65 (m, 2H) ; 2,89 (dd, J=13,0 Hz, J=6,5 Hz, 1H) ; 3,00 (dd, J=13,0 Hz, J=4,5 Hz, 1H) ; 4,36 (dd, J=8,0 Hz, J=4,0 Hz, 1H) ; 4,50 (dd, J=6,5 Hz, J=4,5 Hz, 1H).

Exemple E8 : Préparation de l'acide 3-méthylséléno-2-(2'-hydroxy-4'-méthylsélénobutanoyl)propanoïque (Composé 48)

E8.1: Préparation de l'ester méthylique de l'acide 3-méthylséléno-2-(2'-acétyloxy-4'-méthylsélénobutanoyl)propanoïque (Composé 47)

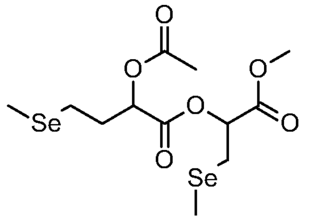

Composé 47

Le composé **47** est obtenu en utilisant les conditions de l'exemple E7 en partant de 89 mg de l'acide 2-acétyloxy-méthylsélénobutyrique (*EP1778706*, exemple 11) et 72 mg du composé **1**.

Le milieu est filtré sur verre fritté puis le filtrat est concentré à sec. Le résidu est purifié sur colonne de silice (cyclohexane/acétate d'éthyle).

On obtient 97 mg (60 %) du produit désiré sous forme d'une huile incolore.

^{1}H-RMN ($CDCl_3$, 400 MHz) mélange des diastéréoisomères : δ (ppm) = 2,05 et 2,06 (2s, 3H) ; 2,11 et 2,14 (2s, 3H) ; 2,18 et 2,19 (2s, 3H) ; 2,27-2,37 (m, 2H) ; 2,60-2,80 (m, 2H) ; 2,92-3,07 (m, 2H) ; 3,80 et 3,81 (2s, 3H) ; 5,24 (dd, J=12,5 Hz, J=6,0 Hz, 1H) ; 5,34 et 5,45 (2dd, J=8,0 Hz, J=4,5 Hz, 1H).

UPLC-MS (AP+) : 443,0 $(M+Na)^+$

E8.2 : Préparation de l'acide 3-méthylséléno-2-(2'-hydroxy-4'-méthylsélénobutanoyl)-propanoïque (Composé 48)

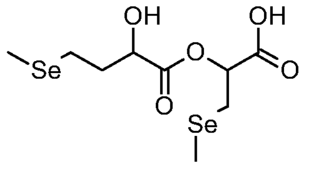

Composé 48

Le composé **48** est obtenu en utilisant les conditions de l'exemple **E7.2** en partant de 79 mg du composé **47**.

On obtient 69 mg (84 %) du produit désiré contenant 2 LiCl sous forme d'un solide collant incolore.

^{1}H-RMN (D_2O, 400 MHz) : δ (ppm) = 2,04 (m, 3H) ; 2,09-2,14 (m, 3H) ; 2,20-2,27 (m, 2H) ; 2,64-2,74 (m, 2H) ; 2,93 (dd, J=13,0 Hz, J=6,5 Hz, 1H) ; 3,04 (dd, J=13,5 Hz, J=4,5 Hz, 1H) ; 4,55 (dd, J=6,5 Hz, J=4,5 Hz, 1H), 5,05 (m, 1H).

Exemple E9 : Préparation de l'acide 2-(pentanoyloxy)-3-(méthylséléno)propanoïque (Composé 49)

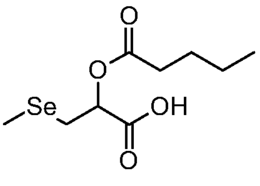

Composé 49

407 mg (1,98 mmoles) de l'acide 2-hydroxy-3-(méthylséléno)propanoïque (**10**) sont solubilisés sous azote dans 33 ml de dichlorométhane. 752 mg (817 µl; 3,96 mmoles; 2 éq.) d'anhydride valérique puis 2,4 mg (19,8 µmoles; 0,01 éq.) de 4-diméthylaminopyridine sont ajoutés au milieu. Le milieu est laissé sous agitation sous azote et à température ambiante pendant 24 h.

752 mg (817 µl; 3,96 mmoles; 2 éq.) d'anhydride valérique puis 2,4 mg (19,8 µmoles; 0,01 éq.) de 4-diméthylaminopyridine sont de nouveau ajoutés au milieu. Le milieu est laissé sous agitation sous azote et à température ambiante pendant 48 h.

10 mL d'eau sont additionnés puis le dichlorométhane du milieu est éliminé par évaporation. 25 mL d'une solution aqueuse de NH_4Cl saturée sont additionnés puis le milieu est extrait par acétate d'éthyle (3x10 mL). Les phases organiques sont regroupées, séchées avec Na_2SO_4, filtrées et concentrées. L'huile obtenue est purifiée sur colonne de silice (cyclohexane/acétate d'éthyle). On obtient 260 mg (48 %) du composé **49** sous forme d'une huile incolore.

^{1}H-RMN ($CDCl_3$, 400 MHz) : δ (ppm) = 0,96 (t, J=7,4 Hz, 3H) ; 1,42 (m, 2H) ; 1,69 (m, 2H) ; 2,15 (s, 3H) ; 2,46 (td, J=1,9 Hz, J=7,4 Hz, 2H) ; 3,01 (m, 2H) ; 5,36 (dd, J=4,3 Hz, J=7,6 Hz, 1H).

UPLC-MS (AP-) : 267,3 (M-H$^+$)

Exemple E10 : Préparation de l'acide 2-(nonanoyloxy)-3-(méthylséléno)propanoïque (Composé 50)

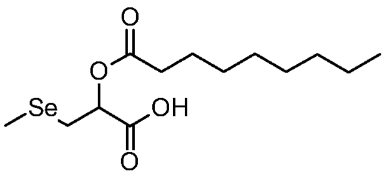

Composé 50

260 mg (1,39 mmoles) de l'acide 2-hydroxy-3-(méthylséléno)propanoïque (**10**) sont solubilisés sous azote dans 23 ml de dichlorométhane. 874 mg (963 µl; 2,78 mmoles; 2 éq.) d'anhydride nonanoique puis 1,7 mg (13,9 µmoles; 0,01 éq.) de 4-diméthylaminopyridine sont ajoutés au milieu. Le milieu est laissé sous agitation sous azote et à température ambiante pendant 24 h.

874 mg (963 µl; 2,78 mmoles; 2 éq.) d'anhydride nonanoique puis 1,7 mg (13,9 µmoles; 0,01 éq.) de 4-diméthylaminopyridine sont ajoutés au milieu. Le milieu est laissé sous agitation sous azote et à température ambiante pendant 24 h.

10 mL d'eau sont additionnés puis le dichlorométhane du milieu est éliminé par évaporation. 25 mL d'une solution aqueuse de NH_4Cl saturée sont additionnés puis le milieu est extrait par acétate d'éthyle (3x10 mL). Les phases organiques sont regroupées, séchées avec Na_2SO_4, filtrées et concentrées. L'huile obtenue est purifiée sur colonne de silice (cyclohexane/acétate d'éthyle). On obtient 116 mg (25 %) du composé **50** sous forme d'une huile incolore.

^{1}H-RMN ($CDCl_3$, 400 MHz) : δ (ppm) = 0,91 (t, J=6,9 Hz, 3H) ; 1,34 (m, 10H) ; 1,70 (m, 2H) ; 2,14 (s, 3H) ; 2,45 (td, J=1,7 Hz, J=7,4 Hz, 2H) ; 3,01 (m, 2H) ; 5,37 (dd, J=4,3 Hz, J=7,6 Hz, 1H).

UPLC-MS (AP-) : 322,9 (M-H$^+$)

Exemple E11.1. : Préparation de l'ester méthylique de l'acide 2-(linoleoyloxy)-3-(méthylséléno)propanoïque (Composé 51)

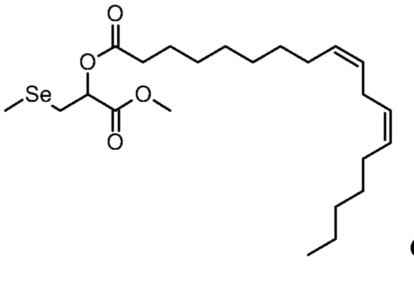

Composé 51

818 mg (2,83 mmoles) d'acide linoleique sont mis en solution dans 35 mL de dichlorométhane. 589 mg (2,83 mmoles; 1 éq.) de N,N'-dicyclohexylcarbodiimide, sont ajoutés. Le milieu est agité pendant 10 min à température ambiante puis 575 mg (2,83 mmoles; 1 éq.) de composé **1** et 35 mg (283 µmoles; 0,1 éq.) de 4-diméthylaminopyridine sont ajoutés. Le milieu est agité à température ambiante pendant 48 h.

Le milieu est filtré sur verre fritté puis le filtrat est concentré à sec. Le résidu est purifié sur colonne de silice (cyclohexane/acétate d'éthyle). On obtient 801 mg (60%) du composé **51** sous forme d'une huile incolore.

^{1}H-RMN ($CDCl_3$, 400 MHz) : δ (ppm) = 0,92 (t, J=6,9 Hz, 3H) ; 1,34 (m, 14H) ; 1,70 (m, 2H) ; 2,08 (m, 4H) ; 2,12 (s, 3H) ; 2,45 (td, J=2,8 Hz, J=7,4 Hz, 2H) ; 2,8 (t, J=6,6 Hz, 2H) ; 2,97 (m, 2H) ; 3,8 (s, 3H) ; 5,38 (m, 5H).
UPLC-MS (AP+) : 461,2 ($M+H^+$)

Exemple E11.2 : Préparation de l'acide 2-(linoleoyloxy)-3-(méthylséléno)propanoïque (Composé 52)

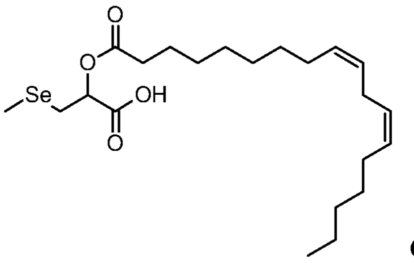

Composé 52

459 mg (1,59 mmoles) d'acide linoleique sont mis en solution dans 19,8 mL de dichlorométhane. 331 mg (2,83 mmoles; 1 éq.) de N,N'-dicyclohexylcarbodiimide, sont ajoutés. Le milieu est agité pendant 30 min à température ambiante puis 300 mg (1,59 mmoles; 1 éq.) d'acide 2-hydroxy-3-(méthylséléno)propanoïque et 20 mg (159 µmoles; 0,1 éq.) de 4-Diméthylaminopyridine sont ajoutés. Le milieu est agité à température ambiante pendant 24 h.
Le milieu est filtré sur verre fritté puis le filtrat est concentré à sec. Le résidu est purifié sur colonne de silice (cyclohexane/acétate d'éthyle puis dichlorométhane/méthanol). On obtient 198 mg (26 %) du composé **52** sous forme d'une huile incolore.

^{1}H-RMN ($CDCl_3$, 300 MHz) : δ (ppm) = 0,93 (t, J=6,8 Hz, 3H) ; 1,35 (m, 14H) ; 1,70 (m, 2H) ; 2,1 (m, 4H) ; 2,15 (s, 3H) ; 2,45 (m, 2H) ; 2,8 (t, J=5,9 Hz, 2H) ; 3,0 (m, 2H) ; 5,4 (m, 5H).
UPLC-MS (AP-) : 444,7 ($M-H^+$)

Exemple E12 : Préparation de l'ester méthylique de l'acide 2-[(1H-imidazol-4-ylcarbonyl)oxy]-3-(méthylséléno)propanoïque (Composé 53)

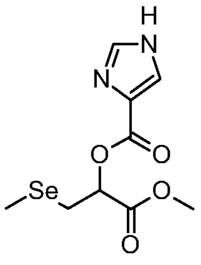

Composé 53

438 mg (3,83 mmoles) d'acide 4-imidazolecarboxylique sont mis en suspension dans 20 mL de dichlorométhane et 30 µl de N,N-diméthylformamide (383 µmoles; 0,1 éq.). Le milieu est refroidie à 0°C puis 503 µL (5,74 mmoles; 1,5 éq.) de chlorure d'oxalyle sont ajoutés. Le milieu est refroidi à 0°C pendant 15 min puis agité 2 h à température ambiante. Le milieu est concentré à sec.

Le concentrât est repris dans 20 ml de dichlorométhane. Le milieu est refroidie à 0°C puis 1,26 mL (7,66 mmoles; 2 éq.) de N,N-diisopropylethylamine sont ajoutés. 794 mg (3,83 mmoles; 1 éq.) de composé **1** sont ajoutés goutte à goutte. Le milieu est agité à température ambiante pendant 48 h.

Le milieu est dilué avec 50 mL d'acétate d'éthyle puis lavé avec 20 mL d'eau. La phase aqueuse est extraite avec 20 mL d' acétate d'éthyle. Les phases organiques sont regroupées, lavées avec 20 mL d'une solution aqueuse de NaCl saturée. La phase organique est séchée avec Na_2SO_4, filtrée et concentrée. Le résidu est purifié sur colonne de silice (cyclohexane/acétate d'éthyl puis dichlorométhane/méthanol). On obtient 806 mg (70 %) du composé **53** sous forme d'un solide jaune pâle.

^{1}H-RMN (DMSO, 400 MHz) : δ (ppm) = 2,12 (s, 3H) ; 3,03 (m, 2H) ; 3,70 (s, 3H) ; 5,42 (dd, J=4,6 Hz, J=7,3 Hz, 1H) ; 7,84 (s, 1H) ; 7,88 (s, 1H) ; 12,74 (sl, 1H).

UPLC-MS (AP+) : 314,7 (M+Na$^+$)

Exemple E13.1 : Préparation de l'ester méthylique de l'acide 2-(pivaloyloxy)-3-(méthylséléno)propanoïque (Composé 54)

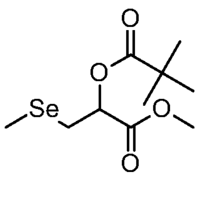

Composé 54

400 mg (1,99 mmoles) de composé **1** sont solubilisés sous azote dans 50 mL de dichlorométhane. 1,5 g (7,95 mmoles; 4 éq.) d'anhydride triméthylacétique puis 24 mg (199 µmoles; 0,1 éq.) de 4-diméthylaminopyridine sont ajoutés au milieu. Le milieu est laissé sous agitation sous azote et à température ambiante pendant 48 h.
748 mg (3,98 mmoles; 2 éq.) d'anhydride triméthylacetique sont de nouveau ajoutés au milieu. Le milieu est laissé sous agitation sous azote et à température ambiante pendant 96 h.
Le milieu est concentré à sec. Le résidu est purifié sur colonne de silice (cyclohexane/acétate d'éthyle). On obtient 484 mg (83 %) du composé **54** sous forme d'une huile incolore.

^{1}H-RMN ($CDCl_3$, 400 MHz) : δ (ppm) = 1,29 (s, 9H) ; 2,13 (s, 3H) ; 2,99 (m, 2H) ; 3,79 (s, 3H) ; 5,31 (dd, J=4,6 Hz, J=7,5 Hz, 1H).
UPLC-MS (AP+) : 281,9 ($M+H^+$)

Exemple E13.2 : Préparation de l'acide 2-(pivaloyloxy)-3-(méthylséléno)propanoïque (Composé 55)

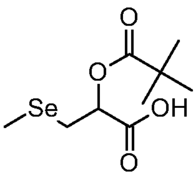

Composé 55

240 mg (0,83 mmoles) de composé **54** sont solubilisés dans 12 mL de THF. 830 µl (0,83 mmoles; 1 équiv.) d'une solution aqueuse d'hydroxyde de lithium à 1M sont ajoutés, la solution est agitée à température ambiante pendant 16 h.
41 µL (41µmoles; 0,05 équiv.) d'une solution aqueuse d'hydroxyde de lithium à 1M sont ajoutés, la solution est agitée à température ambiante pendant 24 h.
Le milieu réactionnel est dilué dans l'eau (20 mL) puis est extrait à l'acétate d'éthyle (3x20 mL). Le pH du milieu est ajusté à 4 par l'ajout d'une solution aqueuse d'acide chlorhydrique à 1 M. Le milieu est extrait à l'acétate d'éthyle (3x20 mL). Les phases organiques de cette deuxième extraction sont regroupées, séchées sur Na_2SO_4, filtrées et concentrées. On obtient 151 mg (66 %) du produit désiré **55** sous forme d'une huile incolore.

^{1}H-RMN ($CDCl_3$, 400 MHz) : δ (ppm) = 1,29 (s, 9H) ; 2,15 (s, 3H) ; 3,03 (m, 2H) ; 5,32 (dd, J=7,7 Hz, J=4,3 Hz, 1H).

UPLC-MS (AP-) : 266,6 ($M-H^+$)

Exemple E14 : Préparation de l'ester méthylique de l'acide 2-(3-chloropropanoyloxy)-3-(méthylséléno)propanoïque (Composé 56)

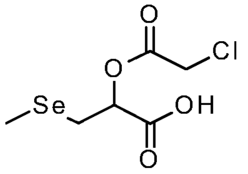

Composé 56

400 mg (1,99 mmoles) de composé **1** sont mis en suspension sous azote dans 6 mL d'acétone. 324 mg (3,98 mmoles; 2 éq.) de pyridine sont ajoutés au milieu. Le milieu est refroidie à - 10°C puis 390 µl (3,98 mmoles; 2 éq.) de 3-chloropropionyl chloride sont ajoutés au milieu à - 10°C. Le milieu est laissé sous agitation et à température ambiante pendant 64 h.

Le milieu réactionnel est dilué dans l'eau (20 mL) puis est extrait à l'acétate d'éthyle (2x20 mL). Les phases organiques sont regroupées, lavées avec 2x20 mL d'une solution aqueuse de $NaHCO_3$ à 1M et 10 mL d'une solution aqueuse de NaCl saturée. La phase organique est séchée avec Na_2SO_4, filtrée et concentrée. Le résidu est purifié sur colonne de silice (cyclohexane/acétate d'éthyl). On obtient 301 mg (51 %) du composé **56** sous forme d'une huile incolore.

^{1}H-RMN ($CDCl_3$, 400 MHz) : δ (ppm) = 2,13 (s, 3H) ; 2,97 (m, 4H) ; 3,81 (s, 3H) ; 3,82 (t, J=6,6 Hz, 2H) ; 5,4 (dd, J=4,6 Hz, J=7,5 Hz, 1H).

Tableau récapitulatif des exemples de préparation des nouveaux composés

Ex.	Composé	Structure	R1	R2	X
**A1**	**1**	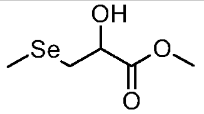	CH_3	H	OCH_3
**A2**	**1**	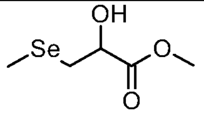	CH_3	H	OCH_3

**A3**	**2**	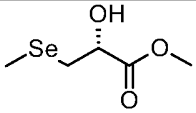	CH_3	H	OCH_3
**A4**	**3**	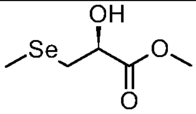	CH_3	H	OCH_3
**A5**	**4**	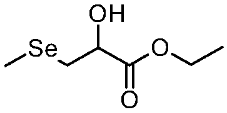	CH_3	H	OCH_2CH_3
**A6**	**5**	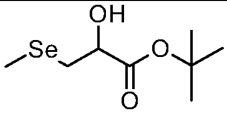	CH_3	H	$OC(CH_3)_3$
**A7**	**6**	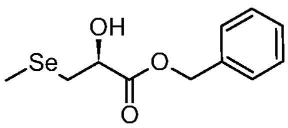	CH_3	H	$OCH_2C_6H_5$
**A8**	**7**	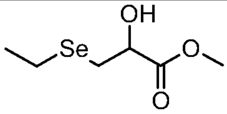	CH_2CH_3	H	OCH_3
**A9**	**8**	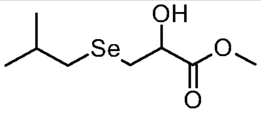	$CH_2CH(CH_3)_2$	H	OCH_3
**A10**	**4**	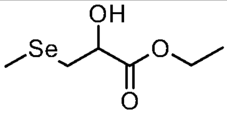	CH_3	H	OCH_2CH_3
**A11**	**9**	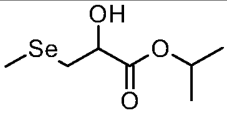	CH_3	H	$OCH(CH_3)_2$
**B1**	**10**	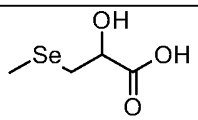	CH_3	H	OH
**B2**	**10**	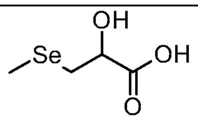	CH_3	H	OH
**B3**	**11**	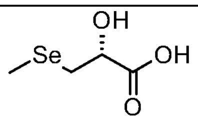	CH_3	H	OH
**B4**	**12**	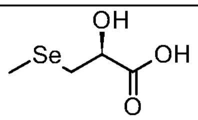	CH_3	H	OH
**B5**	**12**	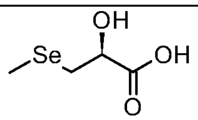	CH_3	H	OH

**B6**	**13**	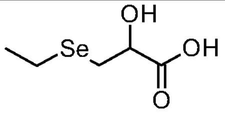	CH_2CH_3	H	OH
**B7**	**14**	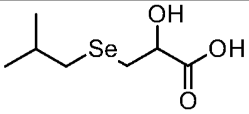	$CH_2CH(CH_3)_2$	H	OH
**C1**	**15**	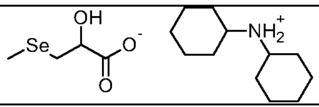	CH_3	H	$ONH_2(C_6H_{11})_2$
**C2**	**16**	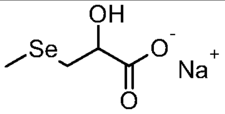	CH_3	H	Sel de Na
**C3**	**17**	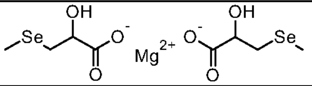	CH_3	H	Sel de Mg
**C4**	**18**	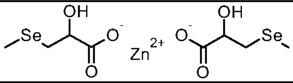	CH_3	H	Sel de Zn
**C5**	**19**	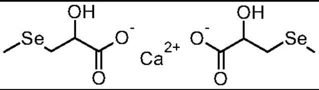	CH_3	H	Sel de Ca
**D1**	**20**	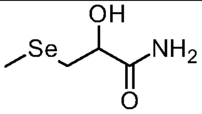	CH_3	H	NH_2
**D2**	**21**	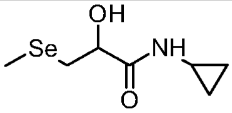	CH_3	H	NHC_3H_5
**D3**	**22**	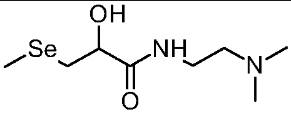	CH_3	H	$NH(CH_2)_2N(CH_3)_2$
**D4**	**23**	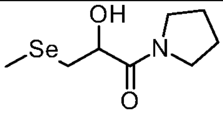	CH_3	H	$N(CH_2)_4$
**D5**	**24**	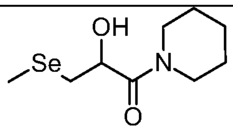	CH_3	H	$N(CH_2)_5$
**D6**	**25**	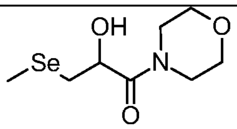	CH_3	H	$N(CH_2)_4O$
**D7**	**26**	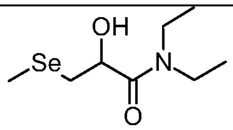	CH_3	H	$N(C_2H_5)_2$
**D8**	**27**	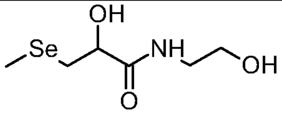	CH_3	H	$NH(CH_2)_2OH$

**D9.1**	**28**	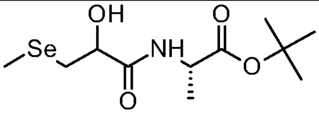	CH_3	H	$NHAlaOC(CH_3)_3$
**D9.2**	**29**	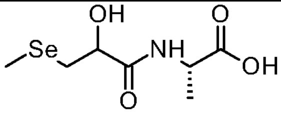	CH_3	H	NHAlaOH
**D10.1**	**30**	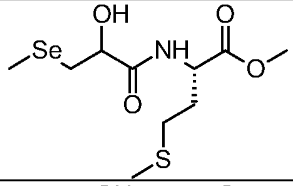	CH_3	H	$NHMetOCH_3$
**D10.2**	**31**	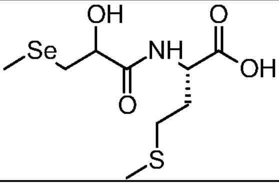	CH_3	H	NHMetOH
**D11.1**	**30a**	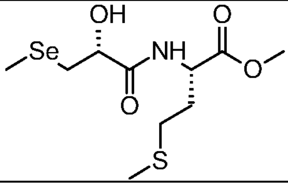	CH_3	H	$NHMetOCH_3$
**D11.2**	**31a**	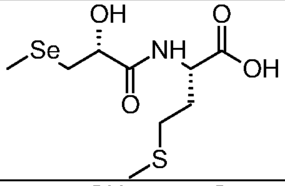	CH_3	H	NHMetOH
**D12.1**	**30b**	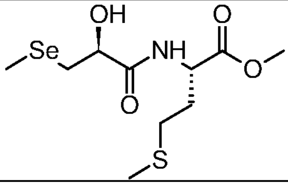	CH_3	H	$NHMetOCH_3$
**D12.2**	**31b**	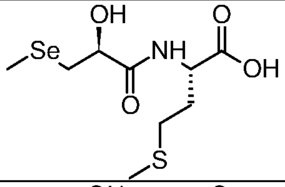	CH_3	H	NHMetOH
**D13**	**32**	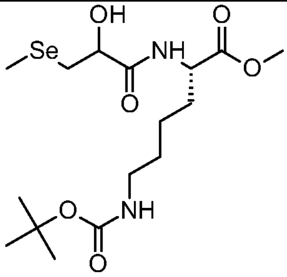	CH_3	H	NHLys(NHBoc)O CH_3

**D14**	**33**	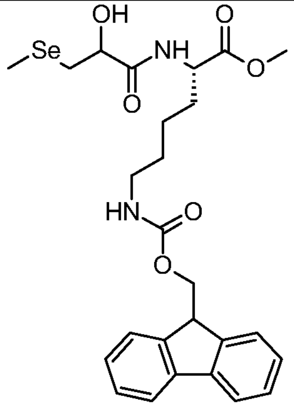	CH_3	H	NHLys(NHFmoc)OCH3
**D15**	**34**	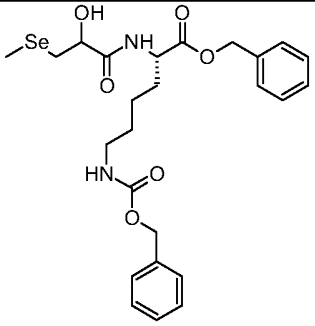	CH_3	H	NHLys(NHCbz)OCH2C6H5
**D16.1**	**35**	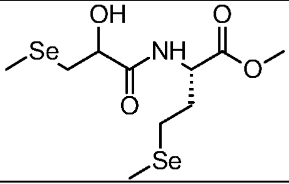	CH_3	H	NHSeMetOCH3
**D16.2**	**36**	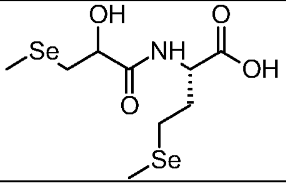	CH_3	H	NHSeMetOH
**D17.1**	**35a**	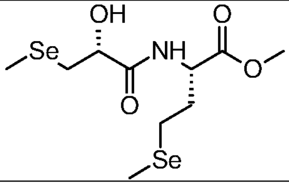	CH_3	H	NHSeMetOCH3
**D17.2**	**36a**	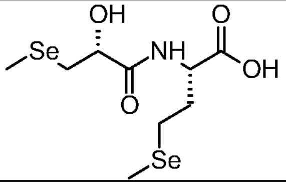	CH_3	H	NHSeMetOH
**D18.1**	**35b**	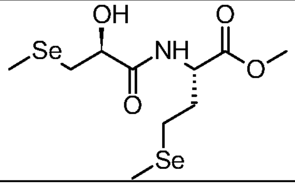	CH_3	H	NHSeMetOCH3

**D18.2**	**36b**	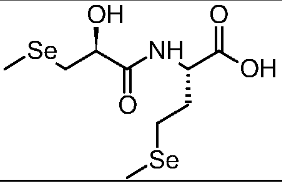	CH_3	H	NHSeMetOH
**E1**	**37**	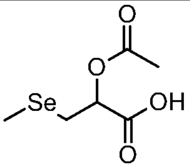	CH_3	$C(=O)CH_3$	OH
**E2**	**38**	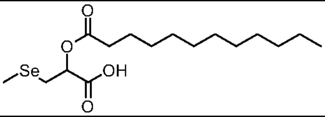	CH_3	$C(=O)(CH_2)_{10}$-CH_3	OH
**E3.1**	**39**	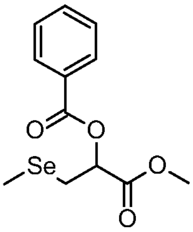	CH_3	$C(=O)C_6H_5$	OCH_3
**E3.2**	**40**	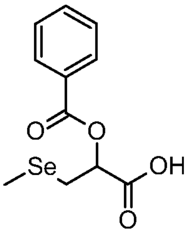	CH_3	$C(=O)C_6H_5$	OH
**E4**	**41**	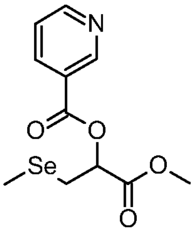	CH_3	$C(=O)C_5H_4N$	OCH_3
**E5.1**	**42**	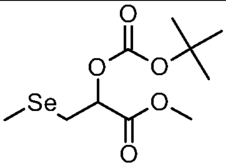	CH_3	$C(=O)OC(CH_3)_3$	OCH_3
**E5.2**	**43**	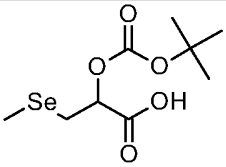	CH_3	$C(=O)OC(CH_3)_3$	OH

**E6**	**44**	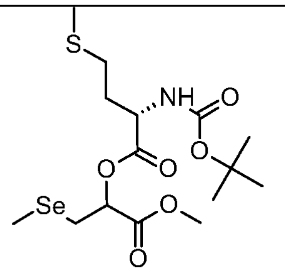	CH_3	MetNHBoc	OCH_3
**E7.1**	**45**	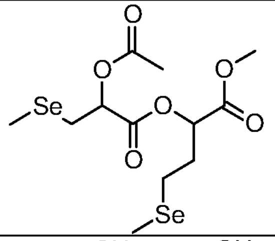	CH_3	$C(=O)CH_3$	$OSeMetOCH_3$
**E7.2**	**46**	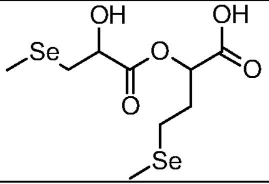	CH_3	H	OSeMetOH
**E8.1**	**47**	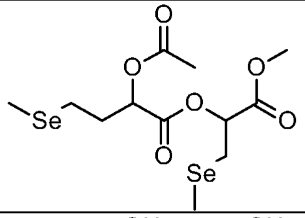	CH_3	$C(=O)CH_3$	$OSeMetOCH_3$
**E8.2**	**48**	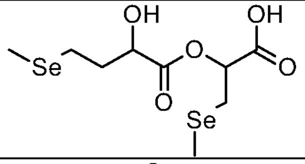	CH_3	H	OSeMetOH
**E9**	**49**	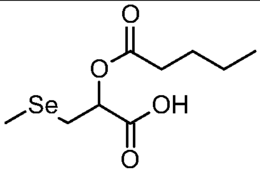	CH_3	$C(=O)C_4H_9$	OH
**E10**	**50**	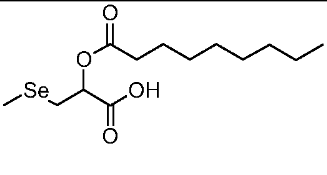	CH_3	$C(=O)C_4H_9$	OH
**E11.1**	**51**	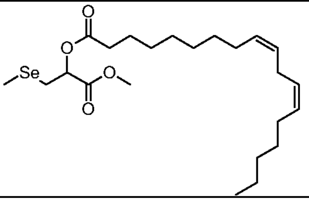	CH_3	$C(=O)C_{17}H_{31}$	OCH_3

**E11.2**	**52**	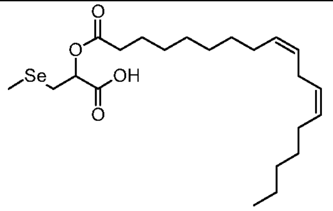	CH_3	$C(=O)C_{17}H_{31}$	OH
**E12**	**53**	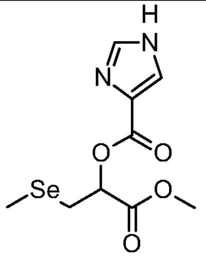	CH_3	$C(=O)C_3H_3N_2$	OCH_3
**E13.1**	**54**	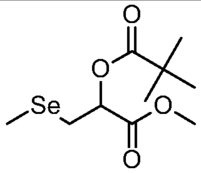	CH_3	$C(=O)C(CH_3)_3$	OCH_3
**E13.2**	**55**	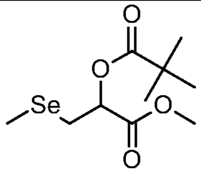	CH_3	$C(=O)C(CH_3)_3$	OH
**E14**	**56**	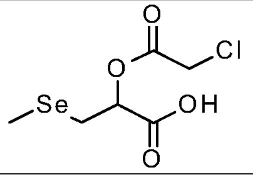	CH_3	$C(=O)(CH_2)_2Cl$	OCH_3

II. Exemples décrivant l'activité anti-tumorale des composés selon l'invention

II.1. Lignées cellulaires

Huit lignées cellulaires issues de différents types de cancers ont été utilsées: PC3 et DU145 (prostate), HT-29 et LS-174T (colon), Hep G2 (foie), MCF-7 (sein), MIA PaCa-2 et PANC-1 (pancréas).

Les caractéristiques de chaque lignée cellulaire sont résumées dans le **Tableau I.**

Tableau I :Caractéristiques des lignées cellulaires utilisées

Organe	Lignée cellulaire	Description
Prostate	PC3	Adhérente, adénocarcinome prostate humain grade IV dérivée de métastase osseuse
	DU145	Adhérente, carcinome prostate humain
Colon	HT-29	Adhérente, adénocarcinome humain (homme 44 ans Caucasien)
	LS174T	Adhérente, adénocarcinome humain (femme 58 ans Caucasien)
Liver	Hep G2	Adhérente, carcinome hépatocellulaire (homme 15 ans Caucasien)
Breast	MCF-7	Adhérente, adénocarcinome humain (femme 69 ans Caucasien)
Pancreas	PANC-1	Adhérente, carcinome (homme 56 ans Caucasien)
	MIA PaCa-2	Adhérente, carcinome (homme 65 ans Caucasien)

II.2. Milieux de culture

Les cellules sont cultivées dans le milieu de culture spécifique décrit dans le **Tableau II**, à 37°C, 5% CO_2, selon les procédures opératoires bien connues de l'Homme de l'Art.

Tableau II: Composition des milieux de culture

Organe	Lignée cellulaire	Milieu de Culture
Prostate	PC3	RPMI 1640 + 10% FBS
	DU145	RPMI 1640 + 10% FBS
Colon	HT-29	Mc Coy's 5a + 10% FBS + 0,5 mM Ultraglutamine
	LS174T	EMEM + 10% FBS + 2 mM Ultraglutamine + 1 mM pyruvate de sodium + 0,1 mM amino-acides non essentiels
Sein	MCF-7	EMEM + 10% FBS + 2 mM Ultraglutamine + 1 mM pyruvate de sodium + 0,1 mM amino-acides non essentiels + 10 nM β-estradiol
Pancréas	PANC-1	RPMI + 10% FBS
	MIA PaCa-2	DMEM + 10% FBS
Foie	Hep G2	EMEM + 10% de FBS complémenté + 0,1 mM amino-acides non essentiels + 2 mM ultraglutamine + Penicilline/Streptomycine

II.3. Evaluation de la cytotoxicité des composés selon l'invention

Après décongélation et amplification des cellules cancéreuses dans le milieu de culture approprié (décrit en 1.2.), des plaques 96 puits sont ensemencées avec ces cellules et incubées ou non (contrôles), dans leur milieu de culture respectif, en présence des composés à tester à 10, 50, 100, 250 et 500 µM.

Après 96h d'incubation, chaque plaque 96-puits est analysée afin de mesurer la viabilité des cellules par un test colorimétrique au WST-1.

II.4 Exemples

F1. Activité anti-tumorale du composé 4 (voir exemple A5)

Les résultats de cytotoxicité obtenus avec le composé 4 sur deux lignées cellulaires sont présentés sur la Figure 2. Ces résultats montrent que la viabilité des cellules diminue d'autant plus que la concentration en composé 4 augmente, la concentration en composé 4 qui diminue de 50% la viabilité cellulaire étant égale à 481 et 335 µM, respectivement pour les cellules DU145 et LS174T.

F2. Activité anti-tumorale du composé 10 (voir exemple B1)

Les résultats de cytotoxicité obtenus avec le composé 10 sur trois lignées cellulaires sont présentés sur la Figure 3. Ces résultats montrent que la viabilité des cellules diminue d'autant plus que la concentration en composé 10 augmente, la concentration en composé 10 qui diminue de 50% la viabilité cellulaire étant égale à 392, 430 et 328 µM, respectivement pour les cellules DU145, LS174T et HT-29.

F3. Activité anti-tumorale du composé 38 (voir exemple E2)

Les résultats de cytotoxicité obtenus avec le composé 38 sur huit lignées cellulaires sont présentés sur les Figures 4a et 4b. Ces résultats montrent que la viabilité des cellules diminue d'autant plus que la concentration en composé 38 augmente. La concentration en composé 38 qui diminue de 50% la viabilité cellulaire est donnée dans le tableau III pour chaque lignée cellulaire.

Tableau III : Concentration en composé 38 diminuant de 50% la viabilité cellulaire après 96 heures de traitement

Lignée cellulaire	Composé 38 (µM)
PC3	198
DU145	169
PANC-1	253
MIA PaCa-2	308
HT-29	166
LS174T	113
Hep G2	263
MCF-7	215

III. Exemples décrivant les compositions des composés selon l'invention

Exemple G1 : Compositions contenant le composé 10

On a préparé des gélules de composition suivante :

Composé 10 Acide 2-hydroxy-3-méthylsélénopropanoïque 0,40mg (en eq. Se)

Excipients* et enveloppe** une gélule de 1000 mg

(* amidon de maïs, lactose, stéarate de magnésium, arôme,

** gélatine, dioxyde de titane, colorants)

Exemple G2 : Compositions contenant le composé 38

On a préparé des gélules de composition suivante :

Composé 38 Acide 2-(dodécanoyloxy)-3-(méthylséléno) propanoïque 0,40mg (en eq. Se)

Excipients* et enveloppe** une gélule de 1000 mg

(* amidon de maïs, lactose, stéarate de magnésium, arôme,

** gélatine, dioxyde de titane, colorants)

Exemple G3 : Compositions contenant le composé 10 et le composé 38

On a préparé des gélules de composition suivante :

Composé 10 Acide 2-hydroxy-3-méthylsélénopropanoïque 0,10mg (en eq. Se)

Composé 38 Acide 2-(dodécanoyloxy)-3-(méthylséléno) propanoïque 0,40mg (en eq. Se)

Excipients* et enveloppe** une gélule de 1000 mg

(* amidon de maïs, lactose, stéarate de magnésium, arôme,

** gélatine, dioxyde de titane, colorants)

REVENDICATIONS

1. Composé sélénié répondant à la formule générale (I) suivante :

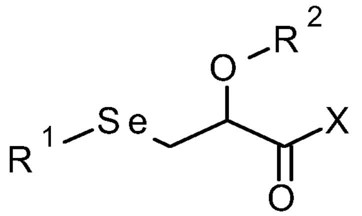

FORMULE GÉNÉRALE (I)

dans laquelle

$\mathbf{R^1}$ = alkyle ;

$\mathbf{R^2}$ = H, $\mathbf{R^4}$C(=O), $\mathbf{R^4}$OC(=O), α-amino-acyle, $CH_3SeCH_2CH_2CH(NH_2)C(=O)$, $CH_3SeCH_2CH_2CH(OH)C(=O)$;

X = OH, O$\mathbf{R^3}$, NH_2, N$\mathbf{R^4R^5}$, α-amino-acide, $CH_3SeCH_2CH_2CH(COOH)NH$- , $CH_3SeCH_2CH_2CH(COOH)O$-;

$\mathbf{R^3}$ = alkyle ;

$\mathbf{R^4}$ = alkyle, aryle ;

$\mathbf{R^5}$ = H, alkyle, aryle ;

$\mathbf{R^4}$ et $\mathbf{R^5}$ pouvant former ensemble un radical cyclo-alkyle à 5 ou 6 chainons pouvant comporter un hétéroatome;

étant entendu que lorsque **X** = NHterbutyle alors $\mathbf{R^2} \neq C(=O)CH_3$

ainsi que tous les stéréoisomères, diastéréoisomères et énantiomères notamment au niveau de l'atome de carbone portant le groupement O$\mathbf{R^2}$, ainsi qu'au niveau des radicaux $\mathbf{R^1}$ à $\mathbf{R^5}$, ainsi que tous oligomères (dimères, trimères, ...) et polymères, linéaires ou ramifiés, acycliques ou cycliques, obtenus entre deux ou plusieurs molécules de dérivés de formule (I) décrits dans l'invention par réaction d'estérification entre les fonctions alcool et acide carboxylique présentes le cas échéant, pris isolément ou en mélange ;

ainsi que tous les sels d'acides ou de bases pharmaceutiquement acceptables desdits composés de formule générale (I) ;

ainsi que les sels de sodium, de calcium, de zinc et de magnésium.

2. Composé sélénié selon la revendication 1, caractérisé en ce que R^1 représente un groupe méthyle, éthyle, allyle.

3. Composé sélénié selon la revendication 1 ou 2, caractérisé en ce que R^2 est choisi dans le groupe constitué par H, α-amino-acyles, $R^4(C{=}O)$, $R^4O(C{=}O)$, $CH_3SeCH_2CH_2CH(OH)C({=}O)$.

4. Composé sélénié selon l'une des revendications 1 à 3, caractérisé en ce que X est choisi dans le groupe OH, α-amino-acide, $CH_3SeCH_2CH_2CH(COOH)NH$-, $CH_3SeCH_2CH_2CH(COOH)O$-.

5. Composé sélénié selon l'une des revendications 1 à 4, caractérisé en ce que R^1 représente un groupe méthyle, éthyle, allyle ; R^2 représente $R^4(C{=}O)$, $R^4O(C{=}O)$ et X représente OH ou OR^3.

6. Composé sélénié selon l'une des revendications 1 à 5, caractérisé en ce que les acides pharmaceutiquement acceptables, sont choisis parmi les acides minéraux tels que chlorhydrique, bromhydrique, iodhydrique, sulfurique, tartrique, phosphorique ; ou choisis parmi les acides organiques tels que les acides formique, acétique, trifluoro-acétique, propionique, benzoïque, maléique, fumarique, succinique, citrique, oxalique, glyoxylique, aspartique, alcanesulfoniques tels que les acides méthane-sulfonique, trifluorométhane-sulfonique, éthane-sulfonique, aryl-sulfoniques tels que les acides benzène- et paratoluène-sulfonique.

7. Composé sélénié selon l'une des revendications 1 à 5, caractérisé en ce que les bases pharmaceutiquement acceptables sont choisies parmi les bases minérales telles que les hydroxydes de sodium, de lithium, de calcium, de potassium, de magnésium, d'ammonium ou de zinc, les carbonates de métaux alcalins ou alcalino-terreux tels que les carbonates et bicarbonates de sodium, de lithium, de calcium, de potassium, de magnésium, d'ammonium ou de zinc ou des bases organiques comme la méthylamine, la propylamine, la triméthylamine, la diéthylamine, la triéthylamine, la N,N-diméthyléthanolamine, le tris(hydroxy-méthyl)aminométhane, l'éthanolamine, la pyridine, la picoline, la dicyclohexyl-amine, la morpholine, la proceïne, la lysine, l'arginine, l'histidine, la N-méthylglucamine ou encore les sels de phosphonium tels que les sels d'alkyl-phosphonium, les sels d'aryl-phosphonium, les sels d'alkyl-aryl-phosphonium, les alkényl-aryl-phosphonium ou les sels d'ammonium quaternaires tels que les sels de tétra-n-butyl-ammonium.

8. Composé sélénié selon l'une des revendications 1 à 7, caractérisé en ce que ledit composé est choisi parmi le groupe consistant de :
l'ester méthylique de l'acide 2-hydroxy-3-(méthylséléno)propanoïque ;
l'ester méthylique de l'acide (*R*)-2-hydroxy-3-(méthylséléno)propanoïque ;
l'ester méthylique de l'acide (*S*)-2-hydroxy-3-(méthylséléno)propanoïque ;
l'ester éthylique de l'acide 2-hydroxy-3-(méthylséléno)propanoïque ;
l'ester *tert*-butylique de l'acide 2-hydroxy-3-(méthylséléno)propanoïque ;
l'ester benzylique de l'acide (*S*)-(2-hydroxy-3-(méthylséléno)propanoïque ;
l'ester benzylique de l'acide (*S*)-(2-hydroxy-3-(méthylséléno)propanoïque ;
l'ester méthylique de l'acide 3-(éthylséléno)-2-hydroxypropanoïque ;
l'ester méthylique de l'acide 2-hydroxy-3-(isobutylséléno)propanoïque ;
l'ester isopropyle de l'acide 2-hydroxy-3-(méthylséléno)propanoïque ;
l'acide 2-hydroxy-3-(méthylséléno)propanoïque ;
l'acide (*R*)-2-hydroxy-3-(méthylséléno)propanoïque ;
l'acide (S)-2-hydroxy-3-(méthylséléno)propanoïque ;
l'acide 3-éthylséléno-2-hydroxypropanoïque ;
l'acide 2-hydroxy-3-(isobutylséléno)propanoïque ;
le sel dicyclohexylammonium 2-hydroxy-3-(méthylséléno)propanoate ;
le sel de sodium 2-hydroxy-3-(méthylséléno)propanoate ;
le sel de magnesium bis(2-hydroxy-3-(méthylséléno)propanoate ;
le sel du zinc bis(2-hydroxy-3-(méthylséléno)propanoate ;
le sel du calcium bis(2-hydroxy-3-(méthylséléno)propanoate ;
le 2-hydroxy-3-(méthylséléno)propanamide ;
le N-cyclopropyl-2-hydroxy-3-(méthylséléno)propanamide ;
le N-[2-(diméthylamino)éthyl]-2-hydroxy-3-(méthylséléno)propanamide ;
la 2-hydroxy-3-(méthylséléno)-1-(pyrrolidin-1-yl)propan-1-one ;
la 2-hydroxy-3-(méthylséléno)-1-(pipéridin-1-yl)propan-1-one ;
la 2-hydroxy-3-(méthylséléno)-1-(morpholin-4-yl)propan-1-one ;
le N,N-diéthyl-2-hydroxy-3-(méthylséléno)-propanamide ;
le 2-hydroxy-N-(2-hydroxyéthyl)-3-(méthylséléno)-propanamide ;
l'ester *tert*-butylique du [(2*RS*)-2-hydroxy-3-méthylsélénopropanoyl]-(*S*)-alanine ;
la [(2*RS*)-2-hydroxy-3-méthylsélénopropanoyl]-(*S*)-alanine ;
l'ester méthylique du [(2*RS*)-2-hydroxy-3-méthylsélénopropanoyl]-(*S*)-méthionine ;
la [(2*RS*)-2-hydroxy-3-méthylsélénopropanoyl]-(*S*)-méthionine ;
l'ester méthylique de la [(2*R*)-2-hydroxy-3-méthylsélénopropanoyl]-(*S*)-méthionine ;
la [(2*R*)-2-hydroxy-3-méthylsélénopropanoyl]-(*S*)-méthionine ;
l'ester méthylique de la [(2*S*)-2-hydroxy-3-méthylsélénopropanoyl]-(*S*)-méthionine ;
la [(2*S*)-2-hydroxy-3-méthylsélénopropanoyl]-(*S*)-méthionine ;

l'ester méthylique de la N(α)-[(2*RS*)-2-hydroxy-3-méthylsélénopropanoyl]-N(ω)-*tert*-butoxycarbonyl-(*S*)-lysine ;

l'ester méthylique de la N(α)-[(2*RS*)-2-hydroxy-3-méthylsélénopropanoyl]-N(ω)-fluorenylméthyloxycarbonyl-(*S*)-lysine ;

l'ester méthylique de la N(α)-[(2*RS*)-2-hydroxy-3-méthylsélénopropanoyl]-N(ω)-benzyloxycarbonyl-(*S*)-lysine ;

l'ester méthylique de la [(2*RS*)-2-hydroxy-3-méthylsélénopropanoyl]-(*S*)-sélénométhionine ;

la [(2*RS*)-2-hydroxy-3-méthylsélénopropanoyl]-(*S*)-sélénométhionine ;

l'ester méthylique de la [(2*R*)-2-hydroxy-3-méthylsélénopropanoyl]-(*S*)-sélénométhionine ;

la [(2*R*)-2-hydroxy-3-méthylsélénopropanoyl]-(*S*)-sélénométhionine ;

l'ester méthylique de la [(2*S*)-2-hydroxy-3-méthylsélénopropanoyl]-(*S*)-sélénométhionine ;

la [(2*S*)-2-hydroxy-3-méthylsélénopropanoyl]-(*S*)-sélénométhionine ;

l'acide 2-(acétyloxy)-3-(méthylséléno)propanoïque ;

l'acide 2-(dodécanoyloxy)-3-(méthylséléno)propanoïque ;

l'ester méthylique de l'acide 2-(benzoyloxy)-3-(méthylséléno)propanoïque ;

l'acide 2-(benzoyloxy)-3-(méthylséléno)propanoïque ;

l'ester méthylique de l'acide 3-(méthylséléno)-2-[(3'-pyridine)oxycarbonyl]propanoïque ;

l'ester méthylique de l'acide 2-[(*tert*-butoxycarbonyl)oxy]-3-(méthylséléno)propanoïque ;

l'acide 2-[(*tert*-butoxycarbonyl)oxy]-3-(méthylséléno)propanoïque ;

l'ester méthylique de l'acide (2*RS*)-[N-(*tert*-butoxycarbonyl)-*S*-methionyl]-3-(méthylséléno)propanoïque ;

l'ester méthylique de l'acide 4-méthylséléno-2-(2'-acétyloxy-3'-méthylsélénopropanoyl)butyrique ;

l'acide 4-méthylséléno-2-(2'-acétyloxy-3'-méthylsélénopropanoyl)butyrique ;

l'ester méthylique de l'acide 3-méthylséléno-2-(2'-acétyloxy-4'-méthylsélénobutanoyl)propanoïque ;

l'acide 3-méthylséléno-2-(2'-hydroxy-4'-méthylsélénobutanoyl)-propanoïque ;

l'acide 2-(pentanoyloxy)-3-(méthylséléno)-propanoïque ;

l'acide 2-(nonanoyloxy)-3-(méthylséléno)-propanoïque ;

l'acide 2-(linoleoyloxy)-3-(méthylséléno)-propanoïque ;

l'ester méthylique de l'acide 2-(linoleoyloxy)-3-(méthylséléno)-propanoïque ;

l'acide 2-(pivaloyloxy)-3-(méthylséléno)-propanoïque ;

l'acide 2-(3-chloropropanoyloxy)-3-(méthylséléno)-propanoïque ;

l'acide 2-{(1H-imidazoyl-4-ylcarbonyl)oxy)}-3-(méthylséléno)-propanoïque ;
l'ester méthylique de l'acide 2-(pivaloyloxy)-3-(méthylséléno)-propanoïque ;

9. Procédé de préparation des composés séléniés de formule générale (I), défini à l'une quelconque des revendications 1 à 7, caractérisé en ce qu'il comprend les étapes suivantes :

1) la réaction d'un ester de l'acide oxirane-2-carboxylique racémique (DL) ou de l'un de ses énantiomères (D ou L) disponibles commercialement par exemple chez SAF France, avec

a/ soit un alkylsélénol R^1SeH, lui-même préparé *in situ* à partir d'un sel alcalin d'alkylsélénolate de formule R^1-Se-M_1 obtenu lui-même par réduction du dialkyle disélénure (dans laquelle M_1 représente un atome de métal alcalin), mis en réaction avec du chlorure d'ammonium;

b/ soit avec un dérivé dialkylaluminium alkylsélénolate de formule $Al(R^1)_2SeR^1$, lui-même généré *in situ* à partir du trialkylaluminium $Al(R^1)_3$ correspondant et du sélénium élémentaire Se°.

2) le cas échéant une ou plusieurs des réactions ou séries de réactions suivantes (voir Figure 1):

- hydrolyse de la fonction ester, puis
- acidification du milieu réactionnel pour obtenir les acides correspondants de formule (I) où X = OH; puis
- estérification des acides de formule (I) ou de leurs sels alcalins avec un alcool ou un halogénure d'alkyle pour obtenir les esters correspondants de formule générale (I) dans laquelle X = OR^3, avec R^3 tel que défini précédemment;
- amidification des acides de formule (I) avec une amine appropriée de formule R^4R^5NH, ou NH_3 dans laquelle R^5 est tel que défini précédemment, pour obtenir le composé de formule générale (I) dans laquelle X = NH_2, NR^4R^5 ou α-amino-acide, $CH_3SeCH_2CH_2CH(COOH)NH$- ,
- estérification, lorsque R^2 = H, de la fonction hydroxyle par un acide aproprié pour obtenir le composé de formule générale (I) dans laquelle OR^2 est différent du groupe OH ;
- salification par un acide ou par une base.

10. Procédé selon la revendication 9, caractérisé en ce que le réactif sélénié est :

* soit un dialkylaluminium alkylsélénolate, et plus particulièrement le diméthyle aluminium méthylsélénolate généré *in situ* à partir de sélénium métal Se° et du triméthyle aluminium $Al(CH_3)_3$ dans un solvant polaire aprotique.

* soit un alkylsélénol, généré *in situ* à partir de sélénium métal Se(0) et d'alkyle lithium, dans un solvant polaire aprotique, puis mis en présence de chlorure d'ammonium.

11. Procédé selon la revendication 9 ou 10, caractérisé en ce que la réaction 1) a lieu au sein d'un solvant polaire aprotique tel que par exemple le THF et en ce que les réactions subséquentes, conduisant aux différents composés de formule (I), comprennent au moins une acidification, ou une estérification, ou une amidification et salification, dans des conditions bien connues de l'homme du métier.

12. Utilisation d'au moins un composé sélénié de formule (I) tel que défini à l'une des revendications 1 à 8, comme agent pharmaceutique, en particulier comme agent anti-tumoral, seul ou associé à au moins un autre agent pharmaceutique et notamment à au moins un autre agent anti-tumoral.

13. Composition pharmaceutique caractérisée en ce qu'elle comprend au moins un principe pharmaceutiquement actif comprenant au moins un composé sélénié de formule générale (I), tel que précédemment défini, seul ou associé avec au moins un autre principe pharmaceutiquement actif.

14. Composition pharmaceutique selon la revendication 13, caractérisée en ce que le composé sélénié de formule générale (I), constitue un principe pharmaceutiquement actif pour réaliser la prévention et le traitement de tumeurs ou cancers, soit seul soit en association avec un ou plusieurs autres agents anti-cancéreux ou cytotoxiques connus, soit en pré-administration soit en co-administration, tels que ceux notamment de la prostate, du foie, du rein, du pancréas, du poumon, du colon et de la peau.

15. Composition pharmaceutique selon la revendication 13 ou 14, caractérisée en ce que au moins un des autres agents anticancéreux soit choisi parmi les composés suivants : l'aminoglutéthimide, l'estramustine, le medroxyprogesteroneacetate, le leuprolide, le flutamide, le torémifène, le Zoladex, les inhibiteurs de métalloprotéinase de matrice; les inhibiteurs du VEGF, tels que les anticorps anti-VEGF (Avastin (R)) et les petites molécules comme le ZD6474 et le SU6668; le

vatalanib, le BAY-43-9006, le SU11248, le CP-547632 et le CEP-7055; les SA-1 et SA-2 inhibiteurs y compris les anticorps anti-HER2 (Herceptin), les inhibiteurs de l'EGFR, y compris le gefitinib, l'erlotinib, l'ABX-EGF, l'EMD72000, le 11F8, et le cetuximab ; les inhibiteurs d'Eg5, tels que le SB-715992, le SB-743921, et le MKI-833 ; les inhibiteurs de PAN, tels que le canertinib, l'EKB-569, le CI-1033, l'AEE-788, le XL-647 ; les inhibiteurs de kinase, tels que le 2C4, et le GW-572016, le Gleevec (R) et le dasatinib (Sprycel (R)); le Casodex (R) (bicalutamide, Astra Zeneca), le tamoxifène; les inhibiteurs de MAPK kinase, les inhibiteurs de PI3 kinase, les inhibiteurs de PDGF, tels que l'imatinib; les anti-angiogéniques et les agents anti-vasculaires; les inhibiteurs de non-récepteurs et récepteurs tyrosine kinases, les inhibiteurs de la signalisation de l'intégrine; la tubuline ; les agents tels que la vinblastine, la vincristine, la vinorelbine, la vinflunine, le paclitaxel, le docétaxel, le 7-O-methylthiomethylpaclitaxel, le 4-désacétyl-4-methylcarbonatepaclitaxel, le C-4 méthyl carbonate paclitaxel, l'epothilone A, l'epothilone B, l'épothilone C, l'epothilone D, la désoxyépothilone A, la désoxyépothilone B, l'oxabicyclo [14.1.0] le heptadécane-5-9-dione (ixabepilone), la leucovorine, le tégafur et des dérivés de ceux-ci; les inhibiteurs de CDK, les inhibiteurs du cycle cellulaire anti-prolifératifs, l'epidophyllotoxin, l'étoposide, le VM-26; les enzymes antinéoplasiques, les inhibiteurs de topoisomérase I et II tels que la camptothécine, le topotécan, la SN-38, la procarbazine, la mitoxantrone ; les complexes de coordination du platine tels que le cisplatine, le carboplatine et oxaliplatine ; les antagonistes de la purine tels que la 6-thioguanine et la 6-mercaptopurine; les antagonistes de la glutamine.

16. Composition pharmaceutique selon la revendication 13, 14 ou 15, caractérisée en ce que au moins un des autres agents cytotoxiques soit choisi parmi au moins un composé suivant: le cyclophosphamide, la doxorubicine, la daunorubicine, la mitoxantrone, le melphalan, l'hexaméthyle mélamine, le thiotépa, la cytarabine, l'idatrexate, le trimétrexate, la dacarbazine, la L-asparaginase, le bicalutamide, le leuprolide, les dérivés de pyridobenzoindole, les interférons, les interleukines.

17. Composition pharmaceutique selon l'une des revendications 13, 14, 15 ou 16, caractérisée en ce qu'elle comprend au moins un composé sélénié de formule (I) à une concentration comprise entre 0,02% et 0,15% en poids équivalent sélénium.

18. Composition pharmaceutique selon l'une des revendications 13, 14, 15 ou 16, caractérisée en ce qu'elle comprend au moins un support pharmaceutiquement acceptable et une quantité thérapeutiquement efficace d'un ou plusieurs des composés sélénies ou d'un stéréoisomère, d'un tautomère, ou d'un sel pharmaceutiquement acceptable. Ces milieux peuvent notamment consister en :

* une solution injectable ou buvable,

* un milieu solide composé d'un ou plusieurs excipients pouvant être sélectionnés parmi des vitamines, des antioxydants naturels tel que la L-ergothionéine, des sels minéraux, des mono-, di- ou polysaccharides, notamment l'acide folique, les vitamines B_6, E ou C, du lactose, de l'amidon.

19. Composition pharmaceutique selon l'une des revendications 13 à 18, caractérisée en ce qu'elle est formulée pour une voie d'administration choisie parmi la voie orale, intraveineuse, parentérale, topique incluant la voie transdermique ou la voie nasale ou la voie oculaire, ou par inhalation et en particulier se présenter sous la forme d'une gélule, d'un gel, d'un comprimé ou d'une poudre.

Figure 1 : Schéma du procédé de synthèse des composés selon la formule générale (I)

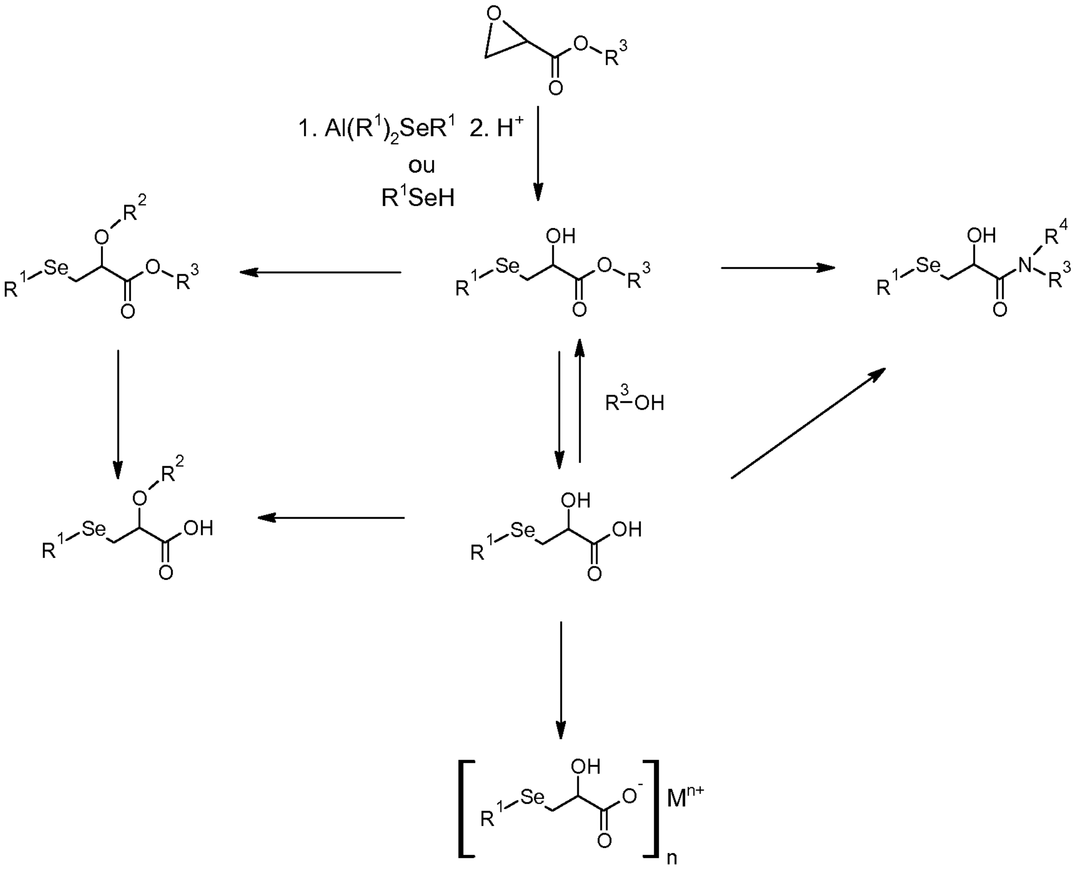

Figure 2 : Pourcentage de viabilité des cellules DU145 et LS174T en fonction de la concentration en composé 4, après 96 heures de traitement

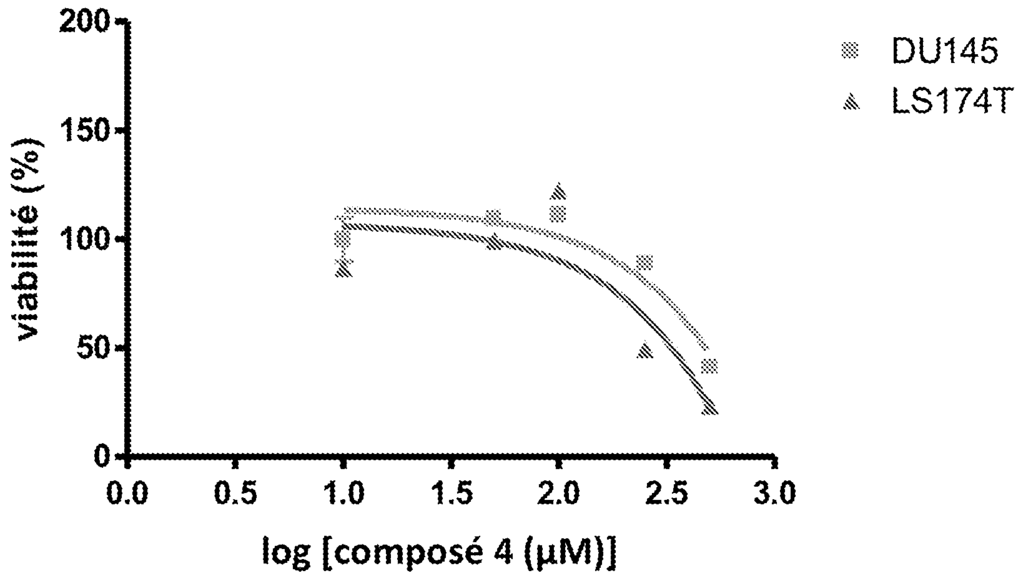

Figure 3 : Pourcentage de viabilité des cellules DU145, LS174T et HT-29 en fonction de la concentration en composé 10, après 96 heures de traitement

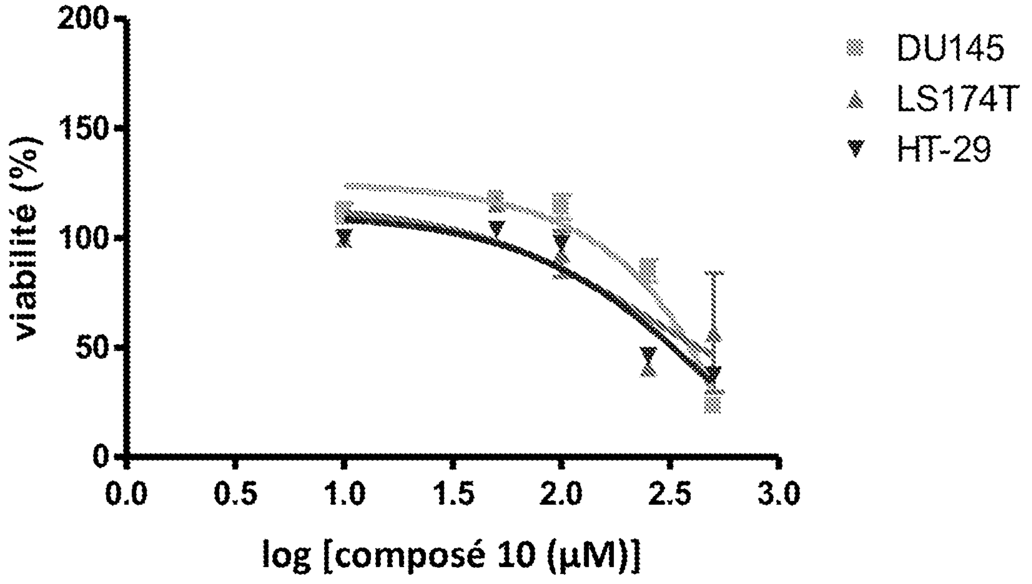

Figure 4 : Pourcentage de viabilité des cellules PC3, DU145, PANC-1 et MIA PaCa-2 en fonction de la concentration en composé 38, après 96 heures de traitement

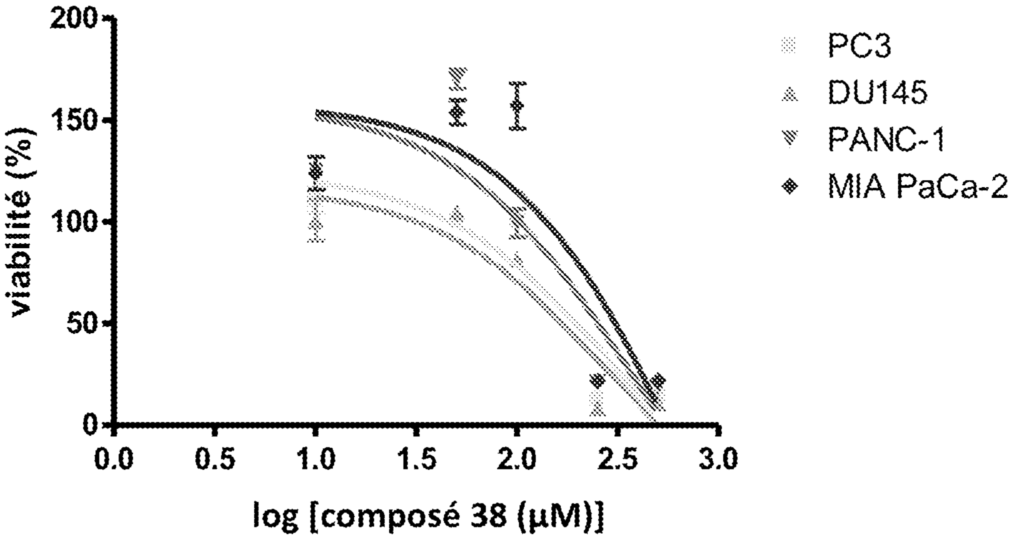

Figure 5 : Pourcentage de viabilité des cellules HT-29, LS174T, HepG2 et MCF-7 en fonction de la concentration en composé 38, après 96 heures de traitement

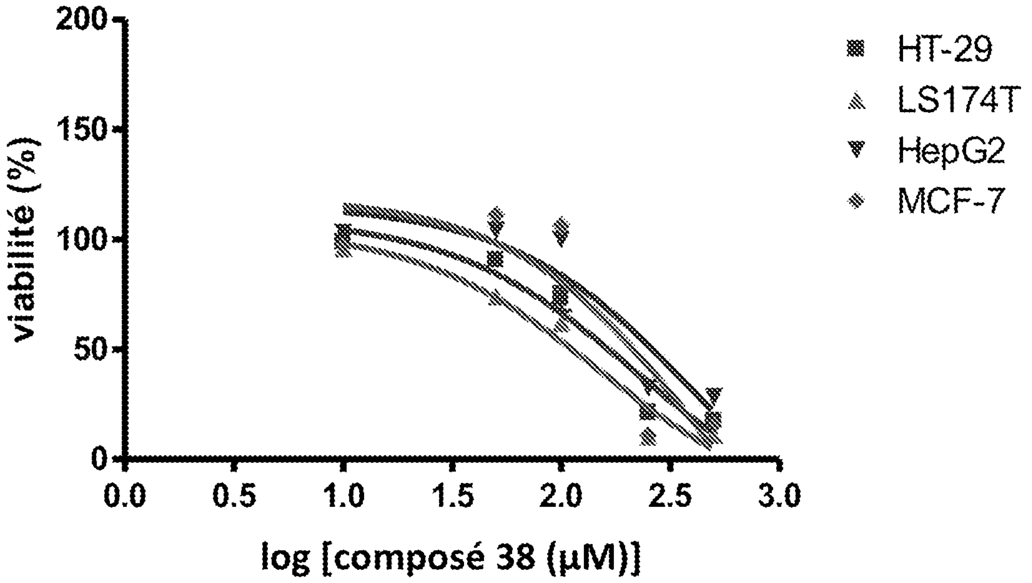

Supplement: Supplementary file 1 [file DataSheet_1.pdf]
