# Supplementary figures and images for: Organic Selenium Reaches the Central Nervous System and Downmodulates Local Inflammation: A Complementary Therapy for Multiple Sclerosis?
Source: Front Immunol. 2020 Oct 30;11:571844. doi: 10.3389/fimmu.2020.571844 (PMC7664308; doi:10.3389/fimmu.2020.571844)

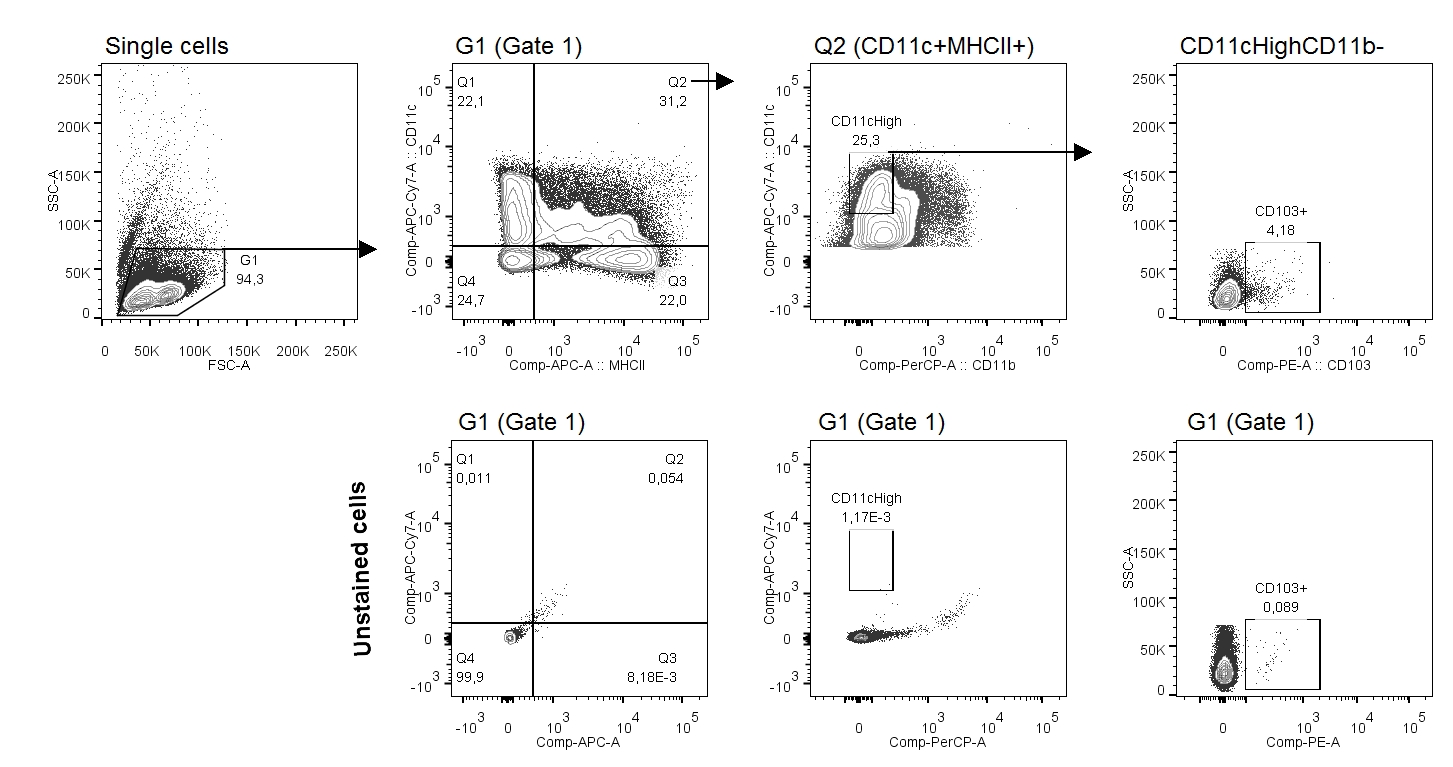

Supplement: Supplementary file 2 [file Image_1.jpeg]

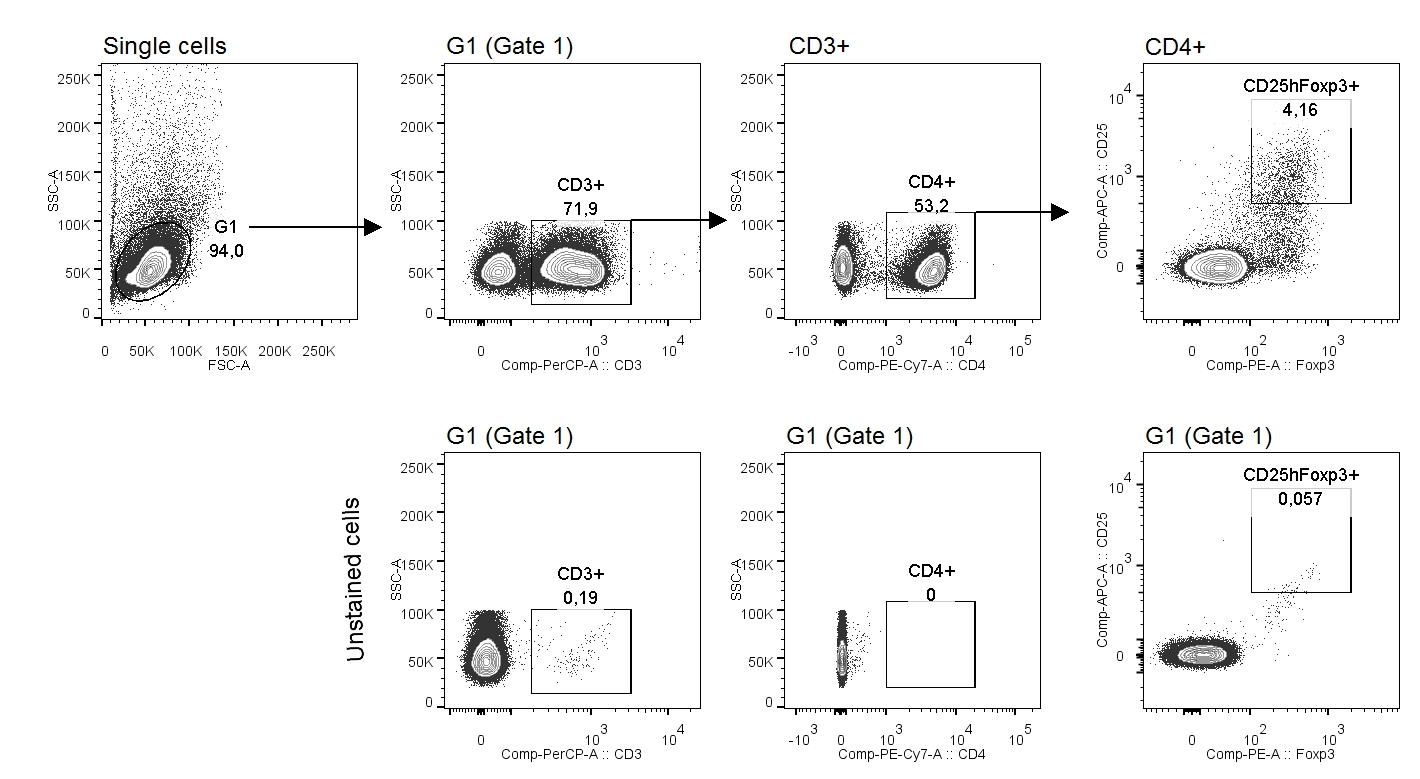

Supplement: Supplementary file 3 [file Image_2.jpeg]
